# Supplementary material for: Just Culture for Medical Students: Understanding Response to Providers in Adverse Events
Source: MedEdPORTAL. 2021 Jul 9;17:11167. doi: 10.15766/mep_2374-8265.11167 (PMC8266940; doi:10.15766/mep_2374-8265.11167)
Supplement: Supplementary file 1 — Slides for Cases.pptxLecture Slides.pptxFaculty Guide.docxQuiz and Evaluation Items.docx [file mep_2374-8265.11167-s001.zip › A. Slides for Cases.pptx]

## Slide 1
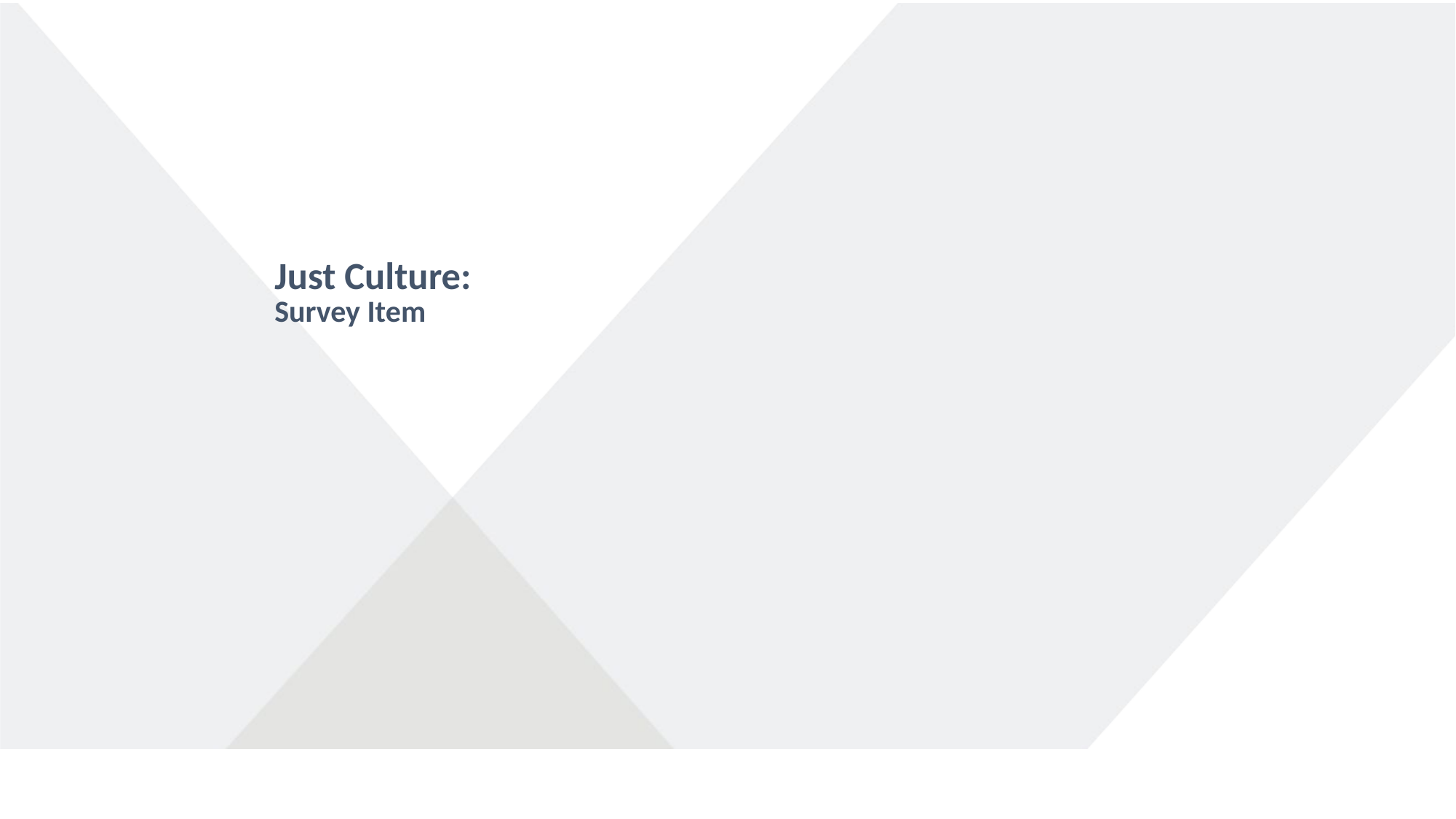

# Just Culture: Survey Item

## Slide 2
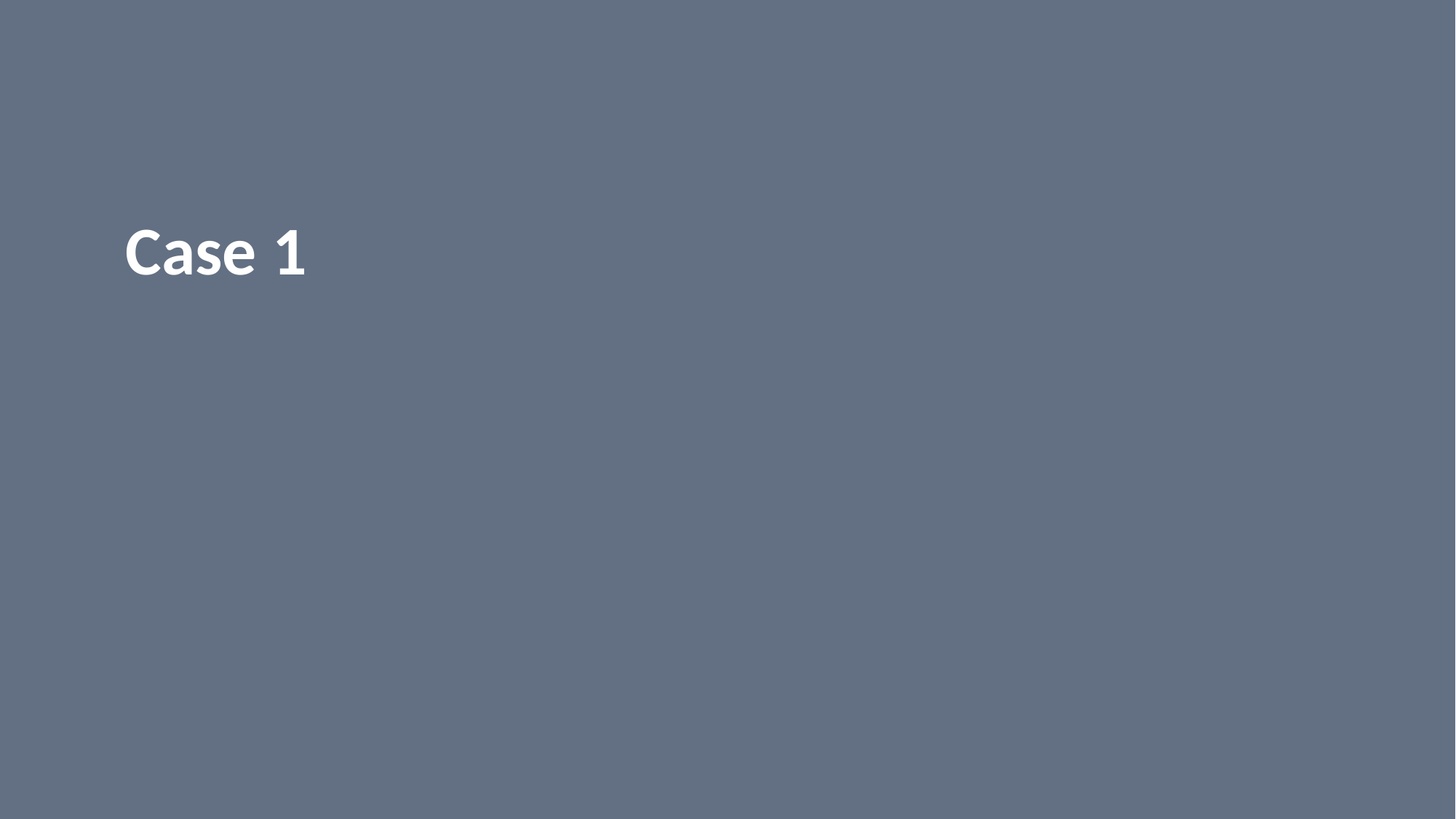

# Case 1

## Slide 3
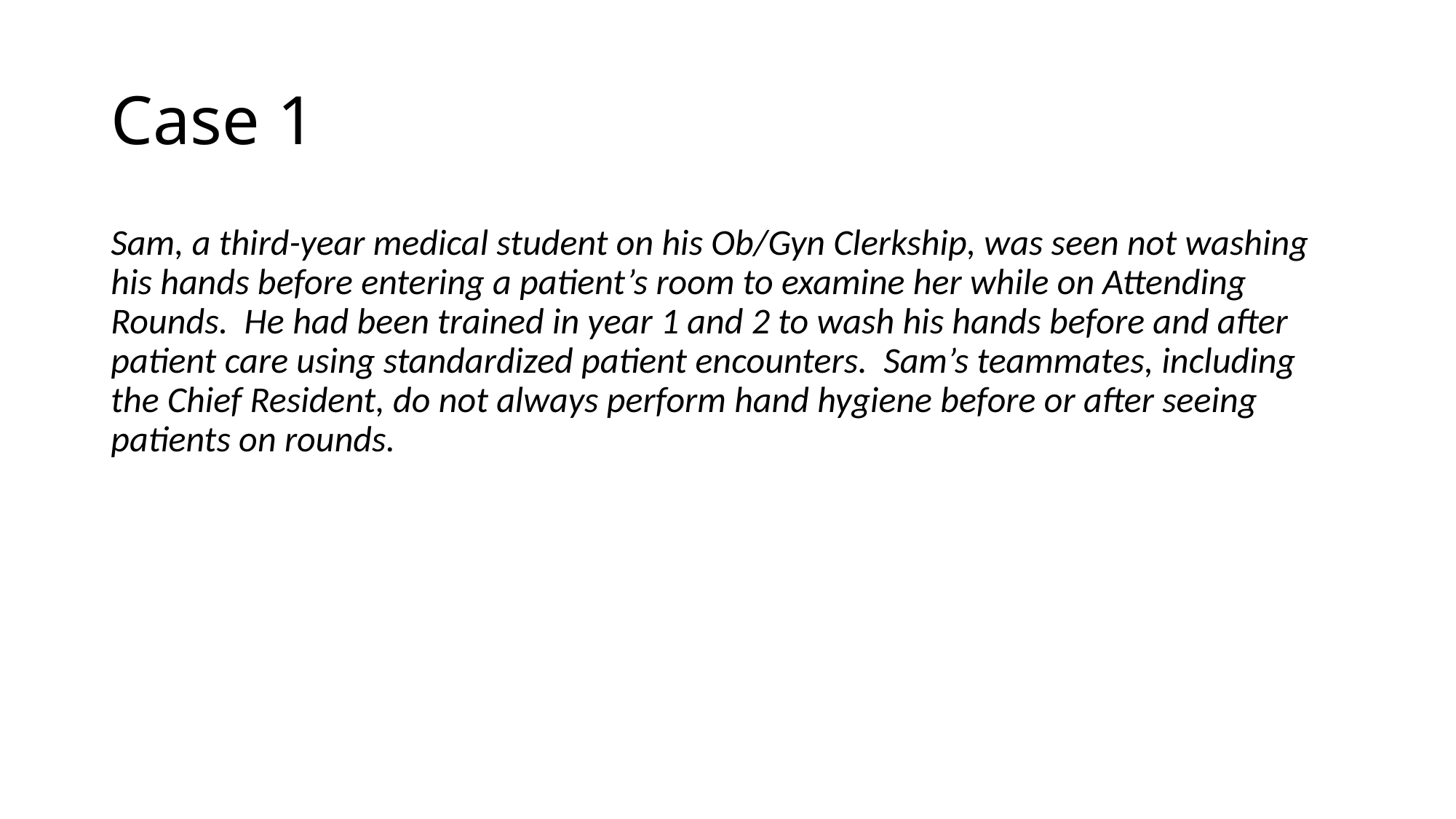

# Case 1
Sam, a third-year medical student on his Ob/Gyn Clerkship, was seen not washing his hands before entering a patient’s room to examine her while on Attending Rounds. He had been trained in year 1 and 2 to wash his hands before and after patient care using standardized patient encounters. Sam’s teammates, including the Chief Resident, do not always perform hand hygiene before or after seeing patients on rounds.

## Slide 4
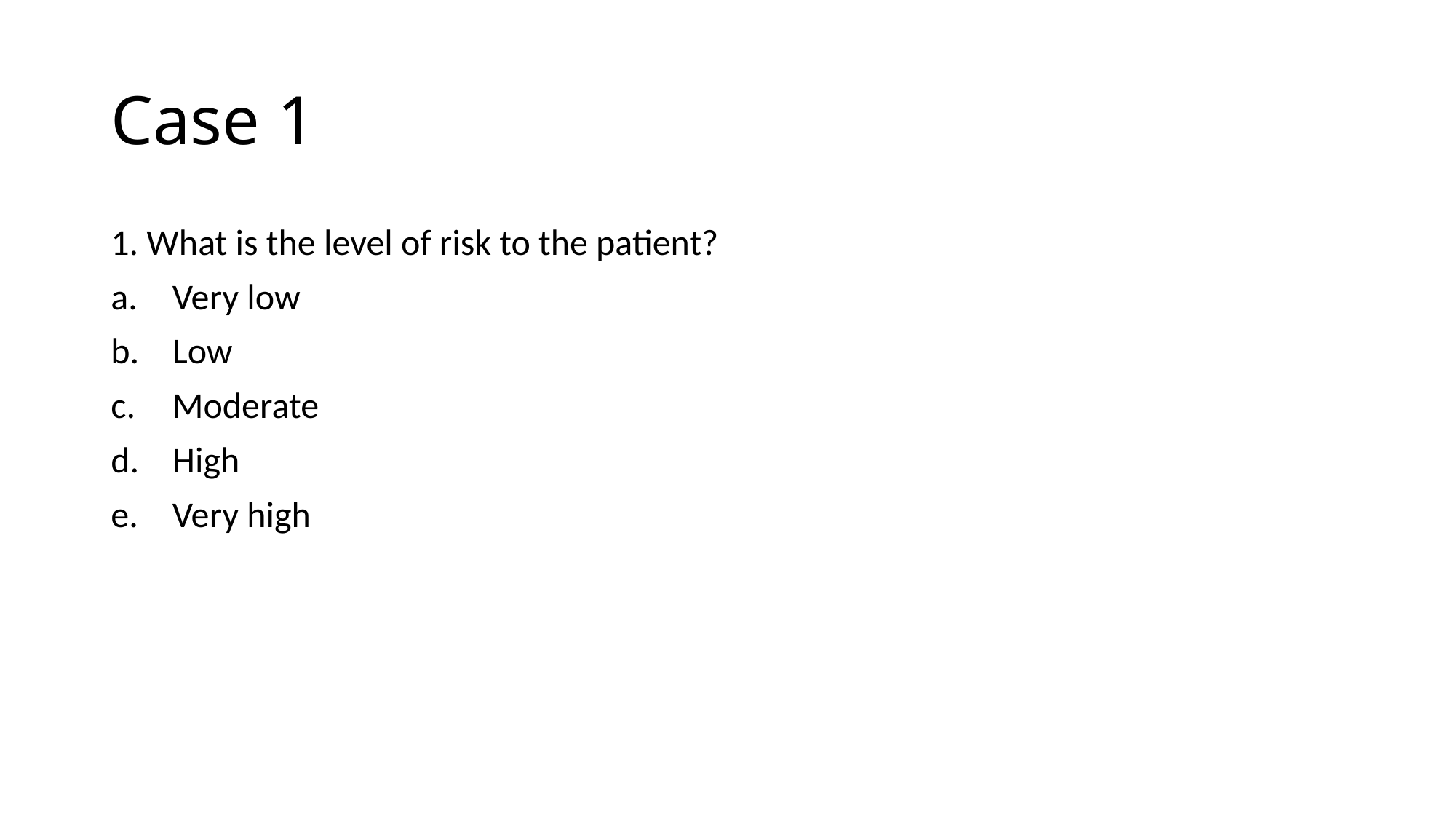

# Case 1
1. What is the level of risk to the patient?
Very low
Low
Moderate
High
Very high

## Slide 5
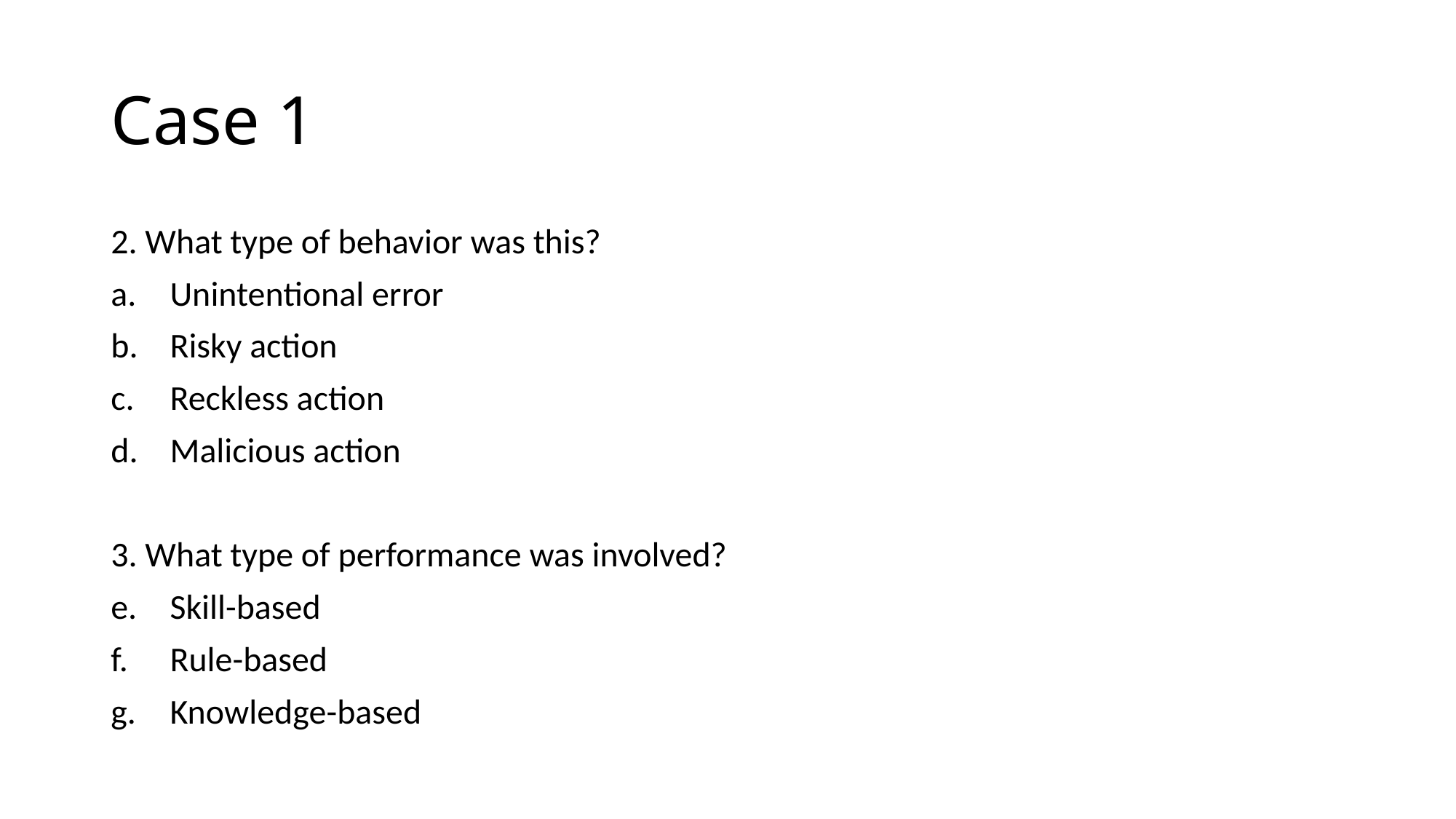

# Case 1
2. What type of behavior was this?
Unintentional error
Risky action
Reckless action
Malicious action
3. What type of performance was involved?
Skill-based
Rule-based
Knowledge-based

## Slide 6
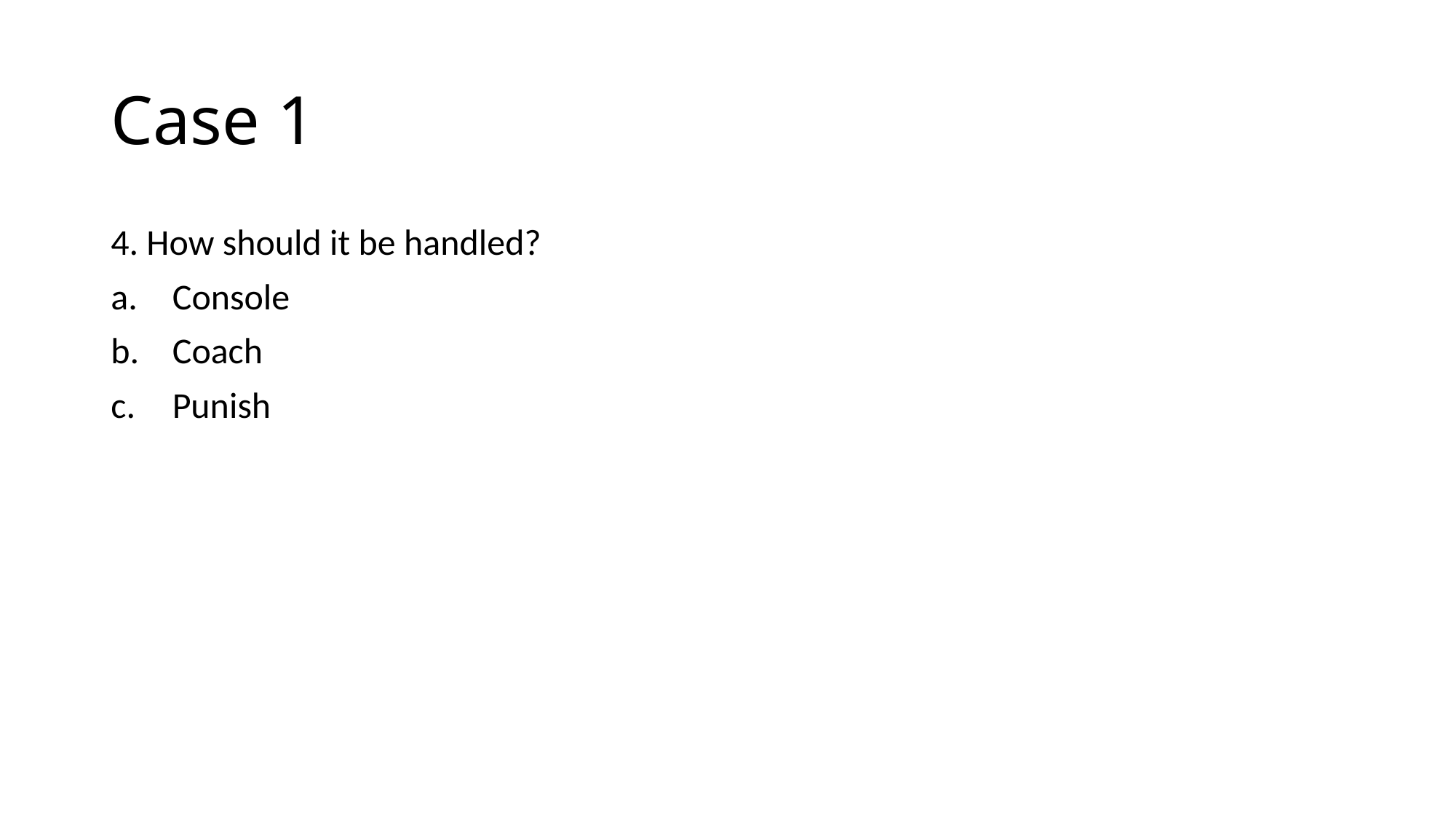

# Case 1
4. How should it be handled?
Console
Coach
Punish

## Slide 7
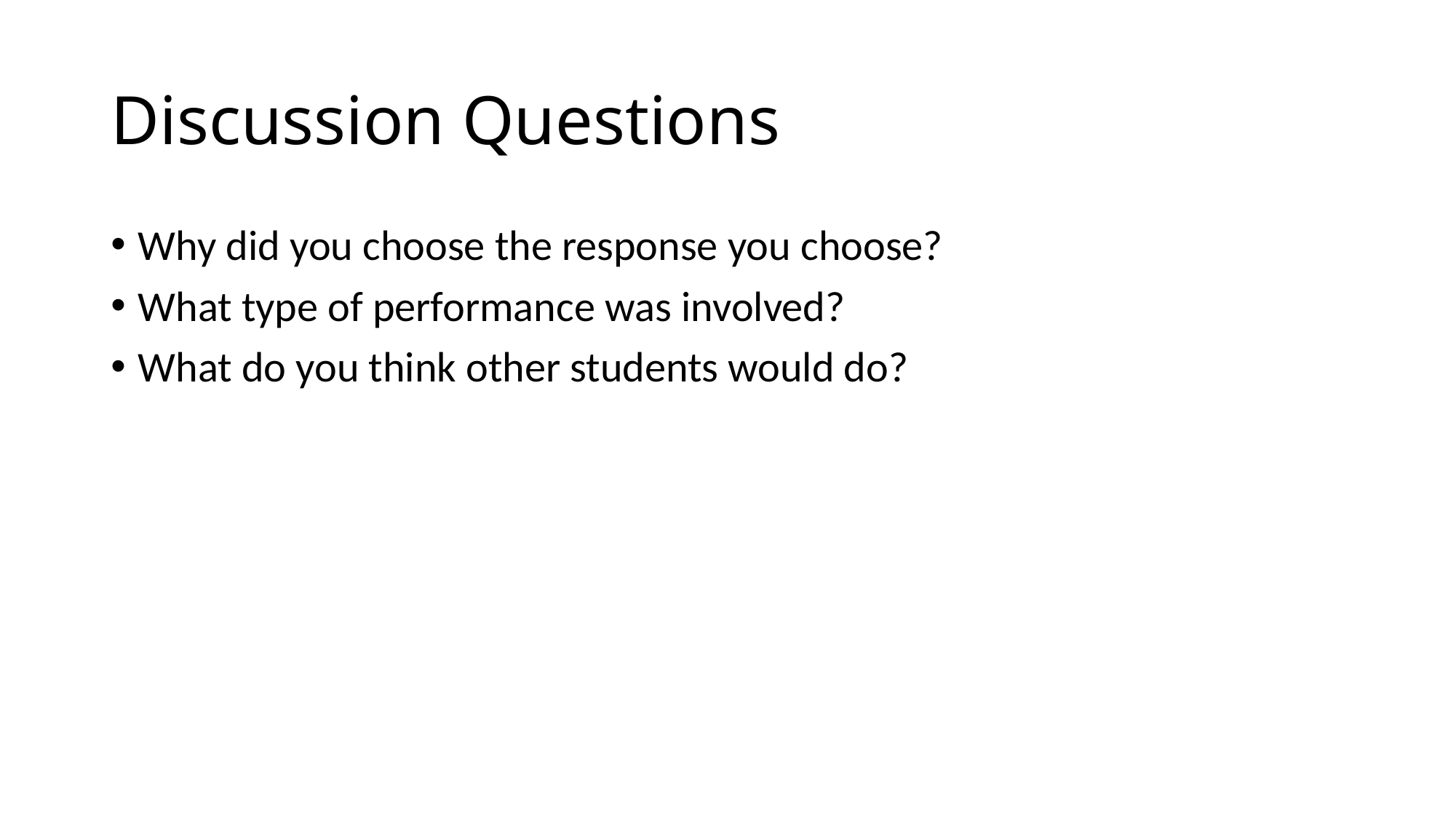

# Discussion Questions
Why did you choose the response you choose?
What type of performance was involved?
What do you think other students would do?

## Slide 8
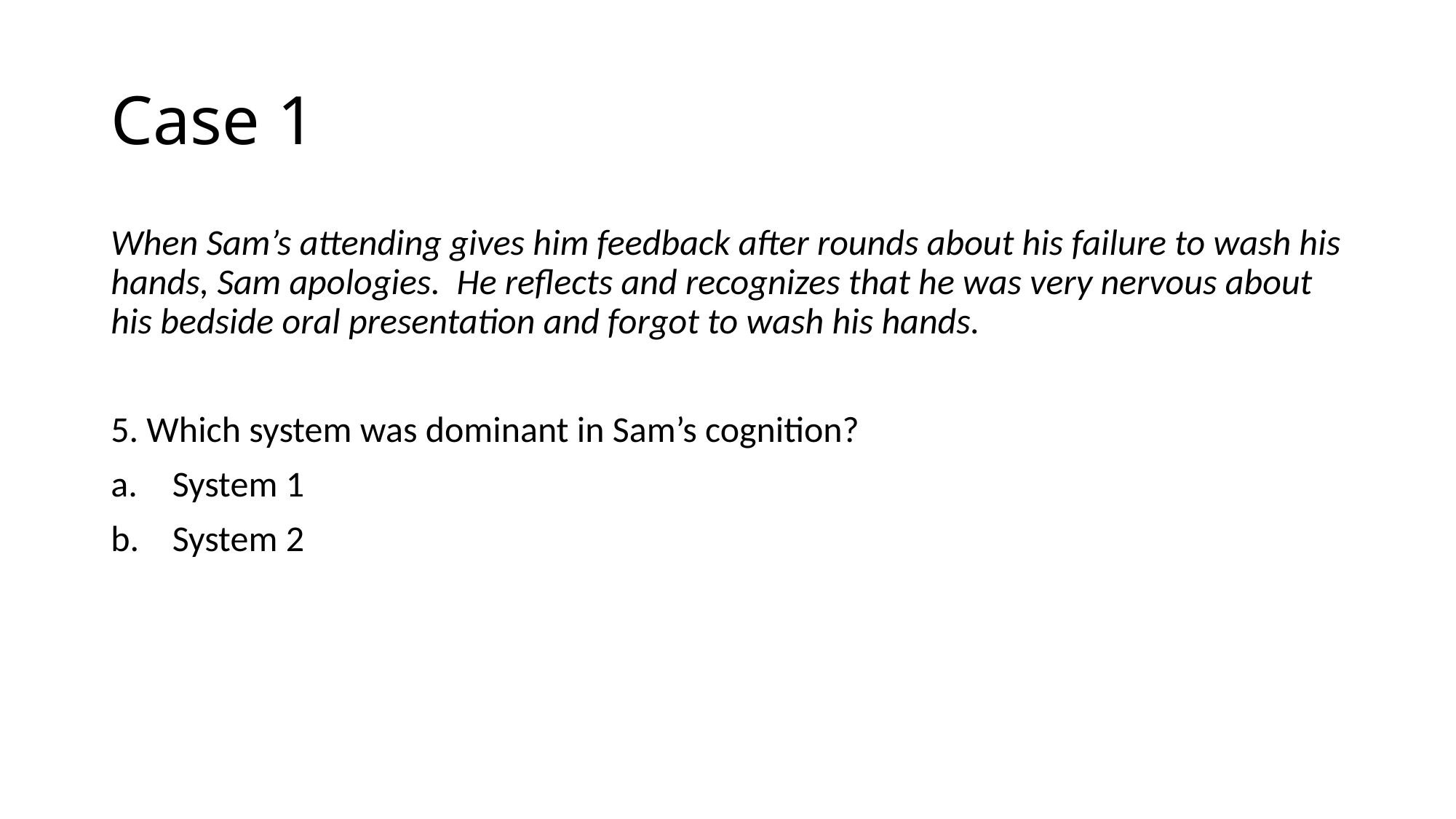

# Case 1
When Sam’s attending gives him feedback after rounds about his failure to wash his hands, Sam apologies. He reflects and recognizes that he was very nervous about his bedside oral presentation and forgot to wash his hands.
5. Which system was dominant in Sam’s cognition?
System 1
System 2

## Slide 9
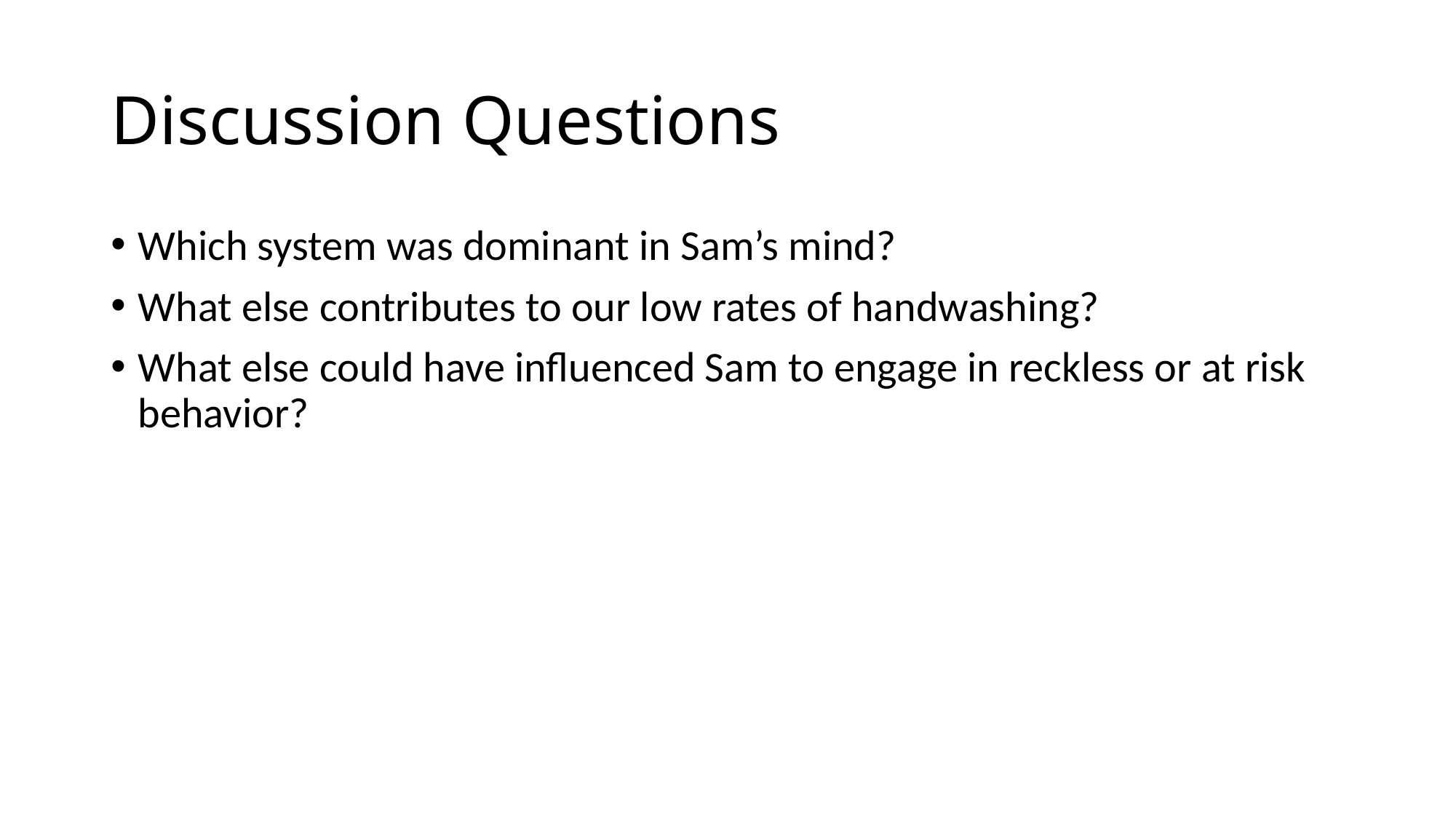

# Discussion Questions
Which system was dominant in Sam’s mind?
What else contributes to our low rates of handwashing?
What else could have influenced Sam to engage in reckless or at risk behavior?

## Slide 10
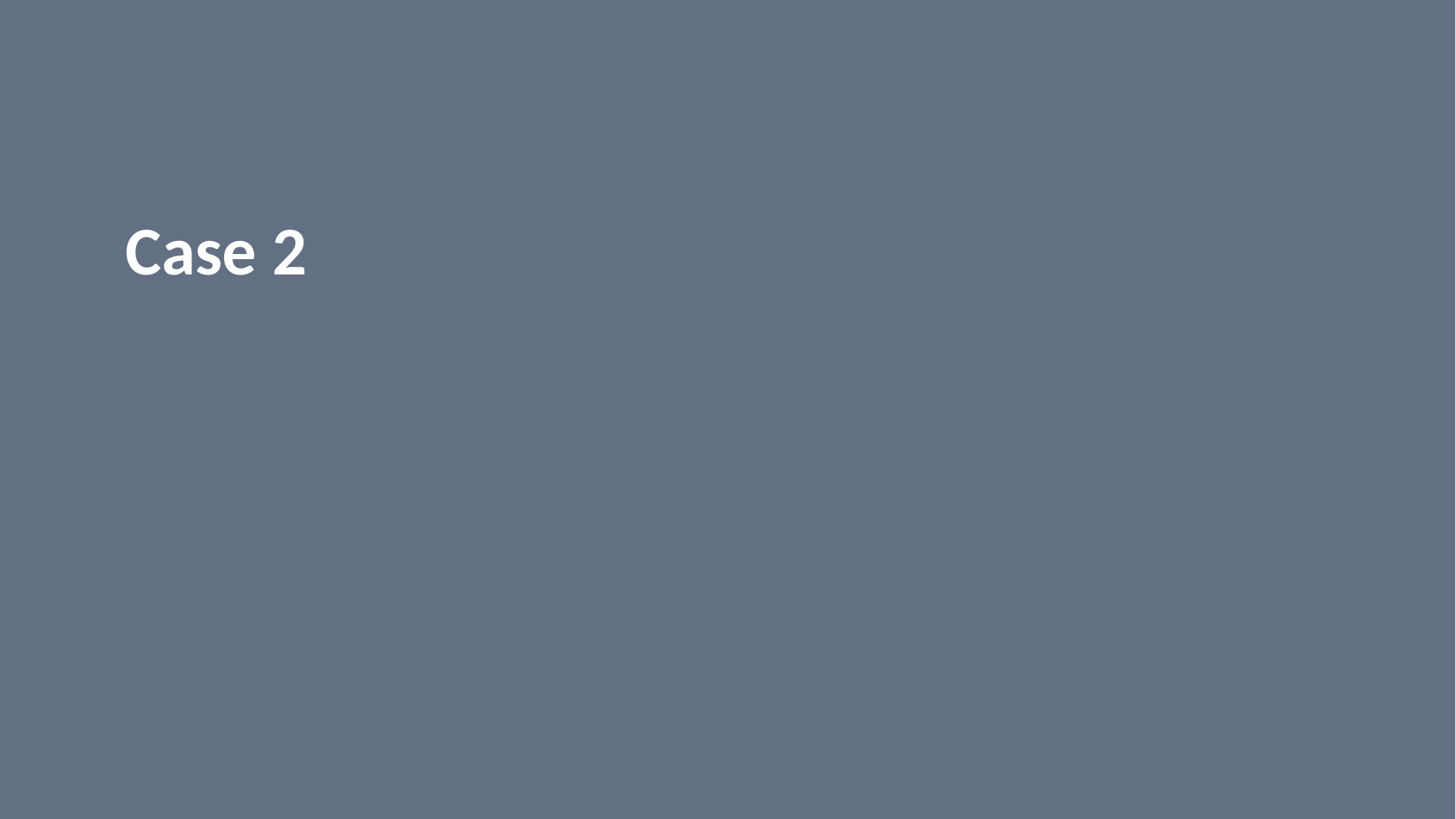

# Case 2

## Slide 11
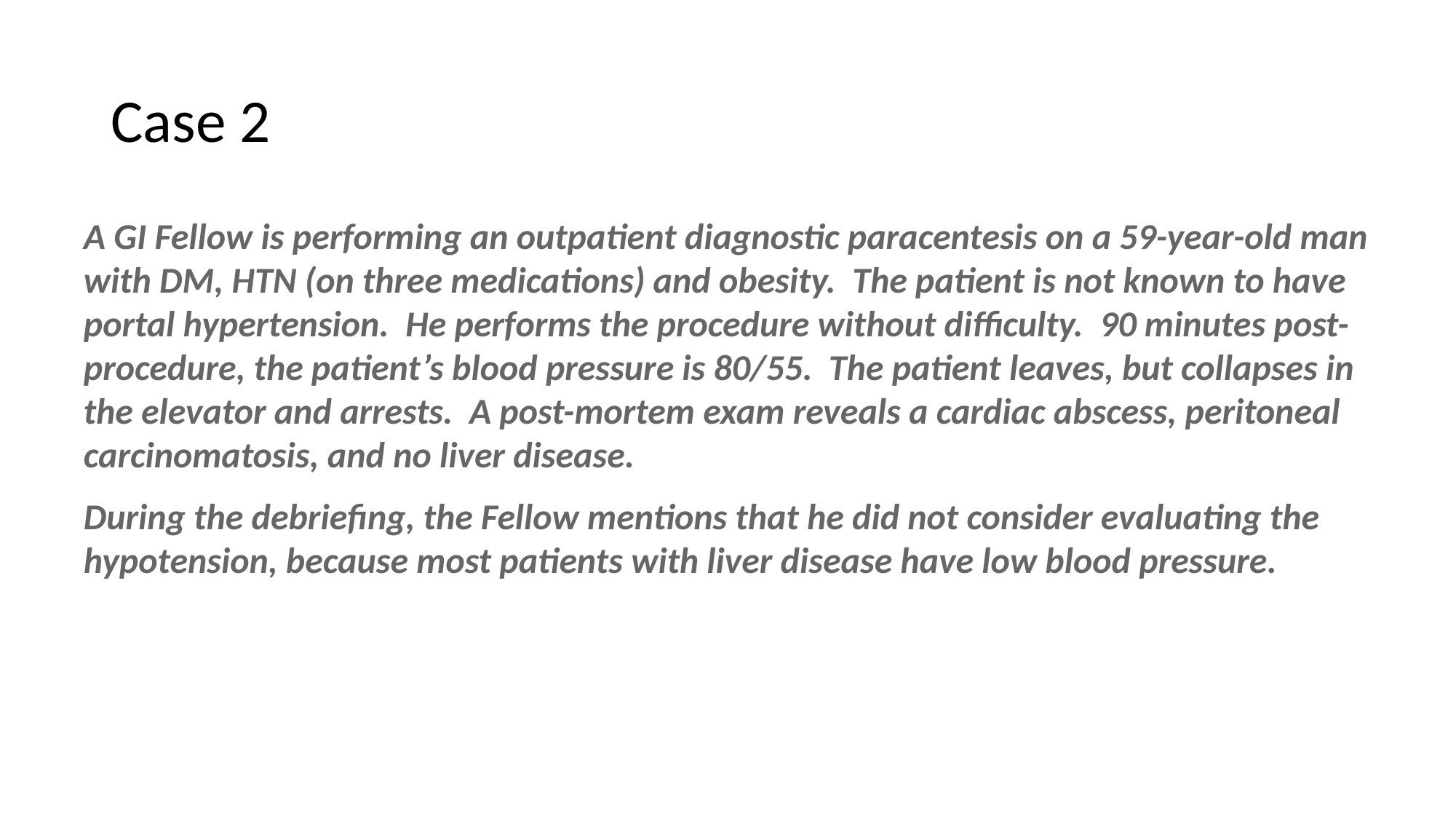

# Case 2
A GI Fellow is performing an outpatient diagnostic paracentesis on a 59-year-old man with DM, HTN (on three medications) and obesity. The patient is not known to have portal hypertension. He performs the procedure without difficulty. 90 minutes post-procedure, the patient’s blood pressure is 80/55. The patient leaves, but collapses in the elevator and arrests. A post-mortem exam reveals a cardiac abscess, peritoneal carcinomatosis, and no liver disease.
During the debriefing, the Fellow mentions that he did not consider evaluating the hypotension, because most patients with liver disease have low blood pressure.

## Slide 12
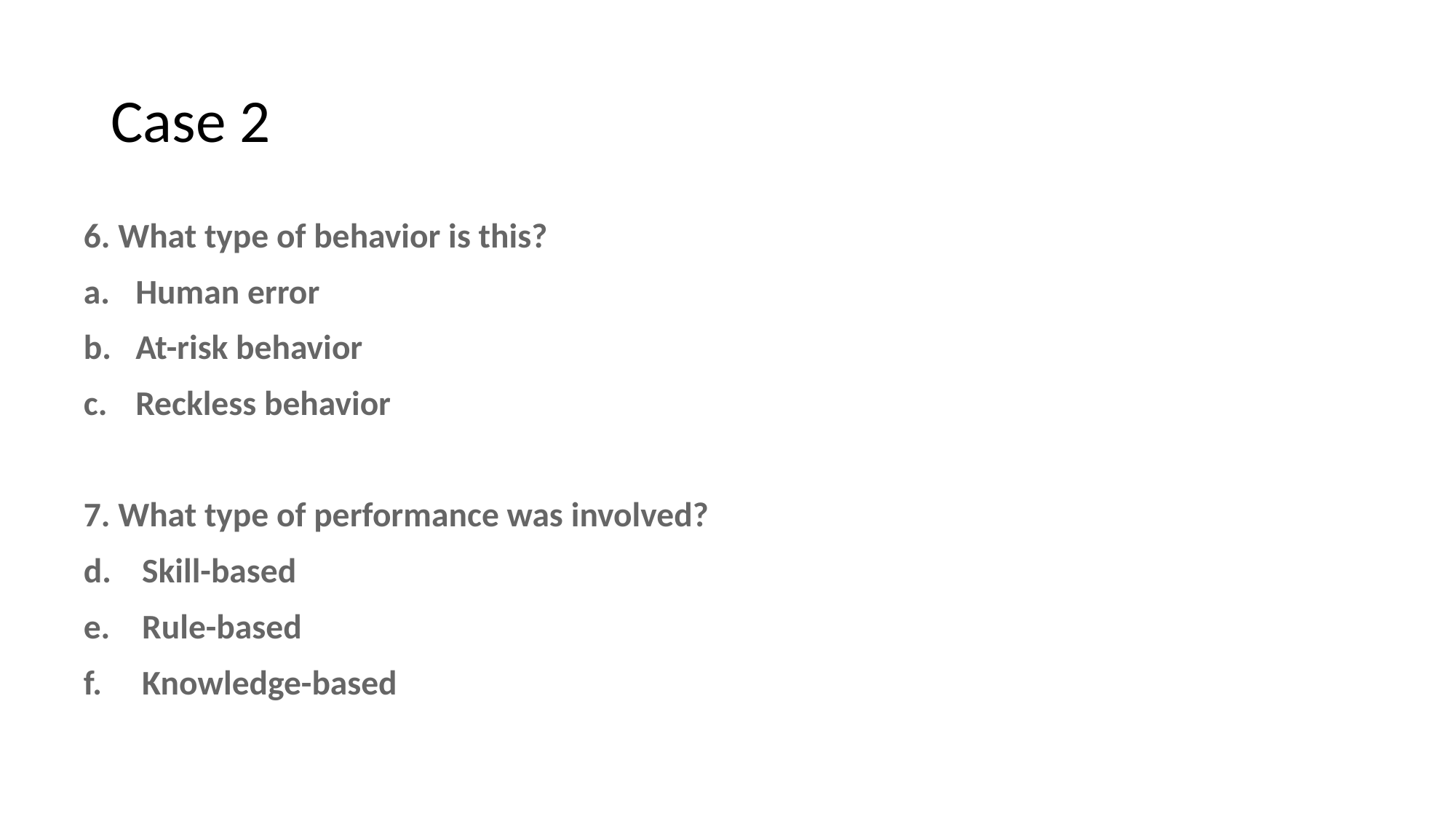

# Case 2
6. What type of behavior is this?
Human error
At-risk behavior
Reckless behavior
7. What type of performance was involved?
Skill-based
Rule-based
Knowledge-based

## Slide 13
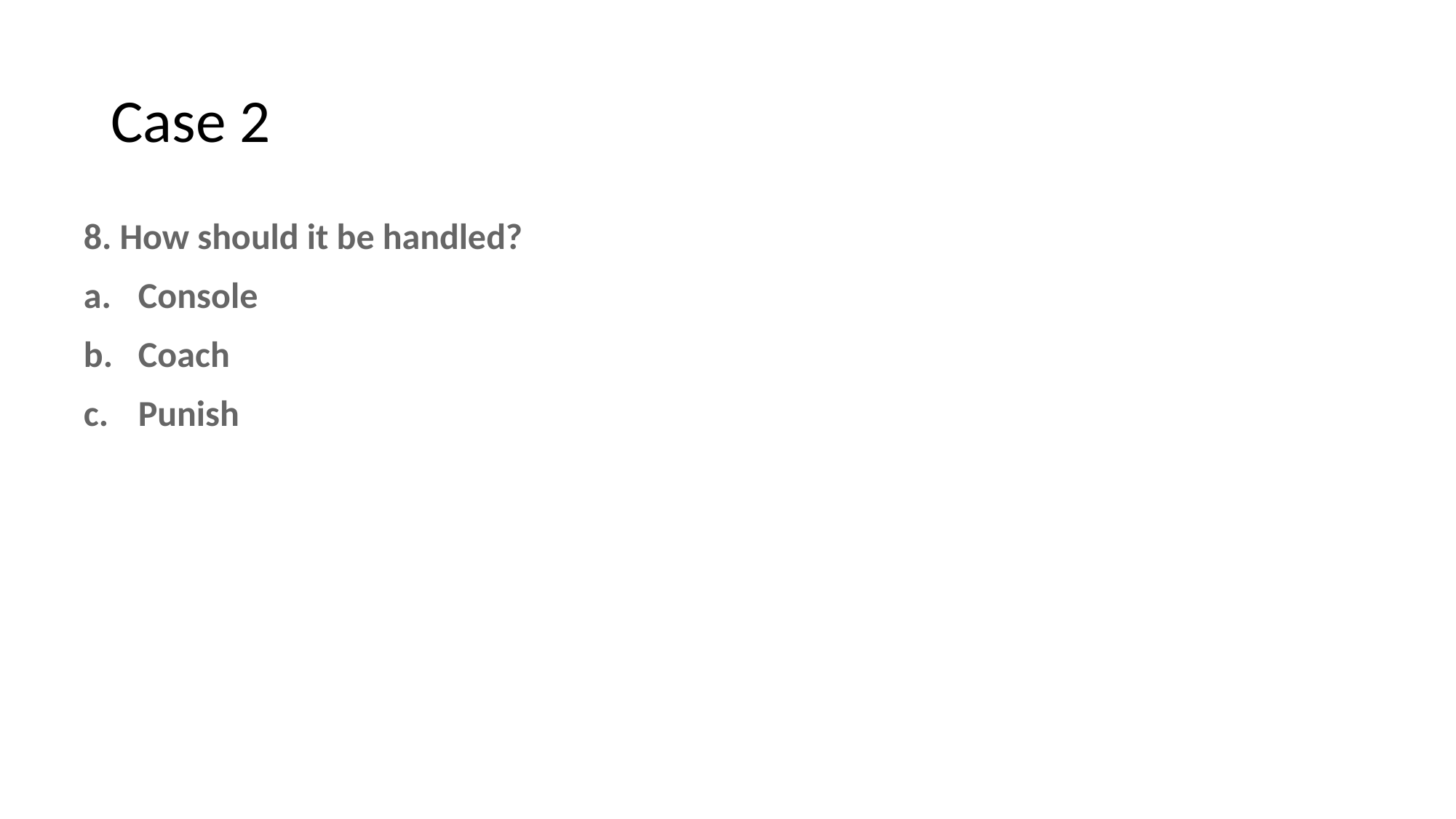

# Case 2
8. How should it be handled?
Console
Coach
Punish

## Slide 14
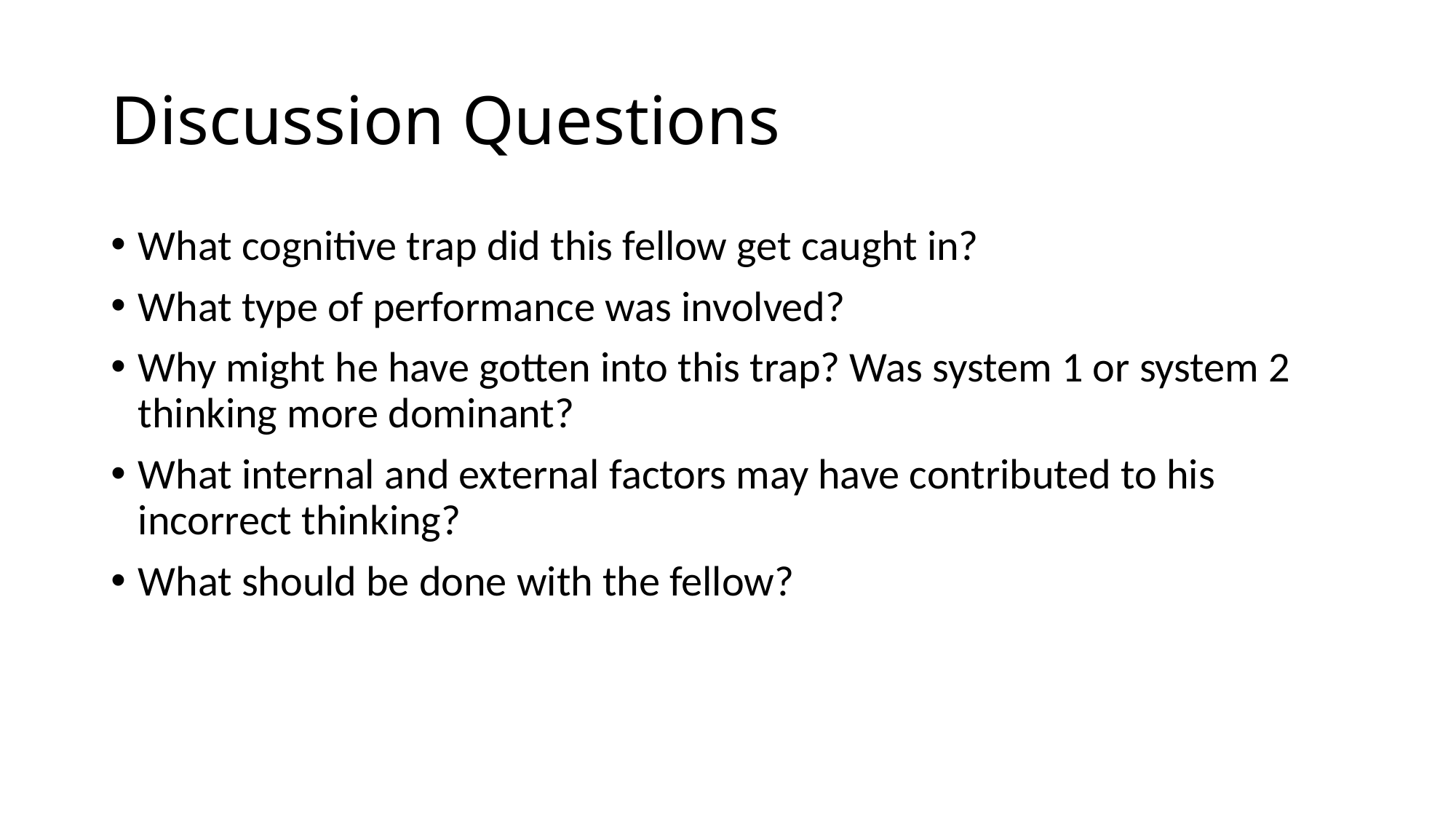

# Discussion Questions
What cognitive trap did this fellow get caught in?
What type of performance was involved?
Why might he have gotten into this trap? Was system 1 or system 2 thinking more dominant?
What internal and external factors may have contributed to his incorrect thinking?
What should be done with the fellow?

## Slide 15
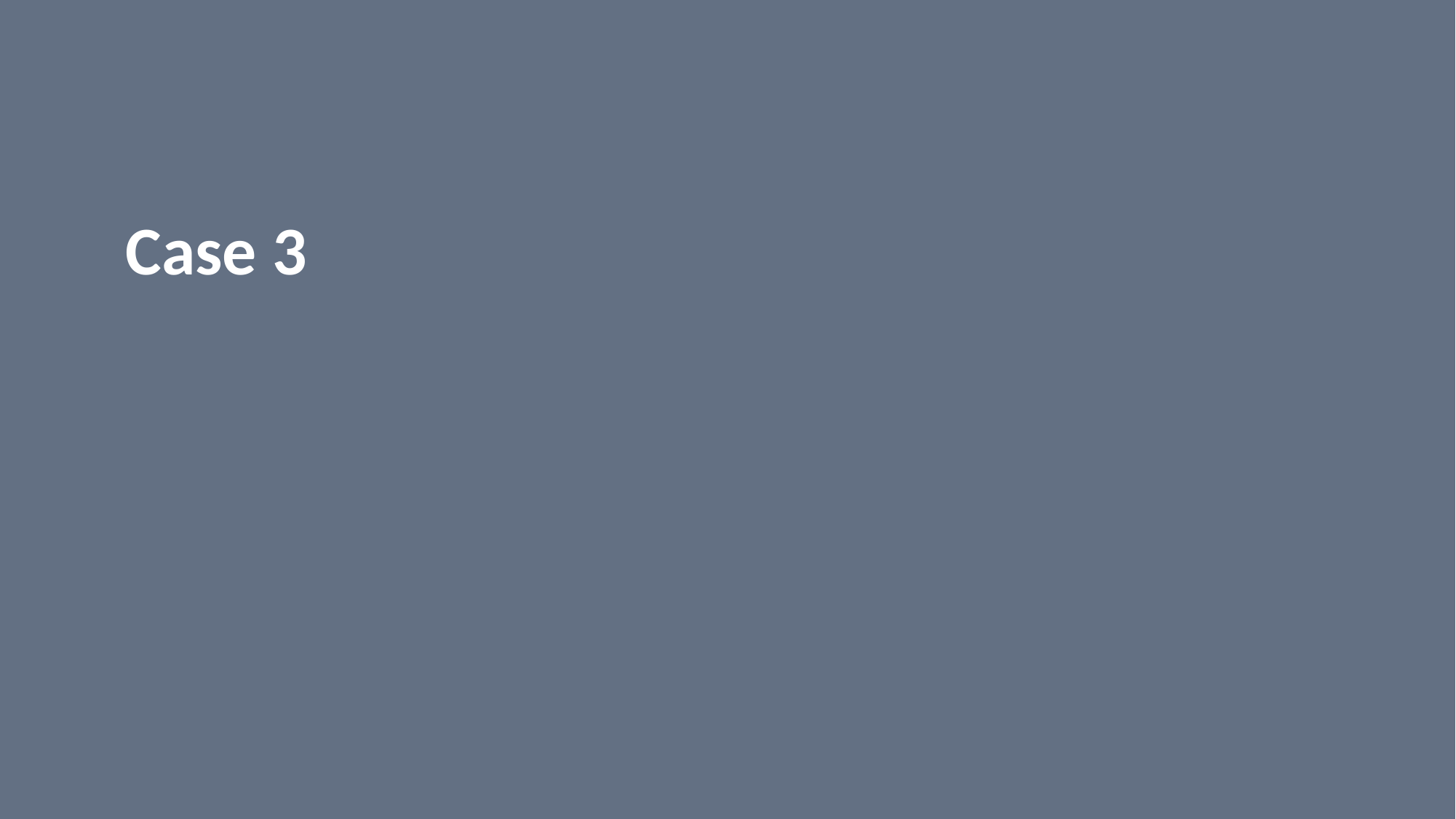

# Case 3

## Slide 16
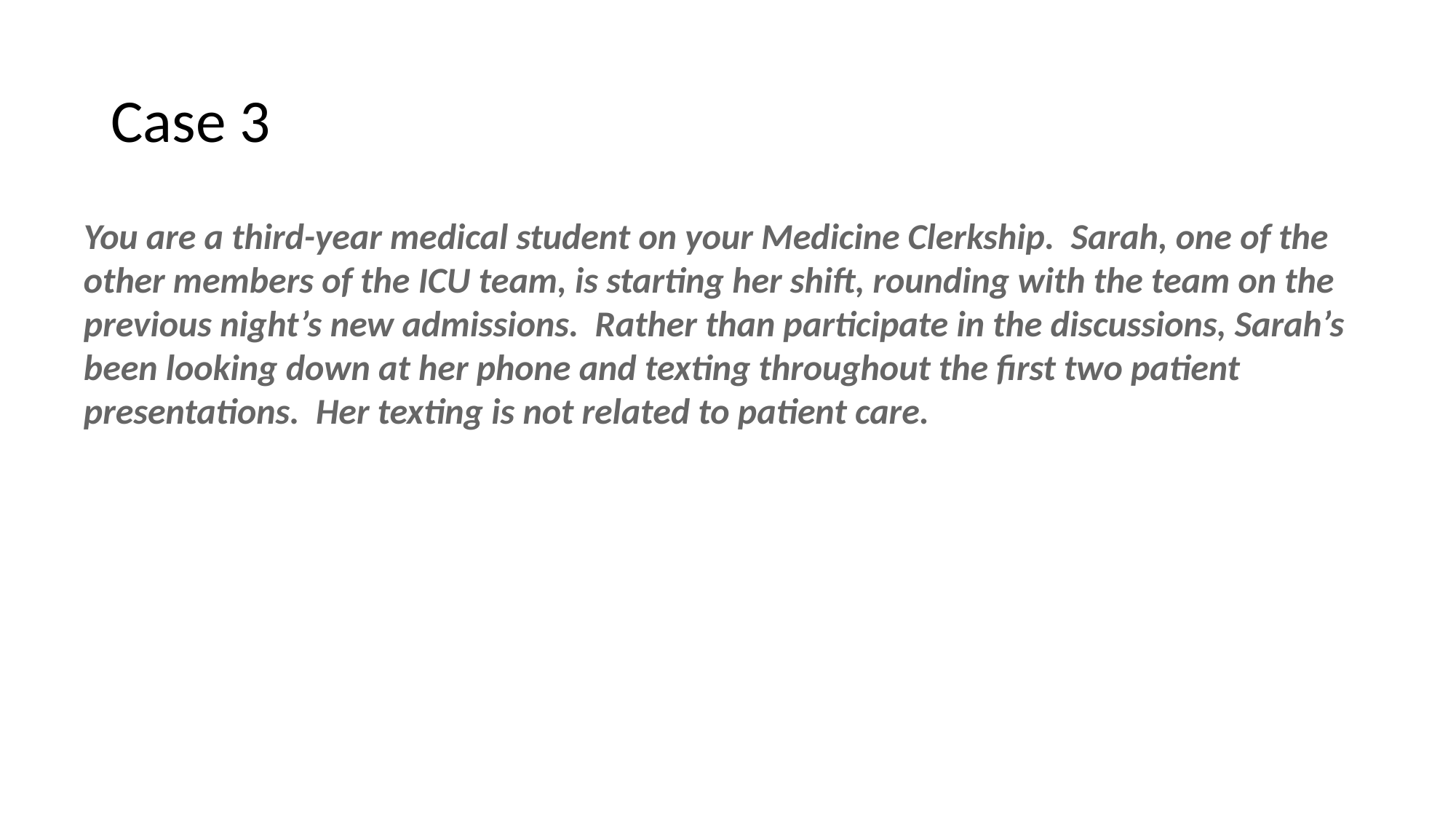

# Case 3
You are a third-year medical student on your Medicine Clerkship. Sarah, one of the other members of the ICU team, is starting her shift, rounding with the team on the previous night’s new admissions. Rather than participate in the discussions, Sarah’s been looking down at her phone and texting throughout the first two patient presentations. Her texting is not related to patient care.

## Slide 17
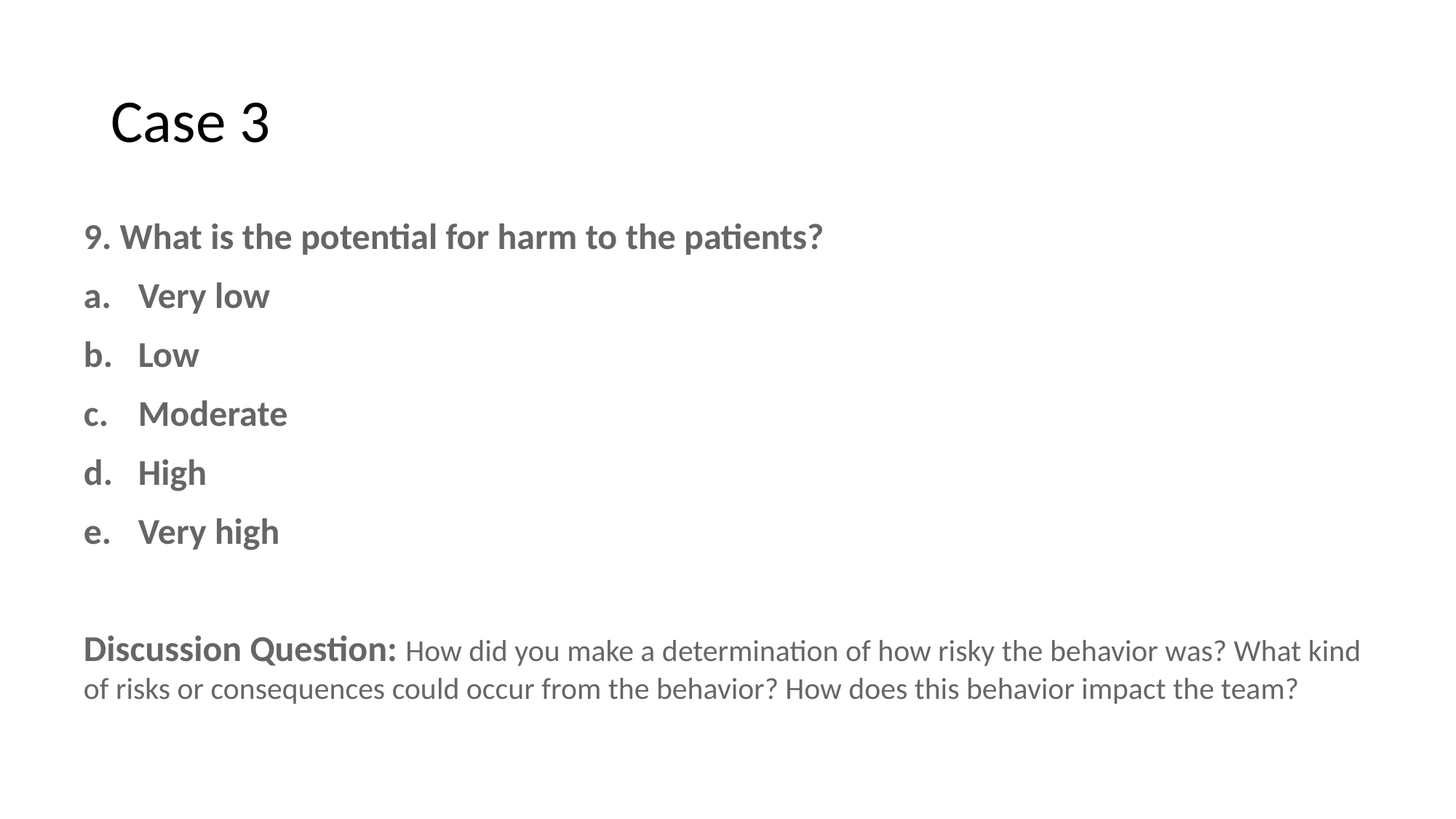

# Case 3
9. What is the potential for harm to the patients?
Very low
Low
Moderate
High
Very high
Discussion Question: How did you make a determination of how risky the behavior was? What kind of risks or consequences could occur from the behavior? How does this behavior impact the team?

## Slide 18
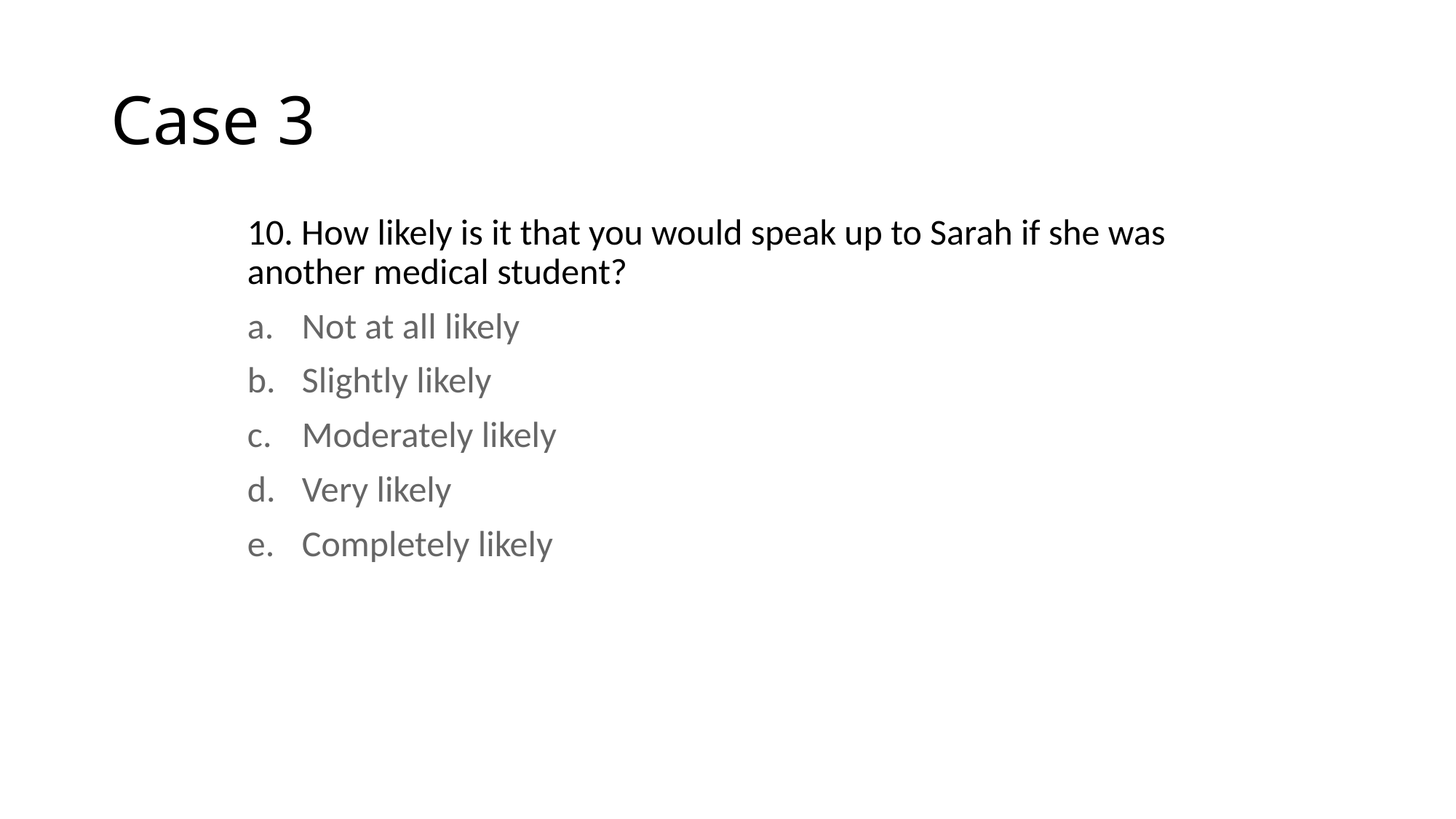

# Case 3
10. How likely is it that you would speak up to Sarah if she was another medical student?
Not at all likely
Slightly likely
Moderately likely
Very likely
Completely likely

## Slide 19
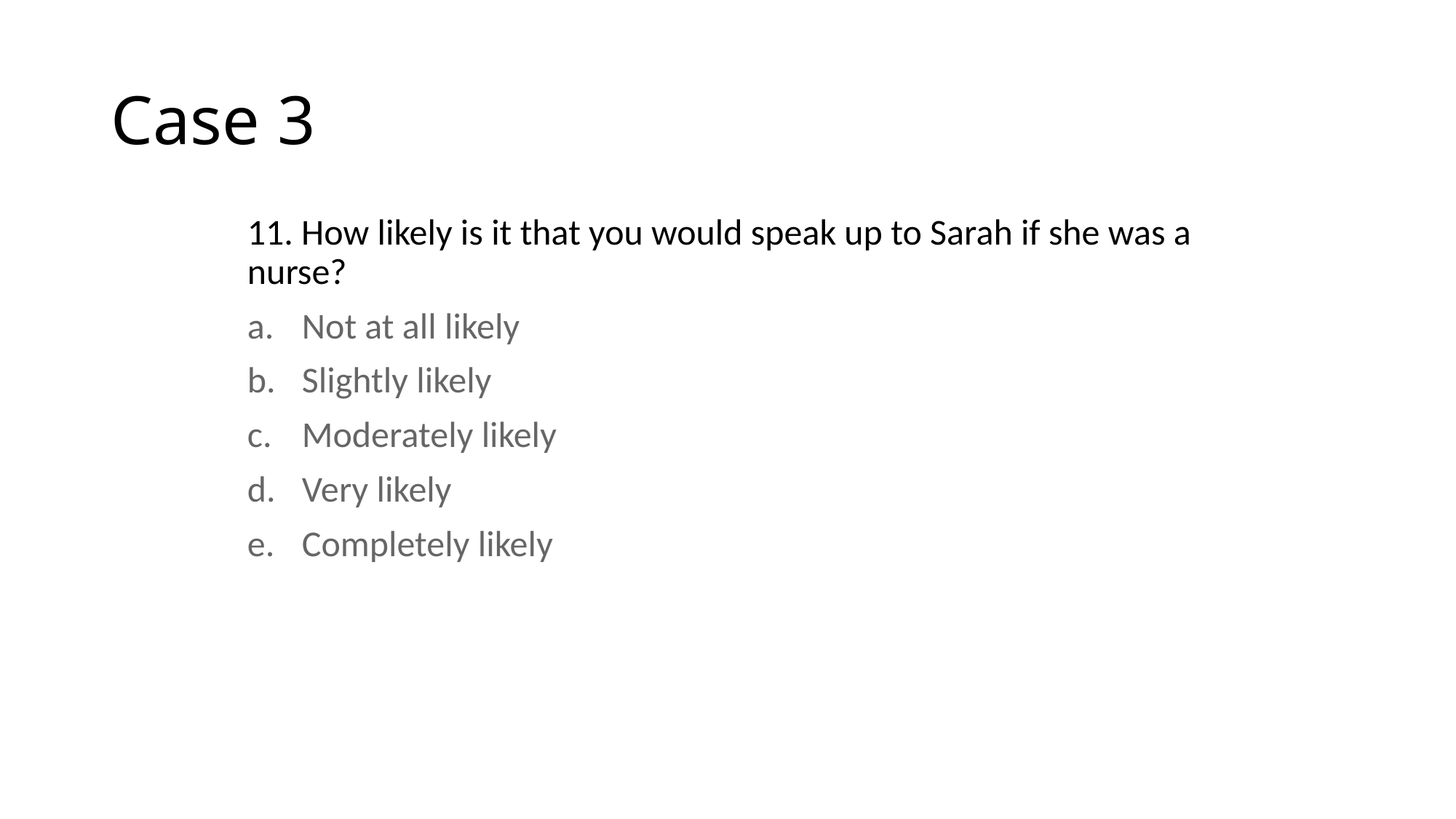

# Case 3
11. How likely is it that you would speak up to Sarah if she was a nurse?
Not at all likely
Slightly likely
Moderately likely
Very likely
Completely likely

## Slide 20
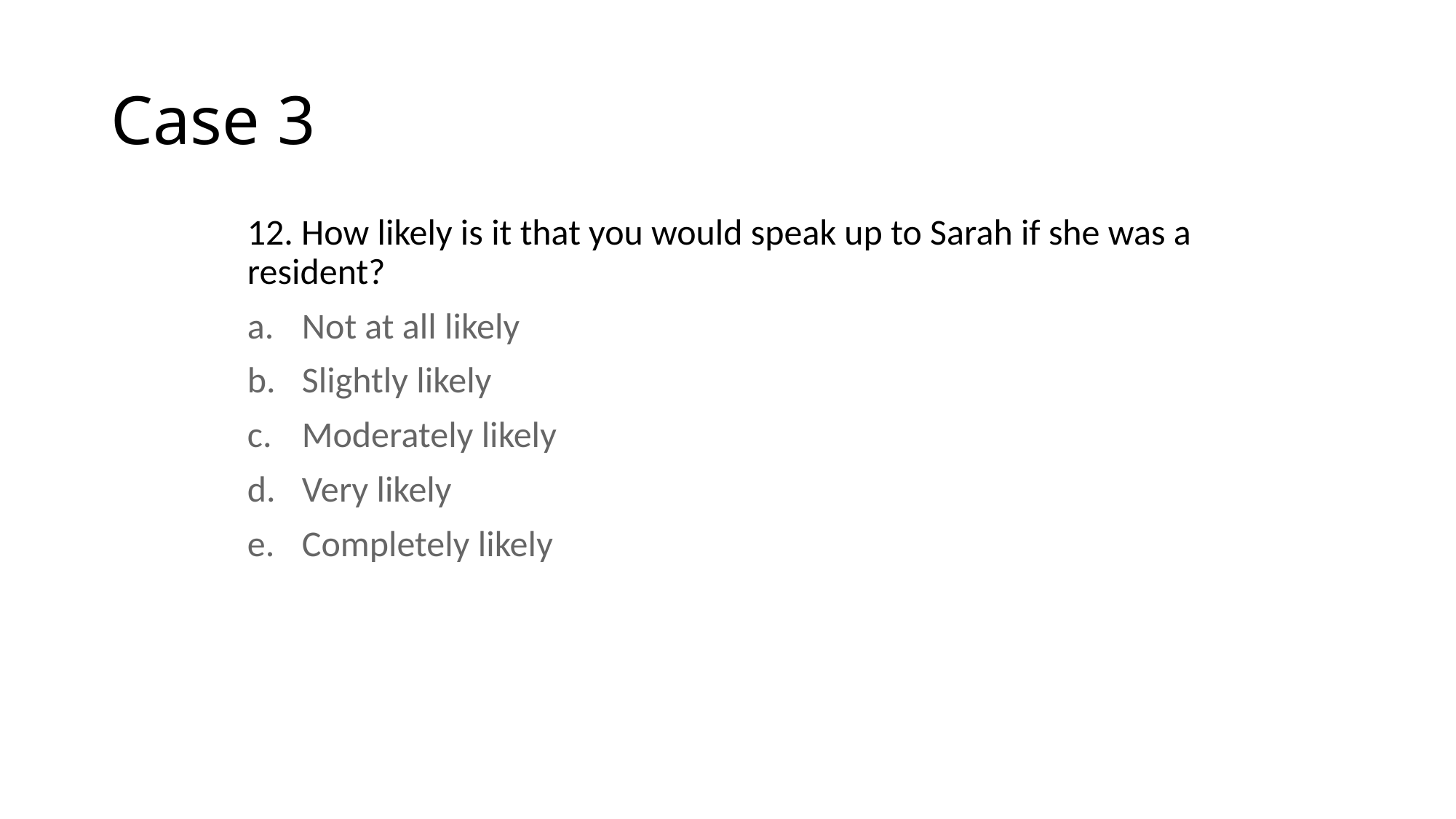

# Case 3
12. How likely is it that you would speak up to Sarah if she was a resident?
Not at all likely
Slightly likely
Moderately likely
Very likely
Completely likely

## Slide 21
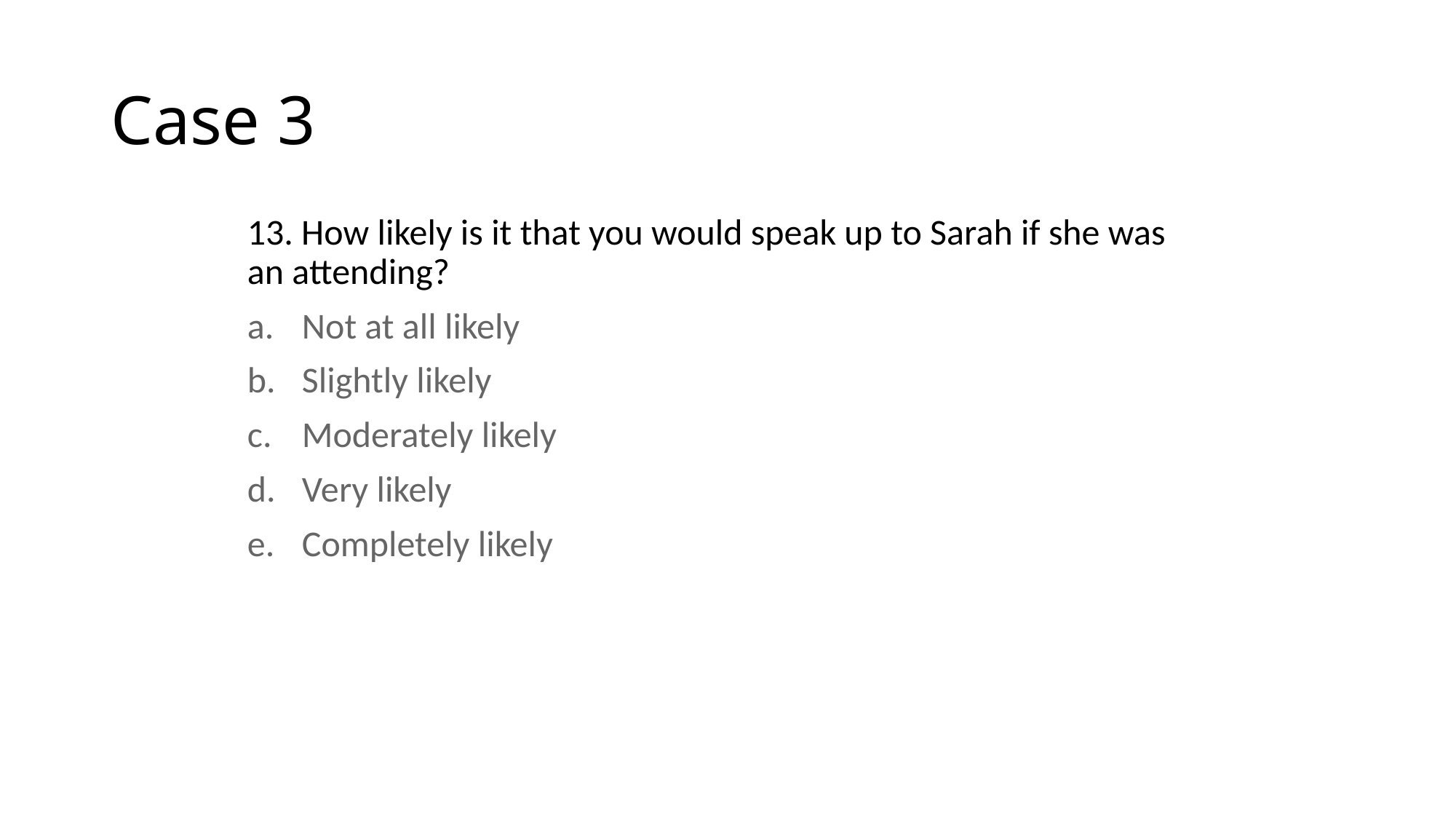

# Case 3
13. How likely is it that you would speak up to Sarah if she was an attending?
Not at all likely
Slightly likely
Moderately likely
Very likely
Completely likely

## Slide 22
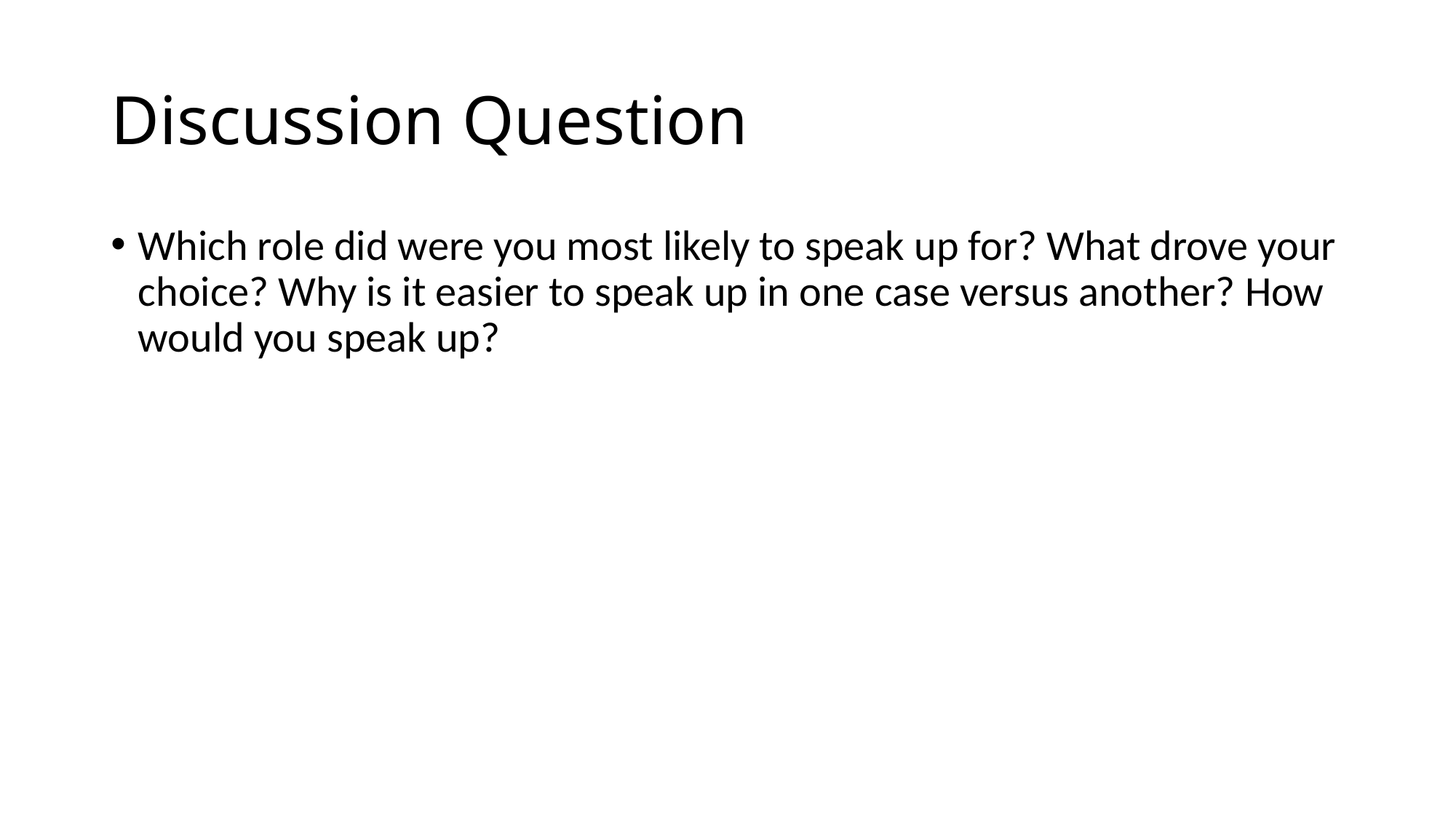

# Discussion Question
Which role did were you most likely to speak up for? What drove your choice? Why is it easier to speak up in one case versus another? How would you speak up?

## Slide 23
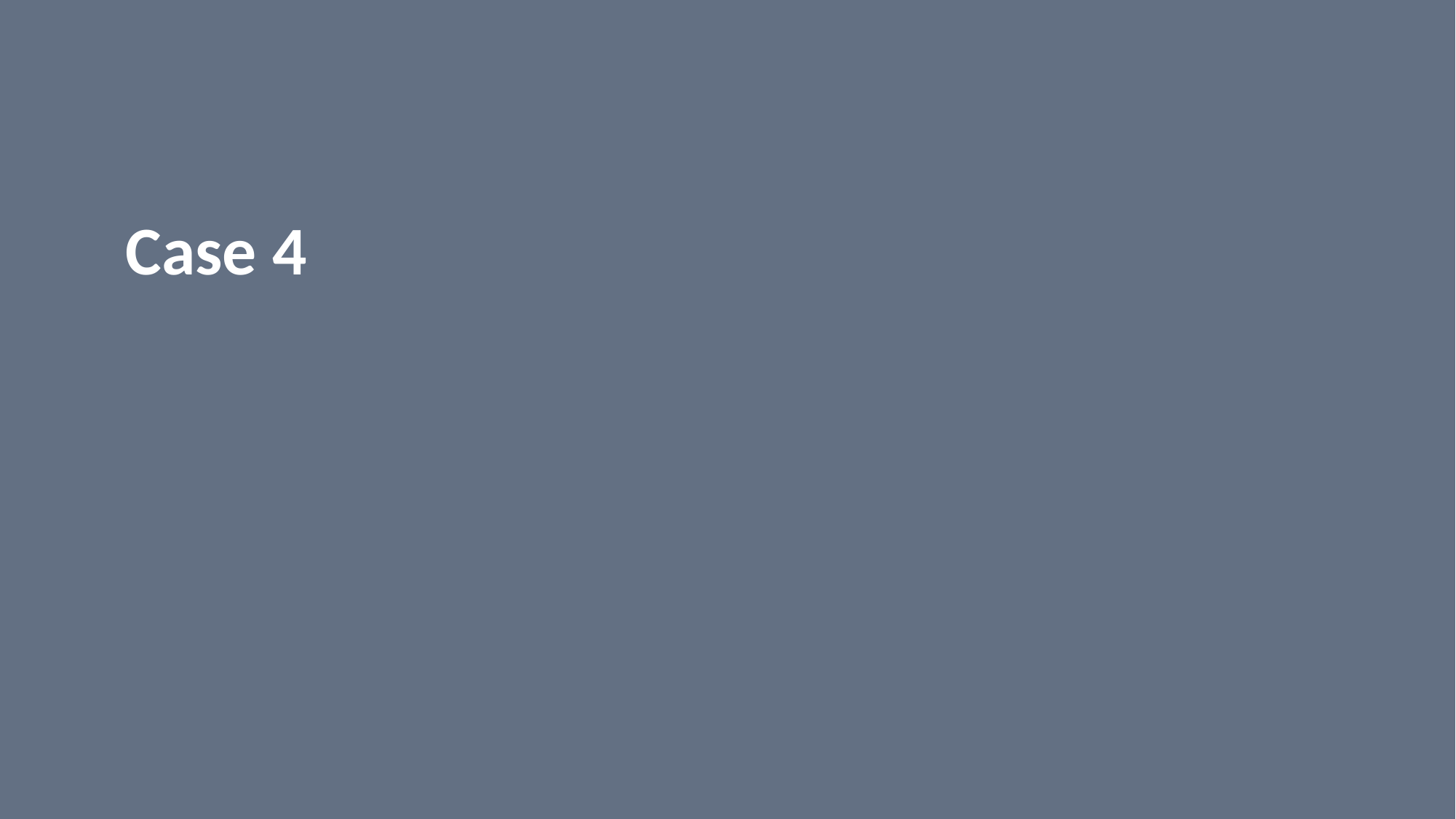

# Case 4

## Slide 24
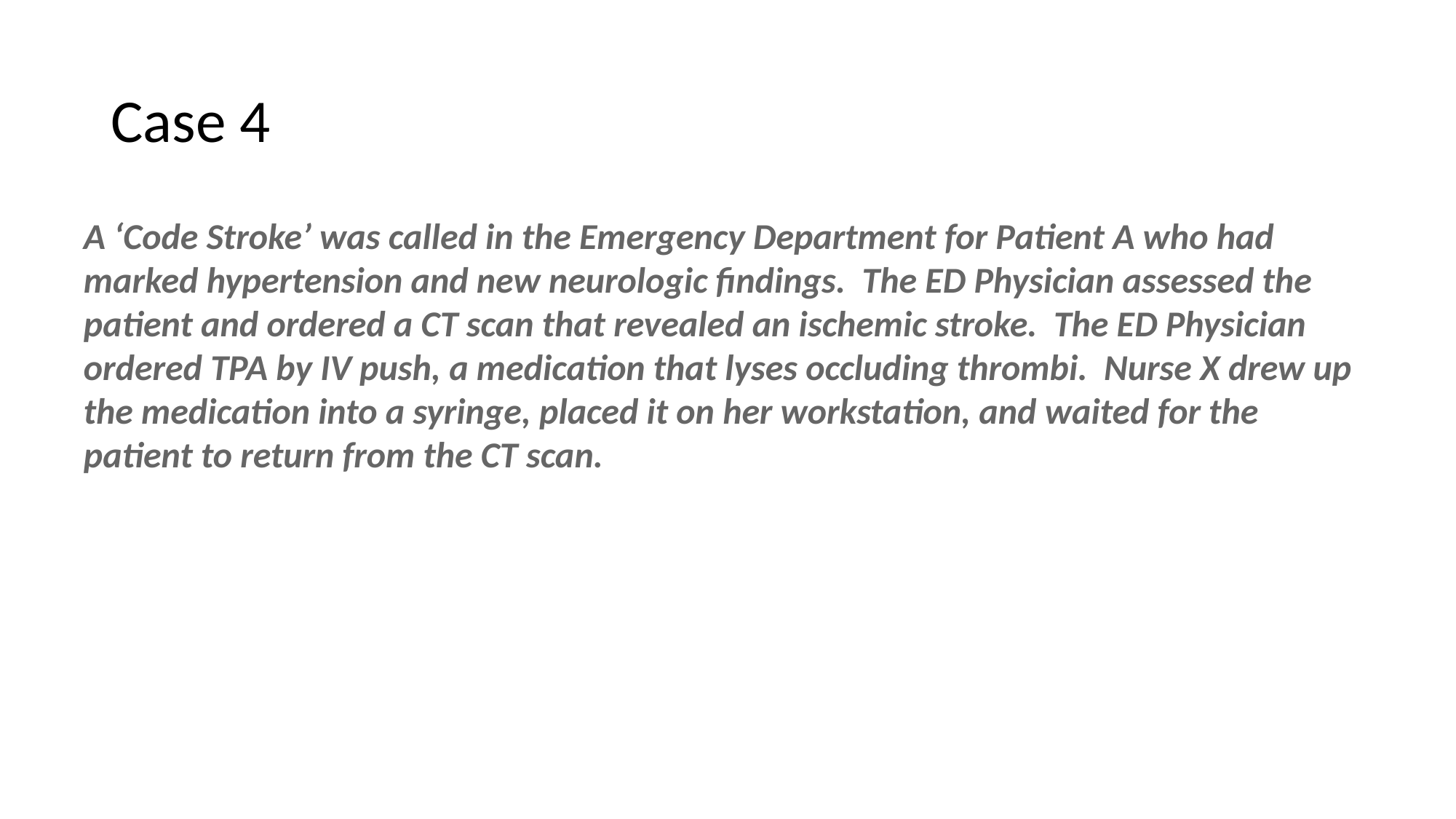

# Case 4
A ‘Code Stroke’ was called in the Emergency Department for Patient A who had marked hypertension and new neurologic findings. The ED Physician assessed the patient and ordered a CT scan that revealed an ischemic stroke. The ED Physician ordered TPA by IV push, a medication that lyses occluding thrombi. Nurse X drew up the medication into a syringe, placed it on her workstation, and waited for the patient to return from the CT scan.

## Slide 25
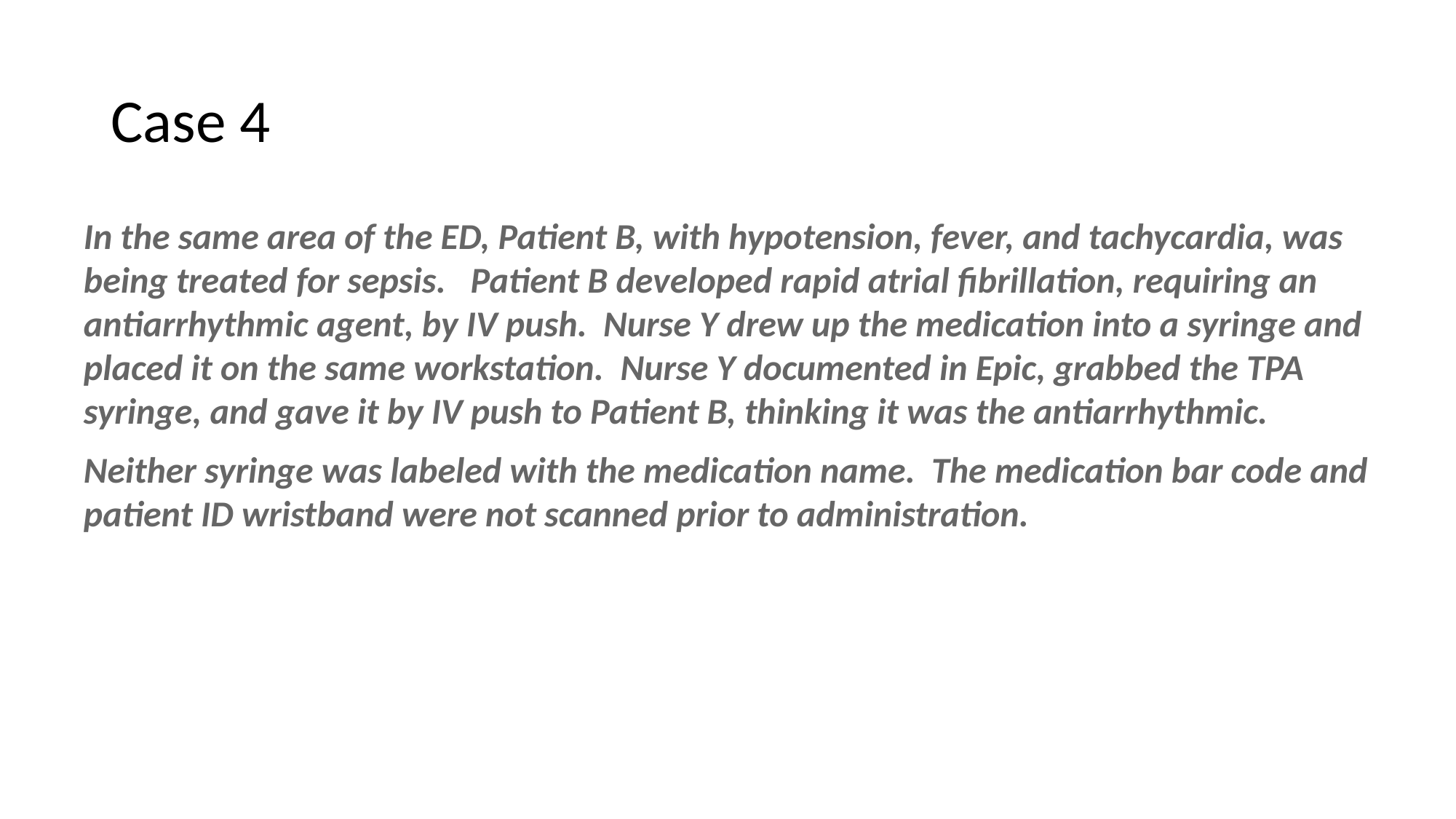

# Case 4
In the same area of the ED, Patient B, with hypotension, fever, and tachycardia, was being treated for sepsis. Patient B developed rapid atrial fibrillation, requiring an antiarrhythmic agent, by IV push. Nurse Y drew up the medication into a syringe and placed it on the same workstation. Nurse Y documented in Epic, grabbed the TPA syringe, and gave it by IV push to Patient B, thinking it was the antiarrhythmic.
Neither syringe was labeled with the medication name. The medication bar code and patient ID wristband were not scanned prior to administration.

## Slide 26
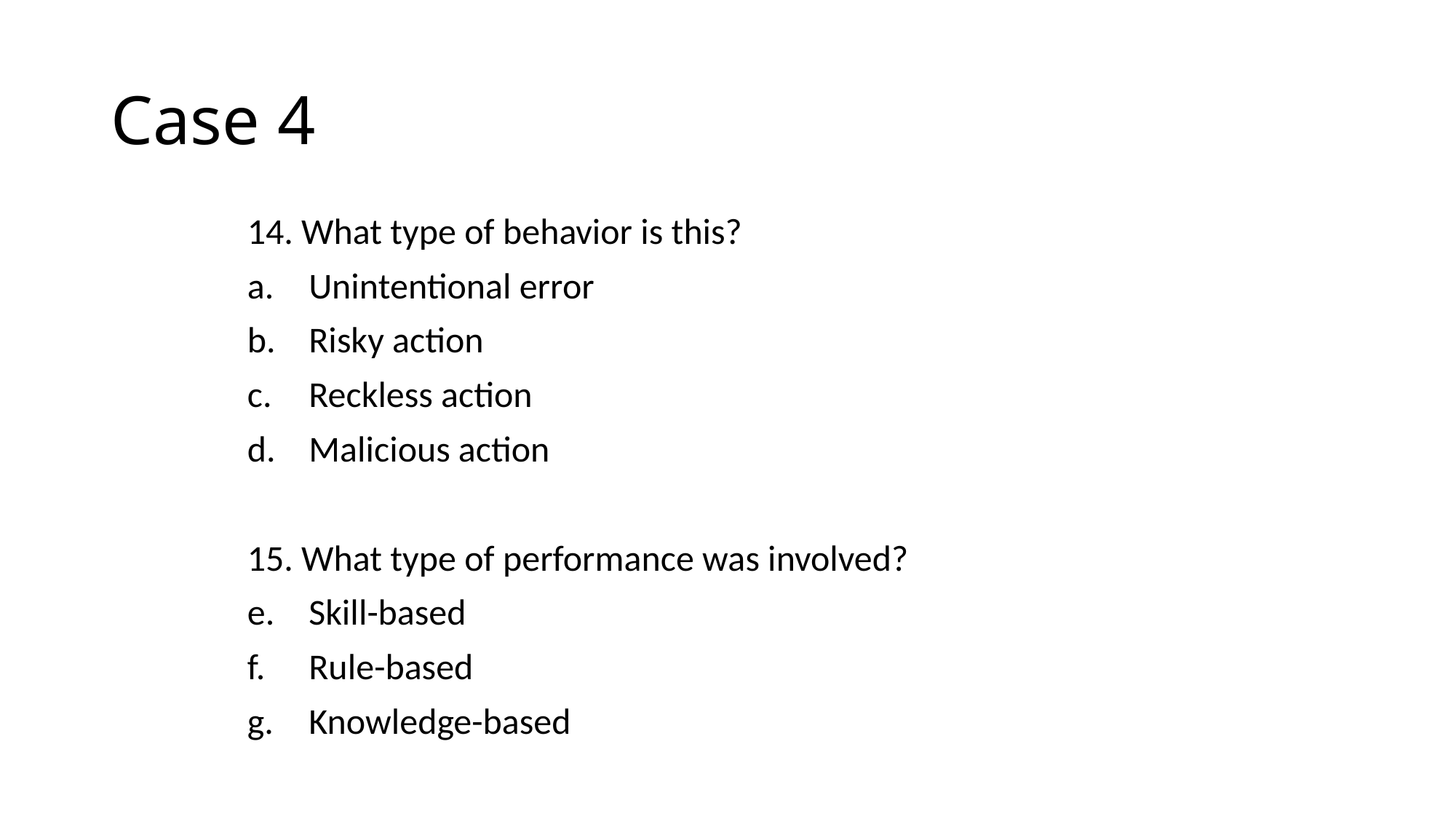

# Case 4
14. What type of behavior is this?
Unintentional error
Risky action
Reckless action
Malicious action
15. What type of performance was involved?
Skill-based
Rule-based
Knowledge-based

## Slide 27
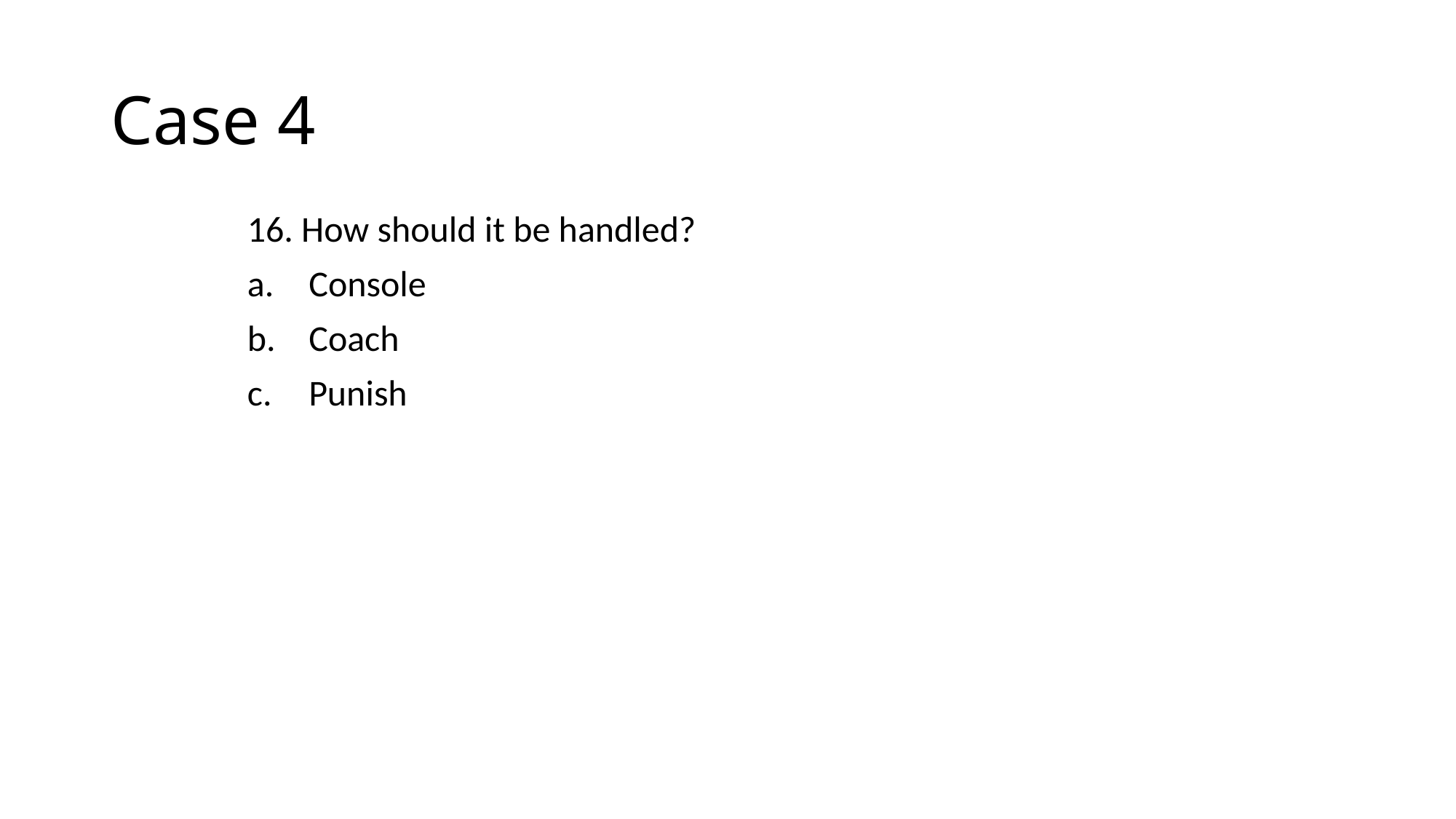

# Case 4
16. How should it be handled?
Console
Coach
Punish

## Slide 28
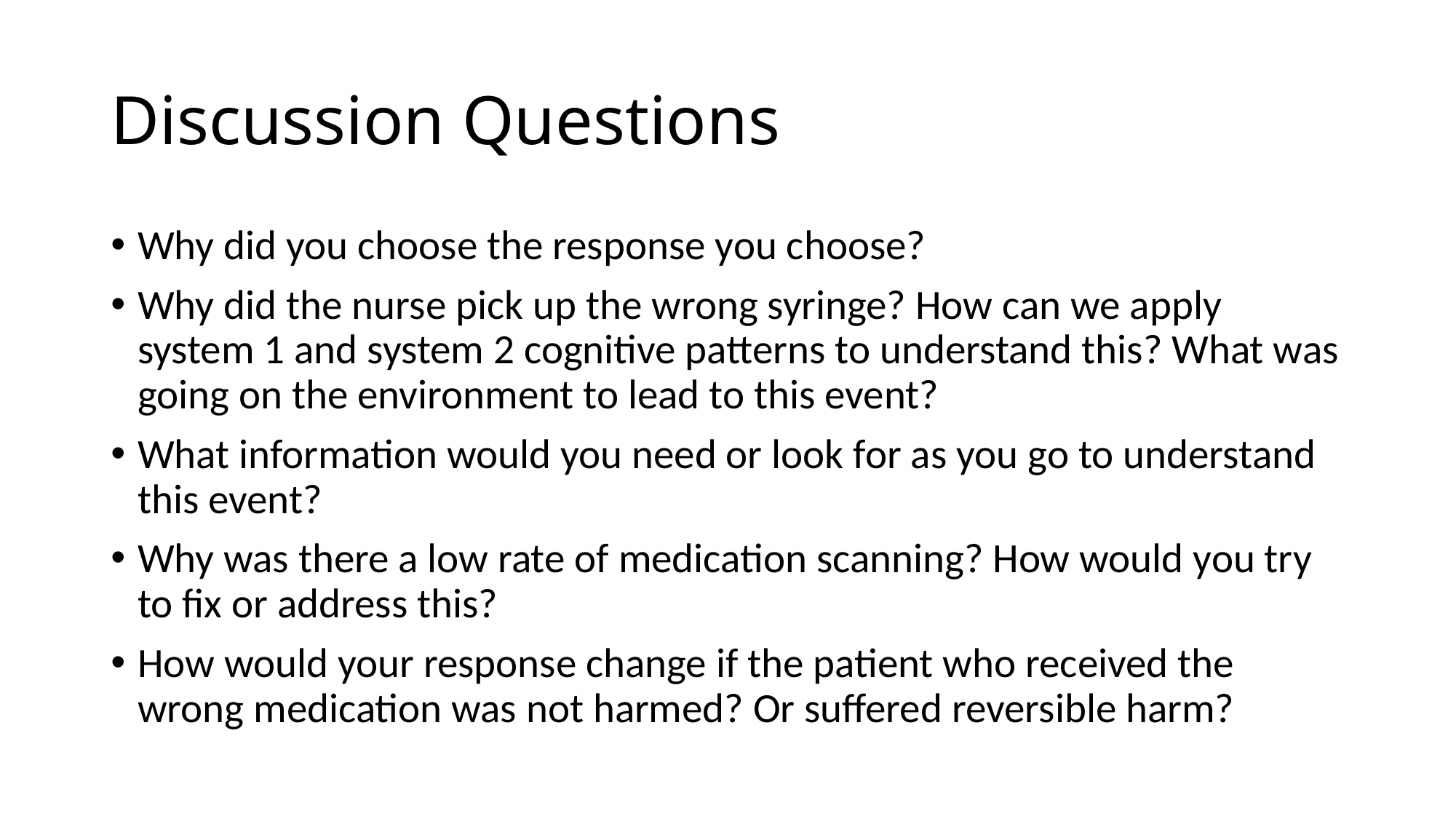

# Discussion Questions
Why did you choose the response you choose?
Why did the nurse pick up the wrong syringe? How can we apply system 1 and system 2 cognitive patterns to understand this? What was going on the environment to lead to this event?
What information would you need or look for as you go to understand this event?
Why was there a low rate of medication scanning? How would you try to fix or address this?
How would your response change if the patient who received the wrong medication was not harmed? Or suffered reversible harm?

## Slide 29
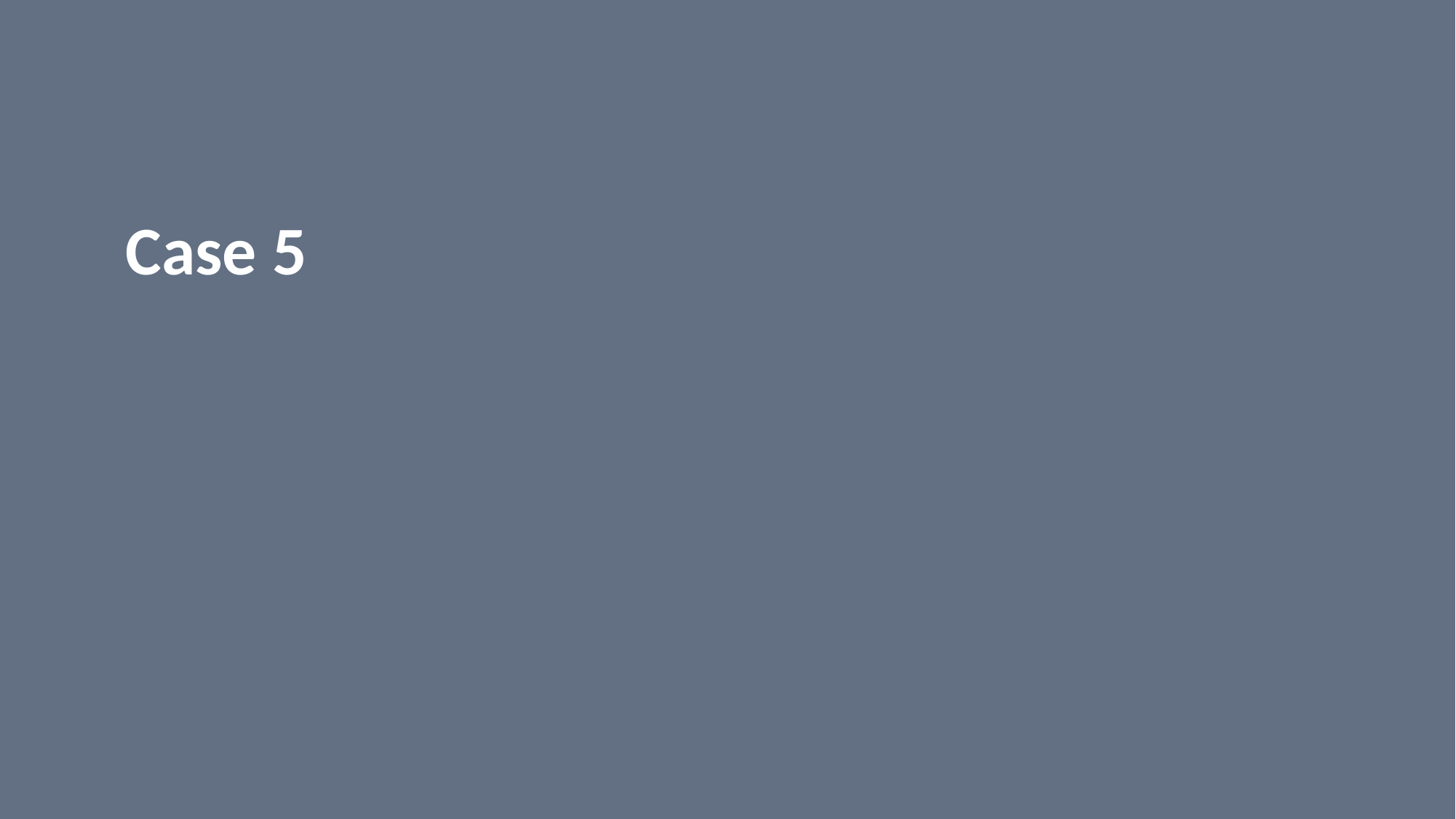

# Case 5

## Slide 30
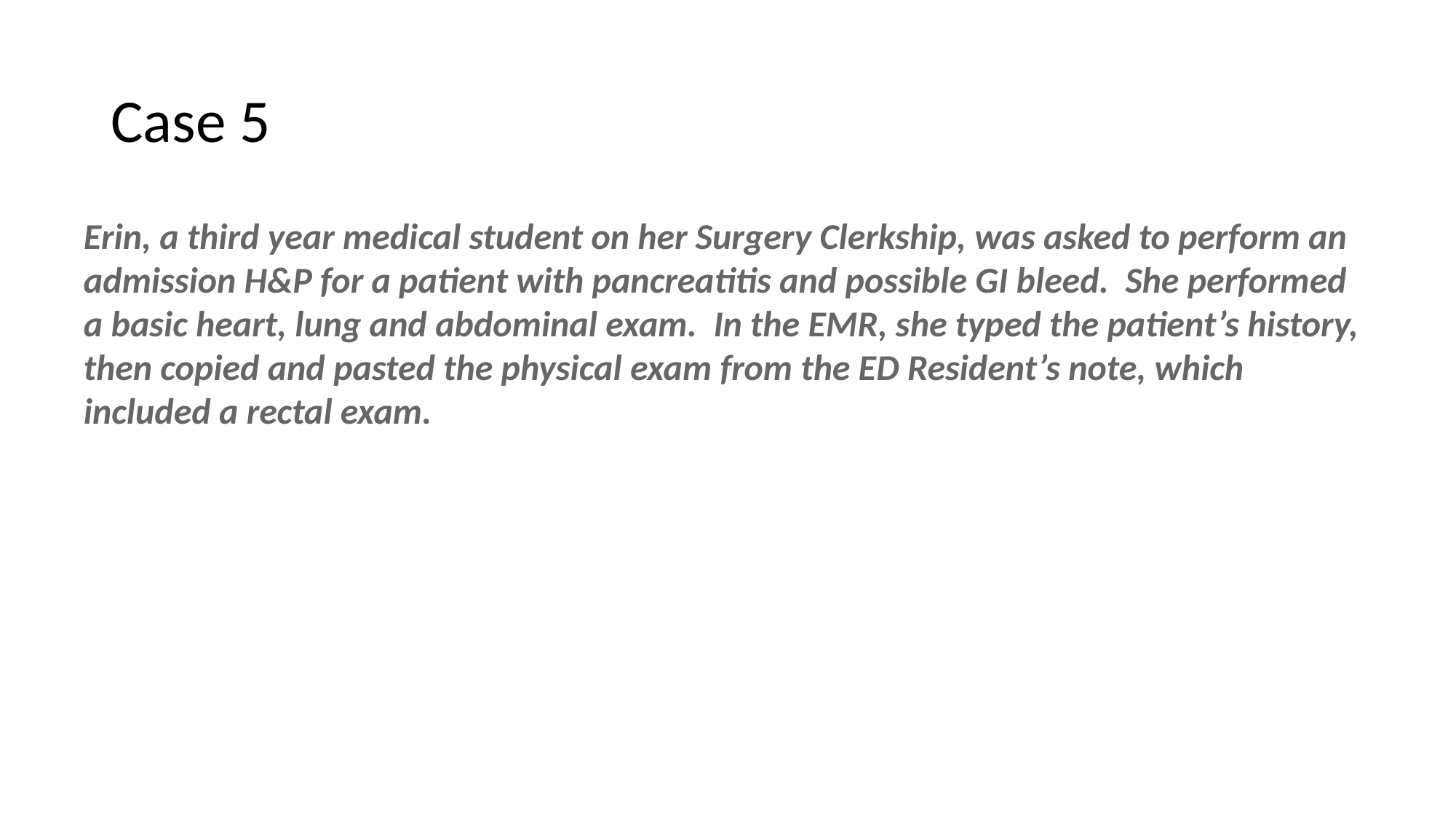

# Case 5
Erin, a third year medical student on her Surgery Clerkship, was asked to perform an admission H&P for a patient with pancreatitis and possible GI bleed. She performed a basic heart, lung and abdominal exam. In the EMR, she typed the patient’s history, then copied and pasted the physical exam from the ED Resident’s note, which included a rectal exam.

## Slide 31
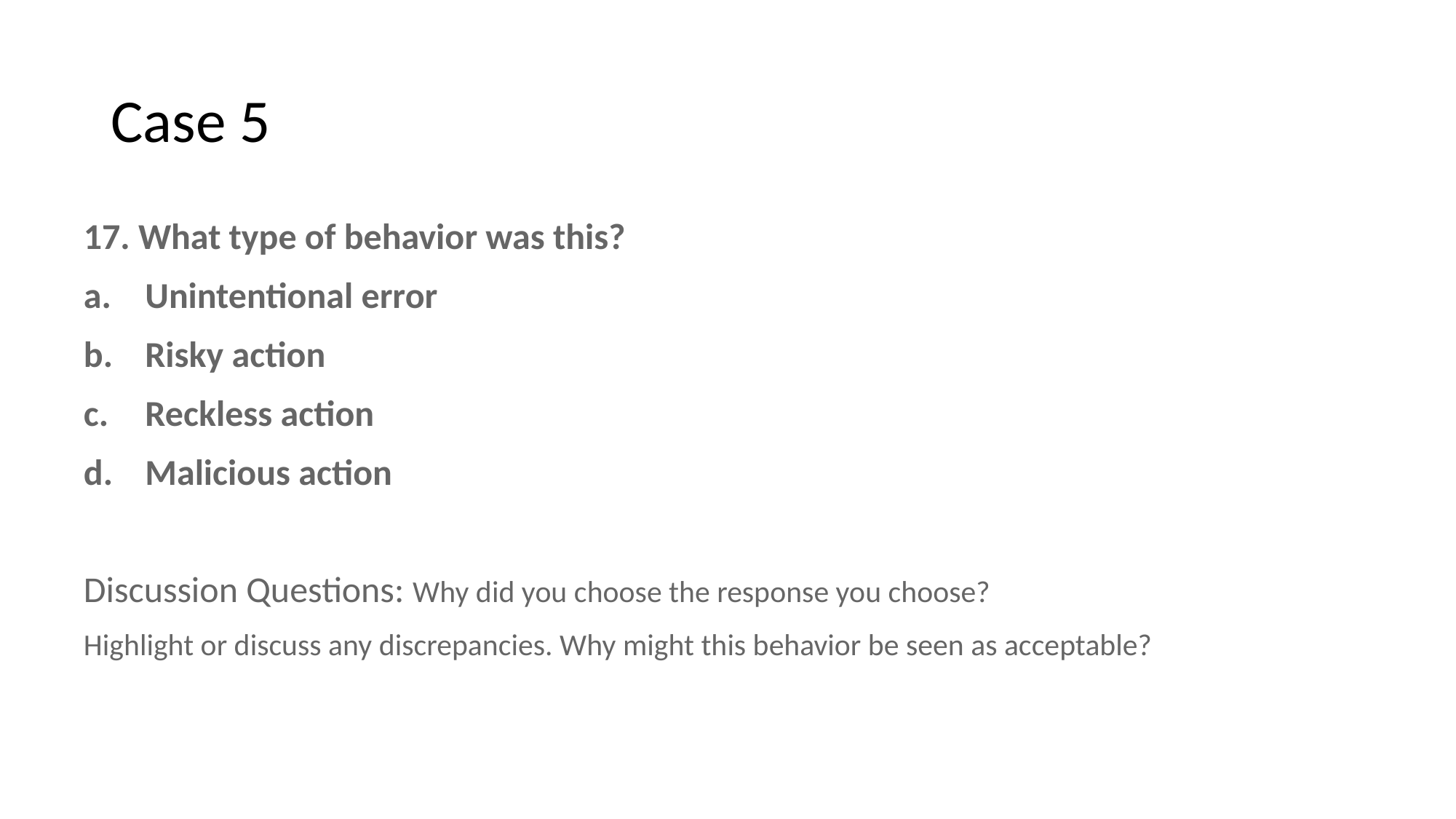

# Case 5
17. What type of behavior was this?
Unintentional error
Risky action
Reckless action
Malicious action
Discussion Questions: Why did you choose the response you choose?
Highlight or discuss any discrepancies. Why might this behavior be seen as acceptable?

## Slide 32
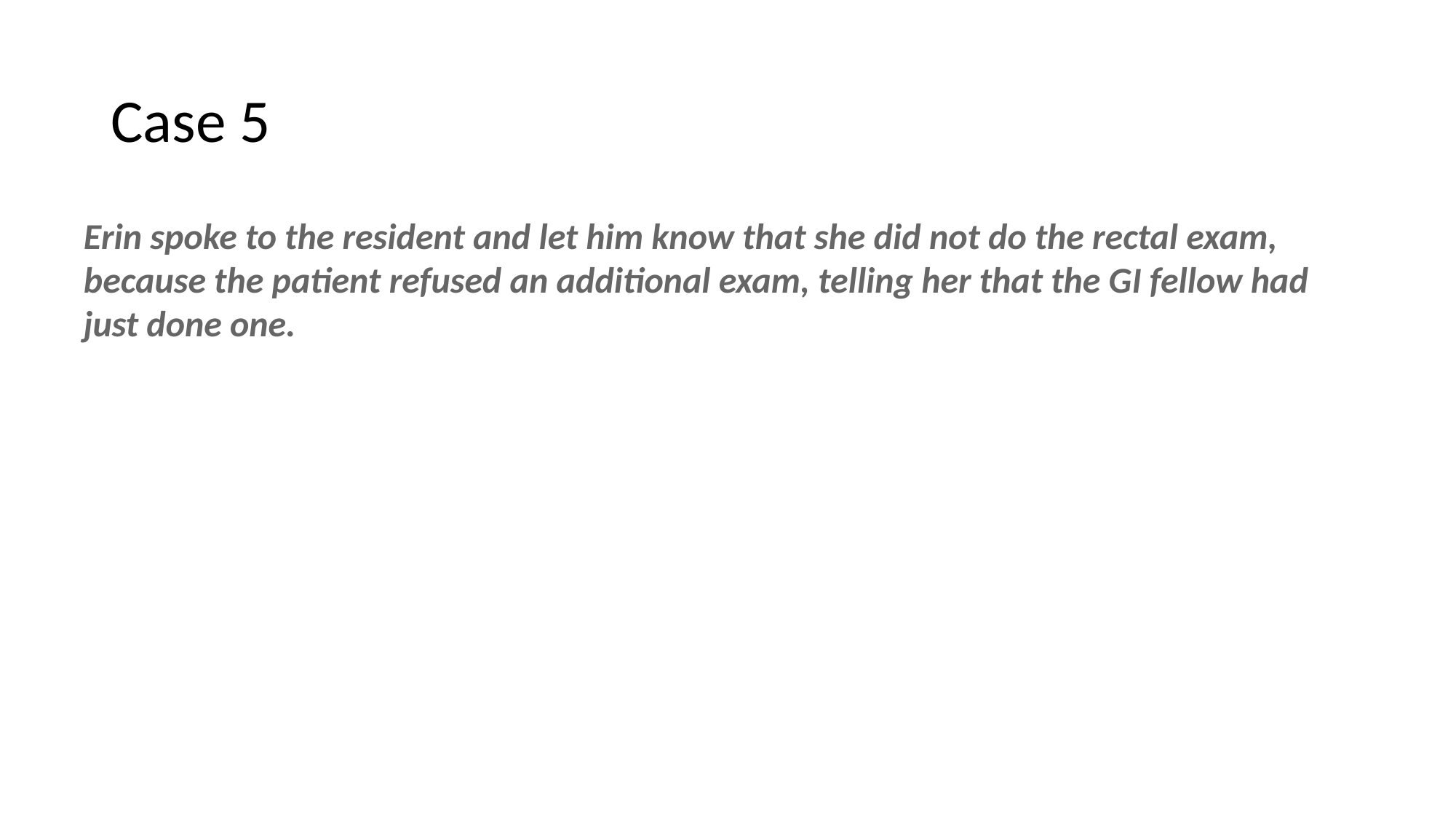

# Case 5
Erin spoke to the resident and let him know that she did not do the rectal exam, because the patient refused an additional exam, telling her that the GI fellow had just done one.

## Slide 33
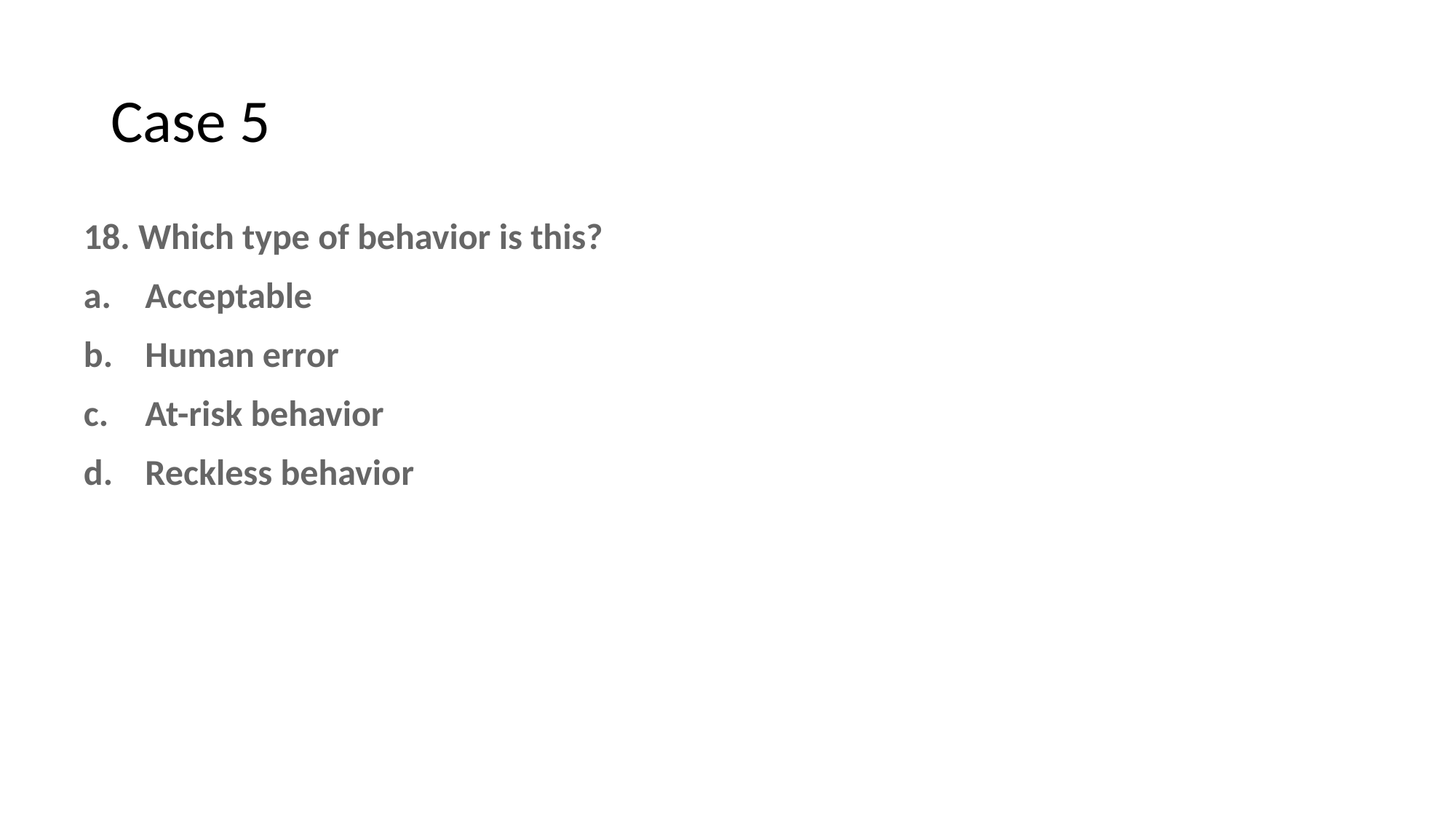

# Case 5
18. Which type of behavior is this?
Acceptable
Human error
At-risk behavior
Reckless behavior

## Slide 34
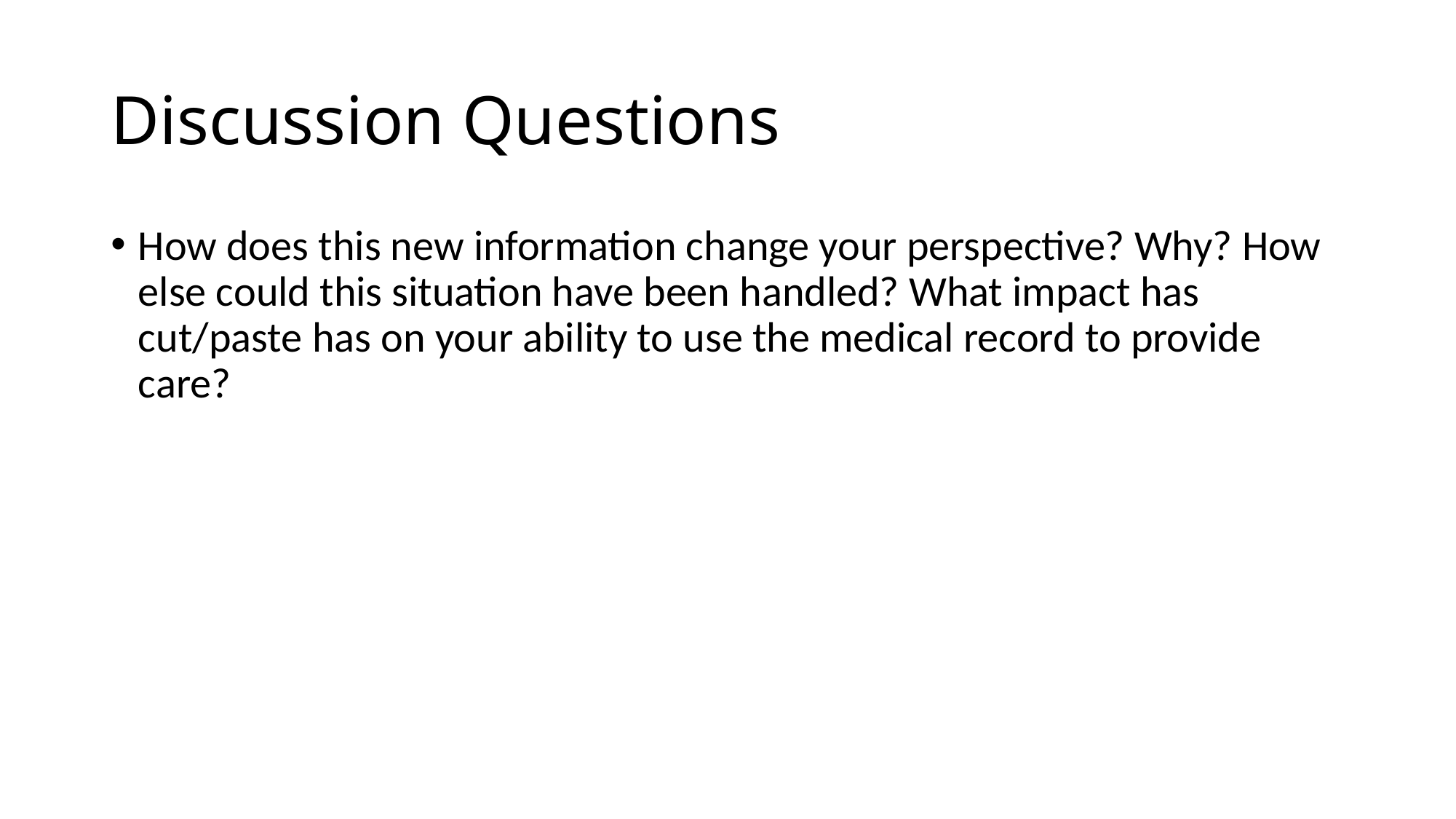

# Discussion Questions
How does this new information change your perspective? Why? How else could this situation have been handled? What impact has cut/paste has on your ability to use the medical record to provide care?
