# Supplementary material for: Just Culture for Medical Students: Understanding Response to Providers in Adverse Events
Source: MedEdPORTAL. 2021 Jul 9;17:11167. doi: 10.15766/mep_2374-8265.11167 (PMC8266940; doi:10.15766/mep_2374-8265.11167)
Supplement: Supplementary file 1 — Slides for Cases.pptxLecture Slides.pptxFaculty Guide.docxQuiz and Evaluation Items.docx [file mep_2374-8265.11167-s001.zip › C. Faculty Guide.docx]

Faculty Guide

Just Culture Small Group Session

**Just Culture Small Group**

Total Time: 60 Minutes

(Time breakdown for each step e.g. Intro, Case, Review, etc)

*Overview for faculty: This session is part of InFocus 6 - a five day, skills-based module for medical students in their 3rd year. This session is designed to teach students specific sets skills that are commonly asked of third year students: (list skills previously numbers). Throughout the session there is an emphasis on patient safety.*

*Students will be in groups of 12-15.*

Faculty: A Patient Safety leader from one of the health system sites who has some Just Culture training.

Equipment Needed:

- Computer, projector

*Overview*

Patient Safety and QI:

- Applying concepts of Just Culture to cases
- HIPPA and Infection Control

Institutional Initiatives that ensures PS and QI:

- Focus on high reliability and just culture
- Managing response to adverse events

Introduction:

Review session objectives –

**By the end of this session students will be able to**:

- Recognize how errors differ in the degree of individual accountability and the responses required to address their undesirable outcomes
- Describe factors that create a culture to speak up

A. Pre-work

Prior to the session students will review 6 scenarios and answer questions about the type of behavior which occurred and how they could consider responding to the event. Results from the survey will be collated into slides and the aggregate class results will be reviewed during this session.

The questions each have the same response: coach, console, or punish.

***All the cases being presented today are all based on real events that have occurred in the Mount Sinai Health System.***

B. Review of cases

**Case 1**:

Part 1:

Sam, a third year medical student on OB, was seen not washing his hands before entering into a patient’s room to examine her. He was trained in year 1 and 2 to wash his hand before and after patient care. He was evaluated on this skill during a standardized patient encounter. An infection control nurse notices and approaches the student. The attending notices this interaction.

*Trigger questions*:

Why did you choose the response you choose?

Highlight or discuss any discrepancies.

What type of performance was involved?

What do you think other students would do?

Part 2:

When Sam’s attending gives him feedback about this after rounds, Sam apologies. He was very nervous about his oral presentation at the bedside on this patient and he forgot.

*Trigger questions*:

Which system was dominant in Sam’s mind?

What else contributes to our low rates of handwashing?

What else could have influenced Sam to engage in reckless or at risk behavior?

**Key points**:

- Initially, this is reckless behavior, as you do not have more information about this. The student learned the skill in the pre-clinical years and has demonstrated he understands the importance.
- This is reckless behavior based on the information provided. However, drift in culture for handwashing may also be a contributor especially in rooms with contact precautions where gloves are being used and many providers so not hand wash before and after gloving. The additional information may place this in the risk action category.
- There is strong evidence that leader/follower behavior exists with hand hygiene. If the first person entering a room performs hand hygiene, the others are more likely to follow suit. This is a critical point as student will become role models soon.
- Obtaining more information behavior passing judgement is key, as the attending did when providing Sam feedback.
- Sam’s system 1 thinking would normally tell him to wash hands however his mental effort was diverted by the oral presentation, the system 2 task, which was at hand.
- Role modeling behavior can influence how junior members behave. Power and hierarchy also play a role.
- Our system has a low rate of hand washing amongst physicians suggesting that others would do this. What system solutions could the team propose?

**Case 2:**

A GI fellow is performing paracentesis for a 59 year old M with DM, HTN (on three medications) and obesity who comes in for a diagnostic paracentesis. This is the patient’s first paracentesis and has no documented portal hypertension. The fellow has performed over 50 of these procedures this year and is appropriate for indirect supervision. He performs the procedure without difficulty. Post procedure the patient’s blood pressure 90 mins after the procedure is 80/55. The patient leaves, collapses in the elevator and arrests. A post mortem exam reveals a cardiac abscess and peritoneal carcinomatosis and no liver disease.

During the debrief, the fellow mentions that he did not considering evaluating the hypotension because most patients with liver disease have a low blood pressure.

*Trigger question*:

What cognitive trap did this fellow get caught in?

What type of performance was involved?

Why might he have gotten into this trap? Was system 1 or system 2 thinking more dominant?

What internal and external factors may have contributed to his incorrect thinking?

*Trigger question*:

What should be done with the fellow? Review responses from the pre-survey and discuss.

**Key Points**:

- This fellow fell into a trap of availability bias since most patients he does this procedure on normally have low blood pressures. In fact, this patient has a high blood pressure at baseline as seen by the fact he takes 3 outpatient medications for hypertension.
- Production pressure, false sense of security having done many of these procedures could also play into the approach.
- Based on the available information, the fellow should be coached about how to assess and manage hypotension in a patient post paracentesis.
- In this case the patient was ultimately septic from a cardiac abscess which is rare however the low BP evaluation might have prompted concern for infection. The fellow should also be offered support as he may also feel like the 2^nd^ victim .

**Case 3:**

You are a third-year medical student on your Medicine Clerkship. Sarah, one of the other members of the ICU team, is starting her shift, rounding with the team on the previous night’s new admissions. Rather than participating in the discussions, she’s been looking down at her phone switching between Facebook and texting throughout the first two patient presentations. Her texting is not related to patient care.

*Trigger questions for risk rating item*: How did you make a determination of how risky the behavior was? What kind of risks or consequences could occur from the behavior? How does this behavior impact the team?

*Trigger questions for speaking up based on role*:

Which role did were you most likely to speak up for? What drove your choice? Why is it easier to speak up in one case versus another? How would you speak up?

**Key points:**

The purpose of this scenario is two fold- **to have students determine how distractions during rounding can be risky and why.** Texting is a common behavior and one which is seen during rounds. However, it does have safety and team work implications.

The second purpose of this scenario is to understand **what drives speaking up behavior**. One challenge in speaking up for team members, especially medical students, is the power dynamic which exists on the team. Students are in the most vulnerable position to speak up however may have the freshest set of eyes and ears to advocate for safety as they are not enmeshed in the professional culture of the profession or service line.

Data from a survey of nearly 700 residents at Vanderbilt, showed that residents were most likely to speak up if the team member was a nurse or intern and less likely if it was a resident. Less than 10% of resident respondent would speak up if the team member was an attending (Martinez W, BMJ 2017).

As you facilitate this section have the students discuss three sets of factors the drive speaking up, which are summarized in this table:

| Personal | Relational | Contextual |
| --- | --- | --- |
| Knowledge base  Sense of personal responsibility for the patient  Autonomy  Interpersonal skills  Empowerment to speak up  Assertiveness  Moral courage  Desire to be “liked”  Psychological safety  Job satisfaction  Causal attribution | Relationship between two individuals—approachability  Privacy  Encouragement from superiors to report  Prior experiences with speaking up  Fear of alienation from a group/team  Fear of retribution/bad evaluation | Nature of the safety threat  Potential of harm  Frequency of rule violation  Power  Hierarchy  Safety culture  Will meaningful change happen after speaking up?  Training in how to speak up  Knowing where to get help  How does the organization recognize people who speak up?  Confidentiality |

Adapted from Martinez, William, et al. "Speaking up about traditional and professionalism-related patient safety threats: a national survey of interns and residents." BMJ Quality & Safety 26.11 (2017): 869-880.

Discussion questions:

Say: *“We can think of three types of factors that make it easier or harder to speak up for safety. These can be our own personal factors, the nature of the relationship between us and the person who is being unsafe, and the team or clinical environment.”*

1. What are reasons that relate to you or reside within us that make it easier or harder to speak up?
2. What factors in our relationship with other team members might impact speaking up? Why is it easier with to speak up if it is the Sub-intern/acting intern versus the attending?
3. How does the clinical environment impact this? Why might it be easier to speak up in the clinic versus the operating room?

As students list the reasons, consider creating a list on the board or having someone scribe. Then show the summary slide.

**Case 4:**

A ‘Code Stroke’ was called in the Emergency Department for Patient A who had marked hypertension and new neurologic findings. The ED Physician assessed the patient and ordered a CT scan that revealed an ischemic stroke. The ED Physician ordered TPA by IV push, a medication that lyses occluding thrombi. Nurse X drew up the medication into a syringe, placed it on her workstation, and waited for the patient to return from the CT scan.

In the same area of the ED, Patient B, with hypotension, fever, and tachycardia, was being treated for sepsis. Patient B developed rapid atrial fibrillation, requiring an antiarrhythmic agent, by IV push. Nurse Y drew up the medication into a syringe and placed it on the same workstation. Nurse Y documented in Epic, grabbed the TPA syringe, and gave it by IV push to Patient B, thinking it was the antiarrhythmic.

Neither syringe was labeled with the medication name.

The medication bar code and patient ID wristband were not scanned prior to administration.

*Trigger questions*:

Why did you choose the response you choose?

Highlight or discuss any discrepancies.

Why did the nurse pick up the wrong syringe? How can we apply system 1 and system 2 cognitive patterns to understand this? What was going on the environment to lead to this event?

What information would you need or look for as you go to understand this event?

Why was there a low rate of medication scanning? How would you try to fix or address this?

How would your response change if the patient who received the wrong medication was not harmed? Or suffered reversible harm?

**Key Points**:

- This case was based on a real event.
- There are two human elements to the event in this case—a human error in selecting the wrong syringe and an at risk behavior not using bar code/scanning technology or the process of verification when administering the medication. The discussion should focus on why these safety procedures were not done.
- Explore whether students choose to console, coach or punish the nurse and why before revealing the information discovered in the investigation.
- In the investigation it was discovered there was low rates of scanning to verify patient and medication in the emergency room and that in emergency situations, which were poorly defined, this was routine practice. This finding is an example of **drift**. Other cases in this session show examples of drift (handwashing).
- Individual and service line level coaching along with weekly feedback on scanning compliance were used to address this event.
- The nurse was consoled and offered support through a program called iCare. Briefly explain iCare to the students which is available to them. They can also use their clerkship directors and school leadership if they need support.

**Case 5**:

Part 1:

Erin, a third year medical student on surgery, was asked to write an admission H&P for a patient with pancreatitis and possible GI bleed. She performed a basic heart, lung and abdominal exam. She copy and pasted the physical exam from the ER resident note, which included a rectal exam.

*Trigger questions*:

Why did you choose the response you choose?

Highlight or discuss any discrepancies. Why might this behavior be seen as acceptable?

Part 2:

Erin speaks to the resident and lets him know she did not do the rectal exam because the patient stated the GI fellow just did the exam. The patient refused an additional exam.

*Trigger questions*: How does this new information change your perspective? Why? How else could this situation have been handled? What impact has cut/paste has on your ability to use the medical record to provide care?

**Kay Points:**

- Cut and paste without attribution to the author or source is not professional and dishonest.
- In the first part of the case, the student was reckless. However, the reasoning giving in the second part makes sense but the student should be coached about how to do this.
- With an electronic medical record, the use of cut and paste is pervasive. There is a culturally attitude towards accepting this behavior without always realizing the consequences.
- When investigative adverse events, the chart often has similar appearing notes without new or changed information as the course has changed making it difficult to understand how a patient’s course could be altered.

C. Ideas for Change

“We all struggle with how to make the healthcare environment more open to be safer and to live up to the ideals of a just culture. **You also heard about the 5 characteristics which make a high reliability organization**. Those characteristics are:

Sensitivity to operations

Do not accept simple explanations for events

Preoccupation with failure

Defer to expertise regardless of hierarchy/seniority

Resilient

Think back to your last rotation. Work in groups of 3-4 to come up with 1-2 recommendations your would make to the last rotation you were on to make the environment more Just or to help them take a step toward high reliability.”

**A volunteer should list three recommendations from the group to place on the board. Someone from the group should submit these via Blackboard at the end of the session.**

D. Wrap-up/transition (<1 minute)

Say: ”This session helped you to apply the principles of Just Culture to understanding a response to an adverse event. We reviewed types of human error and to determine how organizations respond to the health care professionals involved. As a vital team member and team leader, you have a role in trying to uphold a Just Culture in the places where you work.”
