# Supplementary material for: Just Culture for Medical Students: Understanding Response to Providers in Adverse Events
Source: MedEdPORTAL. 2021 Jul 9;17:11167. doi: 10.15766/mep_2374-8265.11167 (PMC8266940; doi:10.15766/mep_2374-8265.11167)
Supplement: Supplementary file 1 — Slides for Cases.pptxLecture Slides.pptxFaculty Guide.docxQuiz and Evaluation Items.docx [file mep_2374-8265.11167-s001.zip › D. Quiz and Evaluation Items.docx]

**Post-Test Items**

1. **What is the definition of a just culture?**
2. A culture where individuals are held accountable for all medical errors.
3. A culture where individuals are held accountable for both their own errors and the errors of the system.
4. A culture where individuals feel comfortable disclosing errors but are not professionally accountable.
5. A culture where individuals and systems recognize that professionals make mistakes and empower their frontline providers to disclose errors.

2. **In a just culture, what is the appropriate action to be taken for a human error?**

a. Console

b. Coach

c. Punish

d. Do nothing

3. **In a just culture, what is the appropriate action to be taken for an at risk behavior?**

a. Console

b. Coach

c. Punish

d. Do nothing

4. **In a just culture, what is the appropriate action to be taken for reckless behavior?**

a. Console

b. Coach

c. Punish

d. Do nothing

5. 67 year old woman with HTN, hyperlipidemia and hepatitis C and heart murmur was seen for a preoperative evaluation for cataract surgery in January. Her cataract surgery was postponed until after her heart murmur was evaluated.

She was seen in February for evaluation of the heart murmur. A transthoracic echo shows aortic stenosis. Cardiology recommends a cardiac catheterization for evaluation. In late April, the patient has a cardiac catheterization which shows severe aortic stenosis. Aortic valve replacement is recommended. She has a preoperative appointment in mid-May where preoperative labs are drawn (CBC, basic metabolic panel, coagulation indices). The provider orders the labs on a downtime paper form and not in Epic. The downtime form did not have the ordering provider’s name. All providers in this area use downtime forms when ordering labs. The patient was admitted a few days later for the valve replacement and discharge a week later, uneventfully. In late September, the department of health calls to follow up on a positive HIV lab test result. The HIV test was drawn as part of the preoperative labs.

The patient is promptly called and given the results.

The director of risk is asking what should done be done with the front line provider in this case who ordered the test. In a hospital with a Just Culture, this provider should be:

1. Disciplined
2. Coached
3. Consoled

6. A nightfloat intern in July is called by a nurse because a delirious patient pulled out an IV. The patient had hypokalemia and needed repletion. The intern decided to put in an NGT to replete the potassium. The intern placed the NGT and then ordered the oral potassium to be given. Forty minutes later the patient became hypoxic and it was found that the NGT was in the lung. During the debrief, the intern mentioned that she had never placed an NGT on her own before or received simulation training. The nightfloat senior was busy doing an admission. There was a culture to not appear “weak.”

What type of behavior was this?

1. Human Error
2. Reckless behavior

7. A nightfloat intern in July is called by a nurse because a delirious patient pulled out an IV. The patient had hypokalemia and needed repletion. The intern decided to put in an NGT to replete the potassium. The intern placed the NGT and then ordered the oral potassium to be given. Forty minutes later the patient became hypoxic and it was found that the NGT was in the lung. During the debrief, the intern mentioned that she had never placed an NGT on her own before or received simulation training. The nightfloat senior was busy doing an admission. There was a culture to not appear “weak.”

1. What should be done?
2. Console the intern
3. Coach the intern
4. Fire the intern
5. Reprimand the intern

**Post Session Evaluation Items:**

Scale: poor (1), fair (2), good (3), very good (4), excellent (5)

*Please rate the quality of the following sessions*:

**Just culture lecture**

Circle your response:

poor (1)

fair (2)

good (3)

very good (4)

excellent (5)

**Just culture small group**

Circle your response:

poor (1)

fair (2)

good (3)

very good (4)

excellent (5)
