# Supplementary material for: Just Culture for Medical Students: Understanding Response to Providers in Adverse Events
Source: MedEdPORTAL. 2021 Jul 9;17:11167. doi: 10.15766/mep_2374-8265.11167 (PMC8266940; doi:10.15766/mep_2374-8265.11167)
Supplement: Supplementary file 1 — Slides for Cases.pptxLecture Slides.pptxFaculty Guide.docxQuiz and Evaluation Items.docx [file mep_2374-8265.11167-s001.zip › B. Lecture Slides.pptx]

## Slide 1
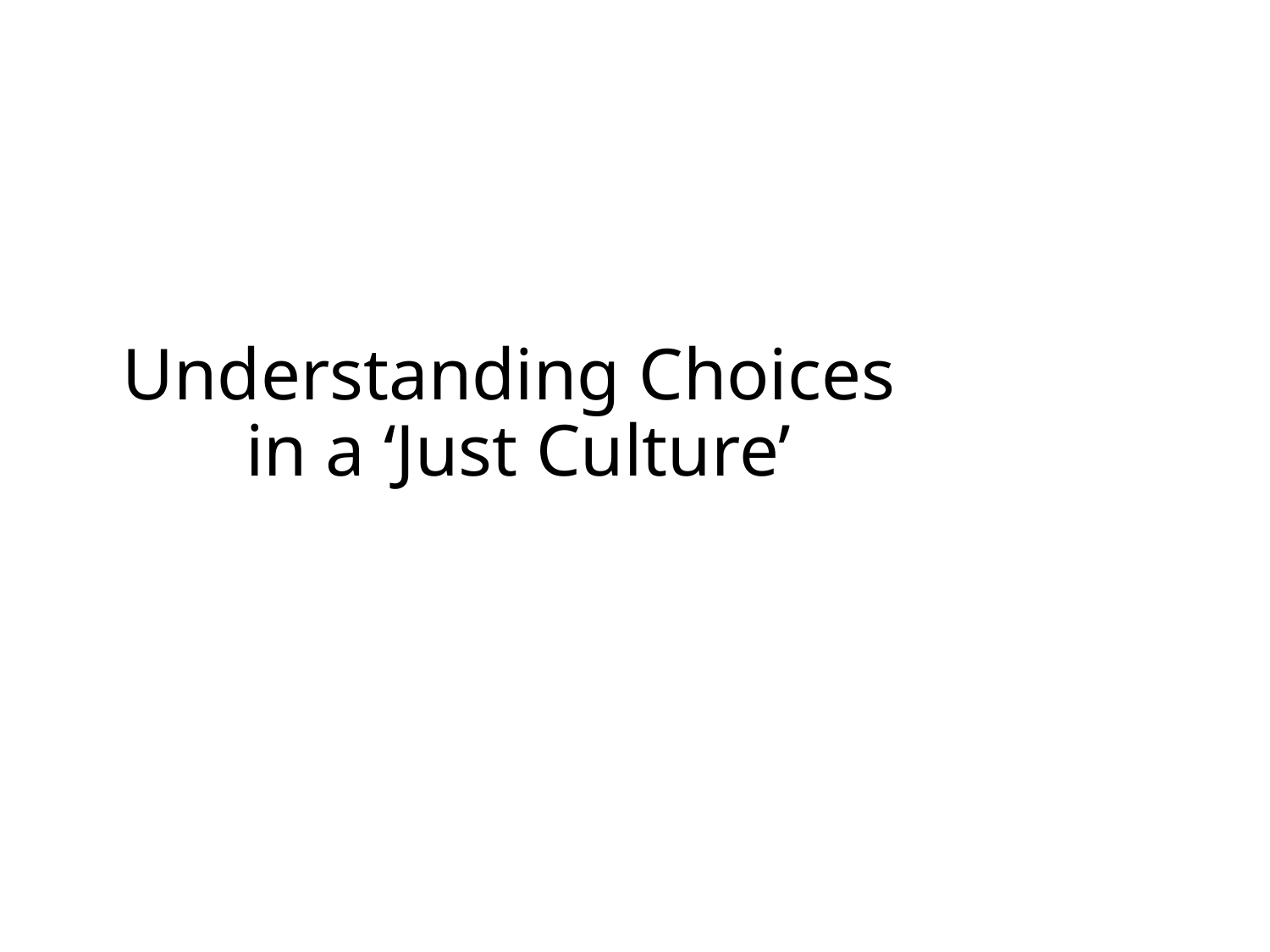

# Understanding Choices in a ‘Just Culture’

## Slide 2
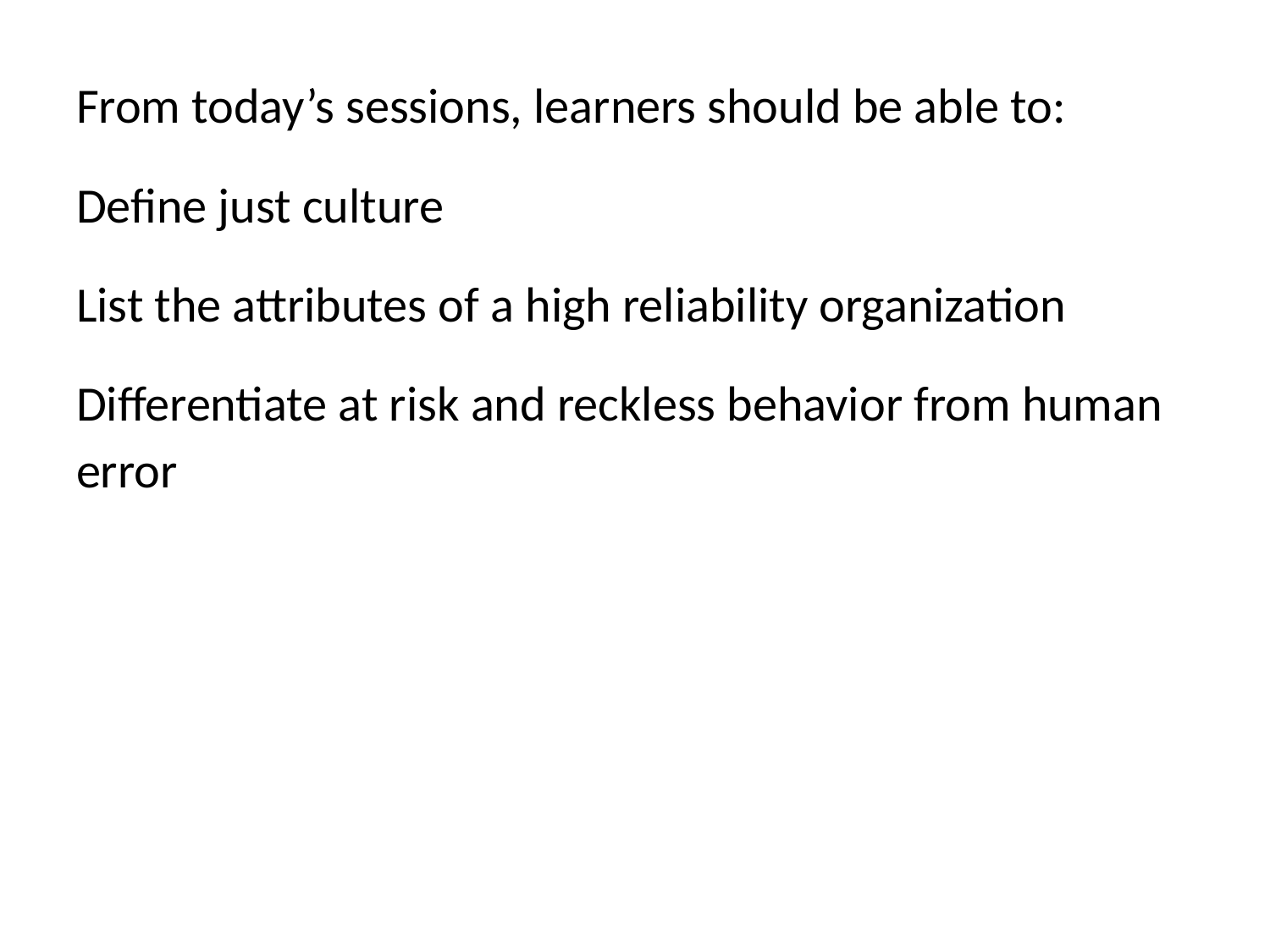

From today’s sessions, learners should be able to:
Define just culture
List the attributes of a high reliability organization
Differentiate at risk and reckless behavior from human error

## Slide 3
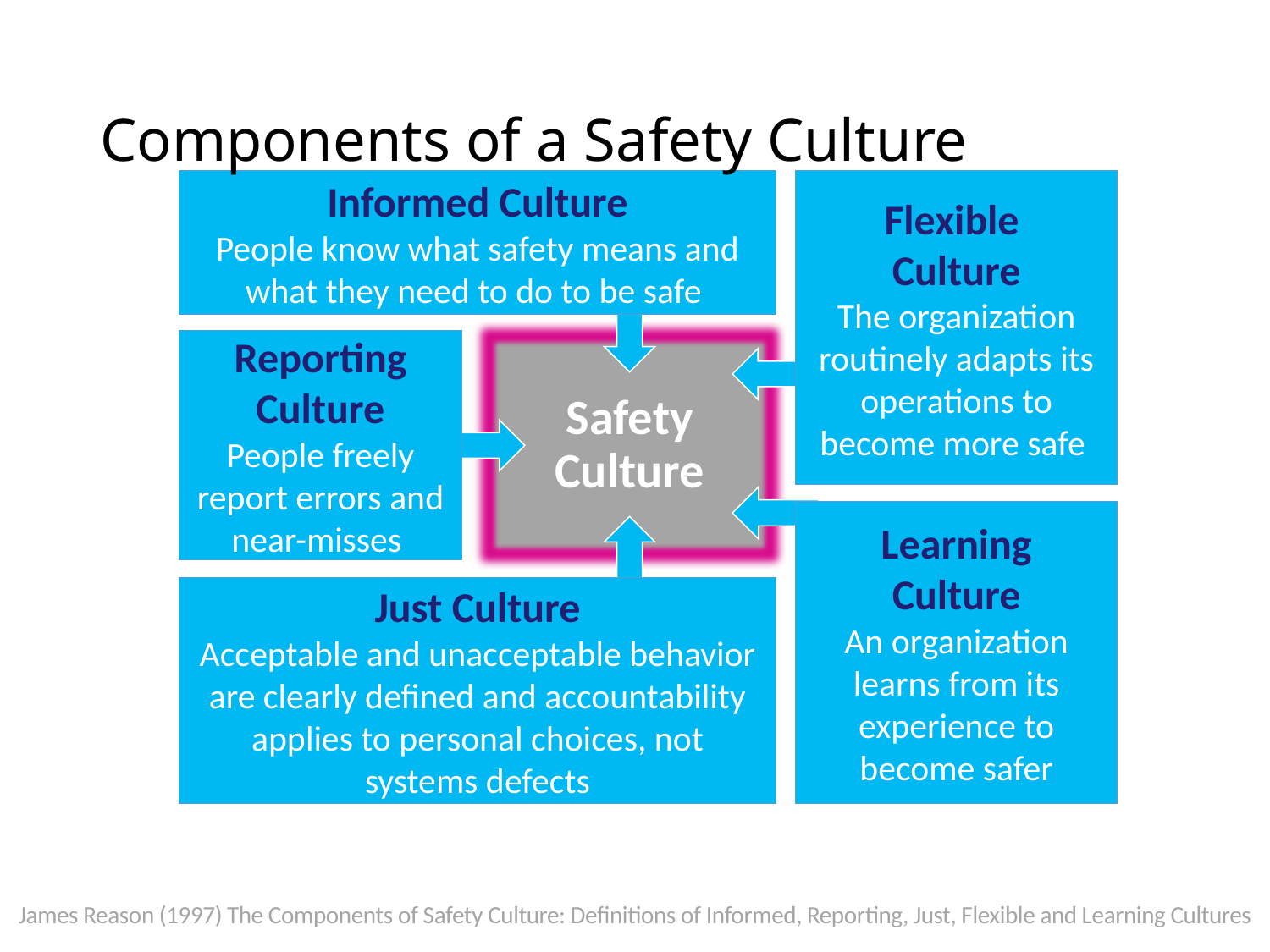

# Components of a Safety Culture
Informed Culture
People know what safety means and what they need to do to be safe
Flexible
Culture
The organization routinely adapts its operations to become more safe
Reporting Culture
People freely report errors and near-misses
Safety Culture
Learning
Culture
An organization learns from its experience to become safer
Just Culture
Acceptable and unacceptable behavior are clearly defined and accountability applies to personal choices, not systems defects
James Reason (1997) The Components of Safety Culture: Definitions of Informed, Reporting, Just, Flexible and Learning Cultures

## Slide 4
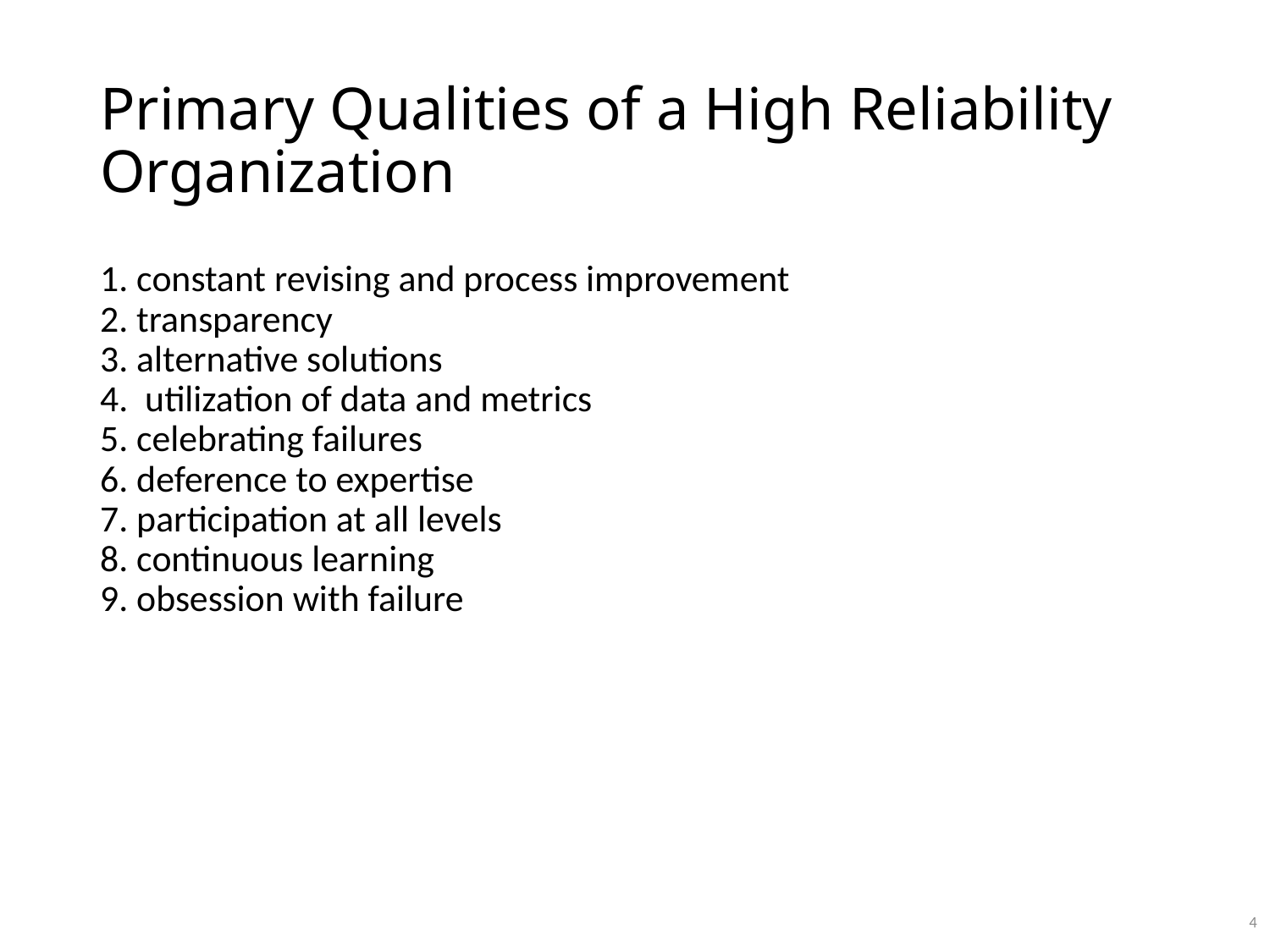

# Primary Qualities of a High Reliability Organization
1. constant revising and process improvement
2. transparency
3. alternative solutions
4. utilization of data and metrics
5. celebrating failures
6. deference to expertise
7. participation at all levels
8. continuous learning
9. obsession with failure
4

## Slide 5
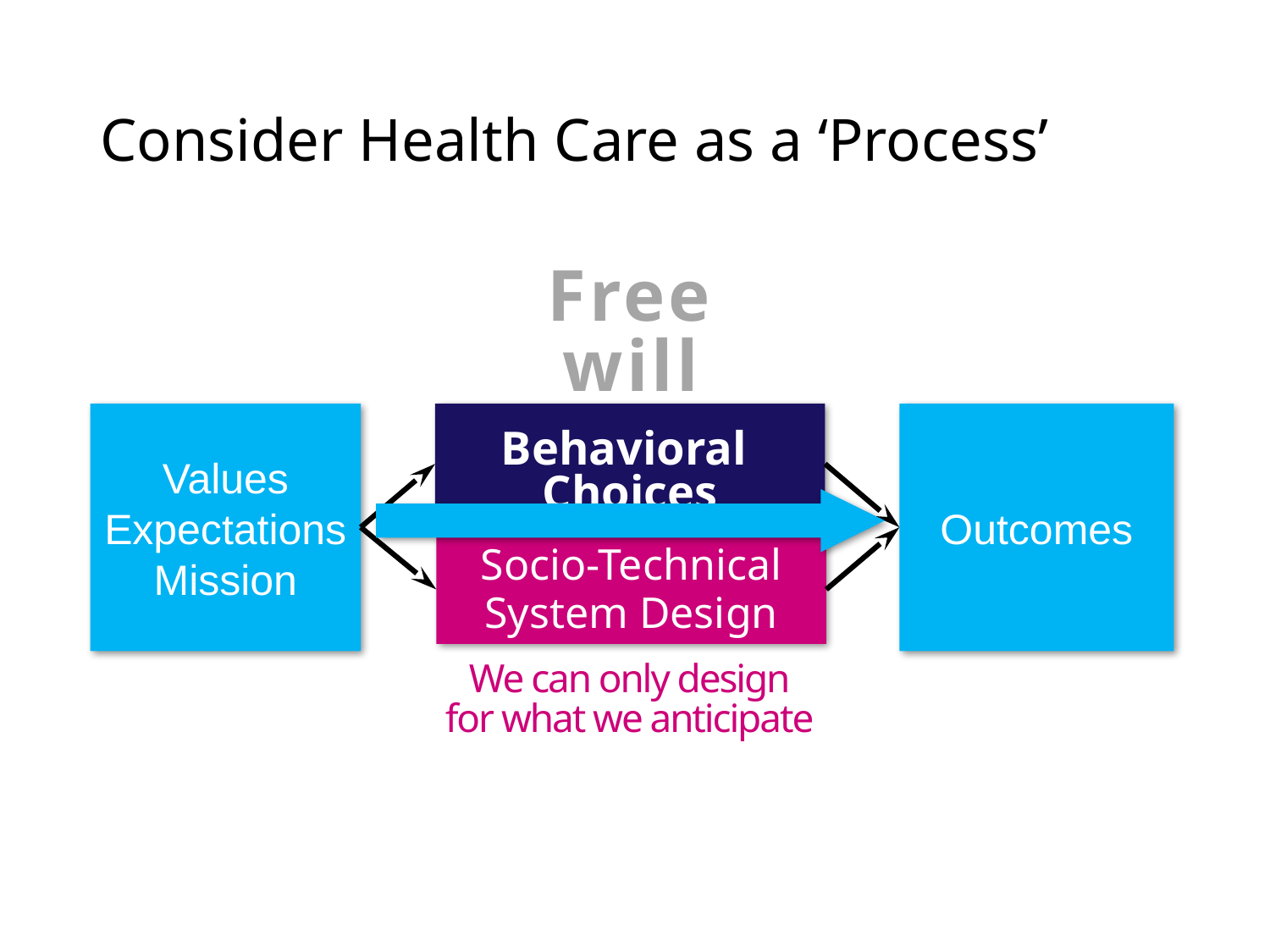

Your text here
# Consider Health Care as a ‘Process’
Free will
Outcomes
Values
Expectations Mission
Behavioral
Choices
Socio-Technical System Design
We can only design
for what we anticipate

## Slide 6
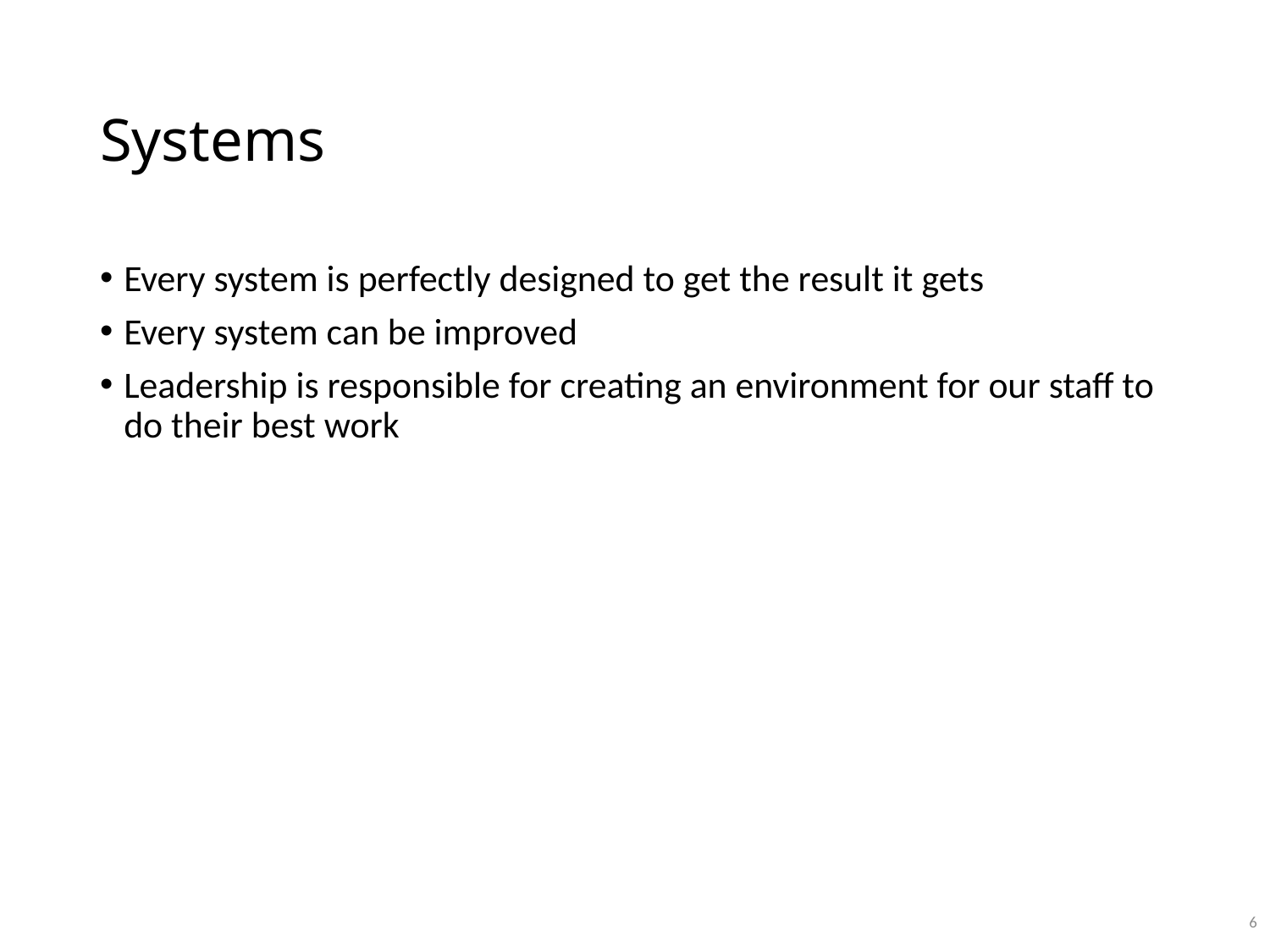

# Systems
Every system is perfectly designed to get the result it gets
Every system can be improved
Leadership is responsible for creating an environment for our staff to do their best work
6

## Slide 7
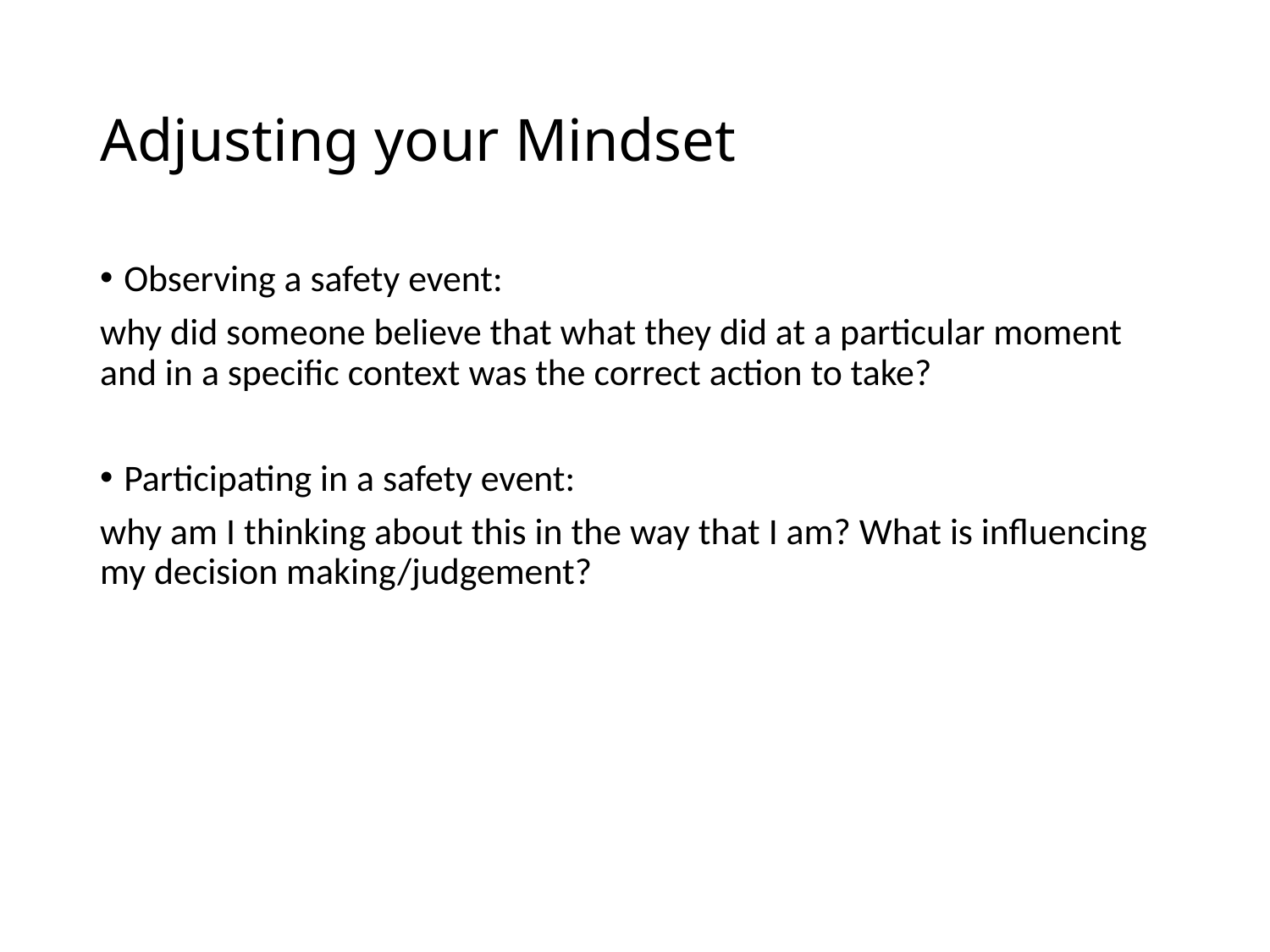

# Adjusting your Mindset
Observing a safety event:
why did someone believe that what they did at a particular moment and in a specific context was the correct action to take?
Participating in a safety event:
why am I thinking about this in the way that I am? What is influencing my decision making/judgement?

## Slide 8
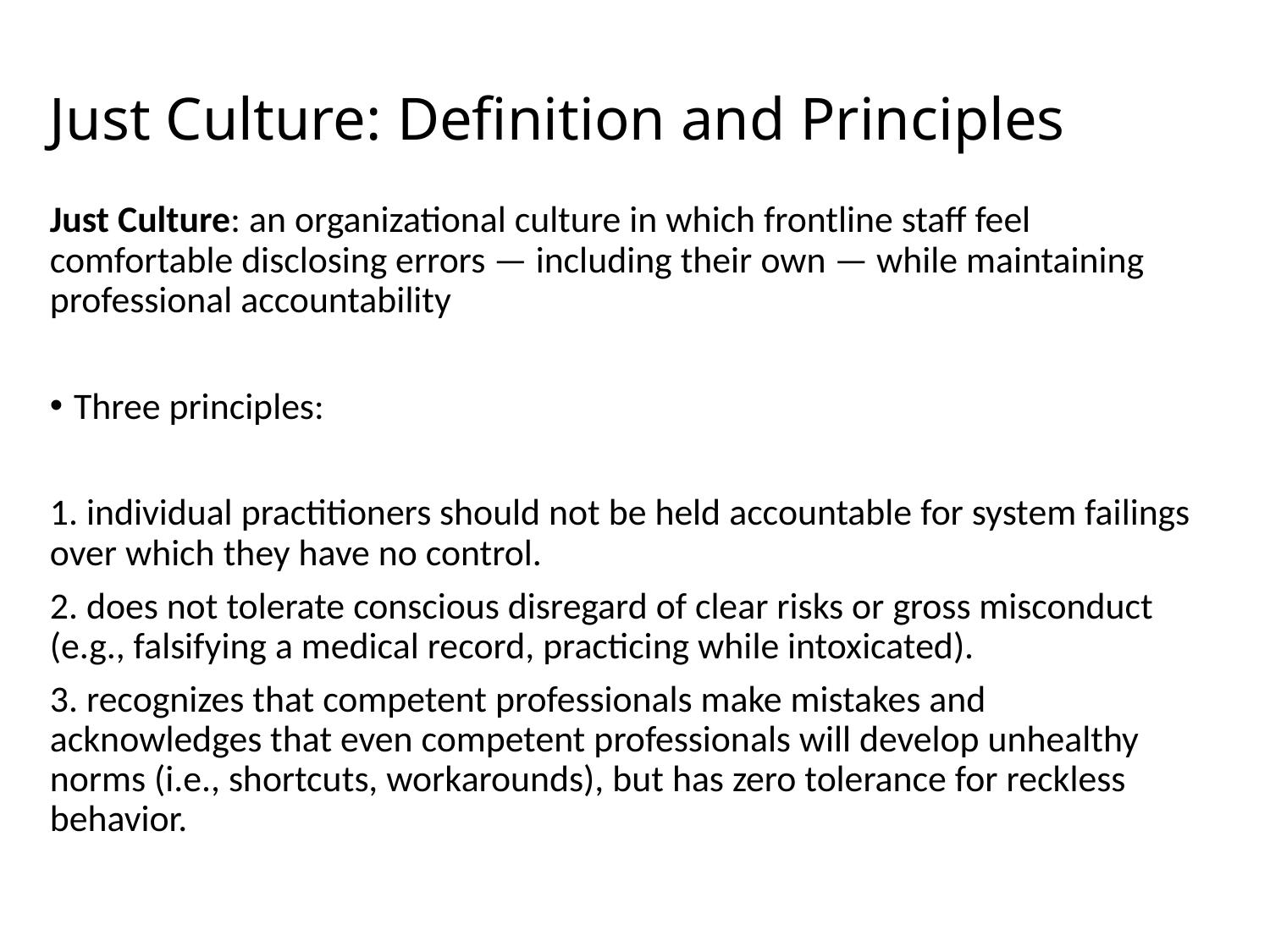

# Just Culture: Definition and Principles
Just Culture: an organizational culture in which frontline staff feel comfortable disclosing errors — including their own — while maintaining professional accountability
Three principles:
1. individual practitioners should not be held accountable for system failings over which they have no control.
2. does not tolerate conscious disregard of clear risks or gross misconduct (e.g., falsifying a medical record, practicing while intoxicated).
3. recognizes that competent professionals make mistakes and acknowledges that even competent professionals will develop unhealthy norms (i.e., shortcuts, workarounds), but has zero tolerance for reckless behavior.

## Slide 9
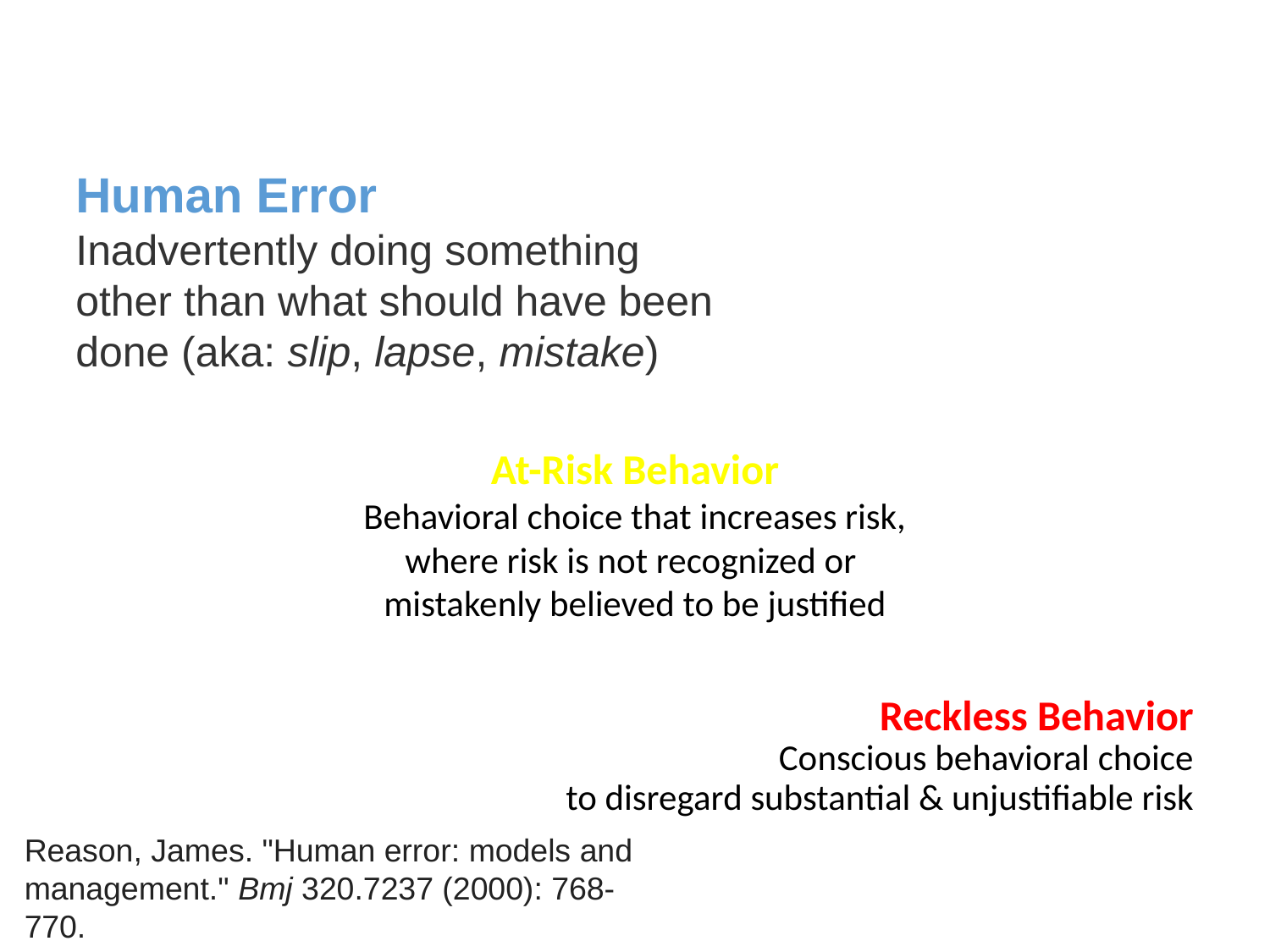

Human Error
Inadvertently doing something
other than what should have been done (aka: slip, lapse, mistake)
At-Risk Behavior
Behavioral choice that increases risk,
where risk is not recognized or
mistakenly believed to be justified
Reckless Behavior
Conscious behavioral choice
to disregard substantial & unjustifiable risk
Reason, James. "Human error: models and management." Bmj 320.7237 (2000): 768-770.

## Slide 10
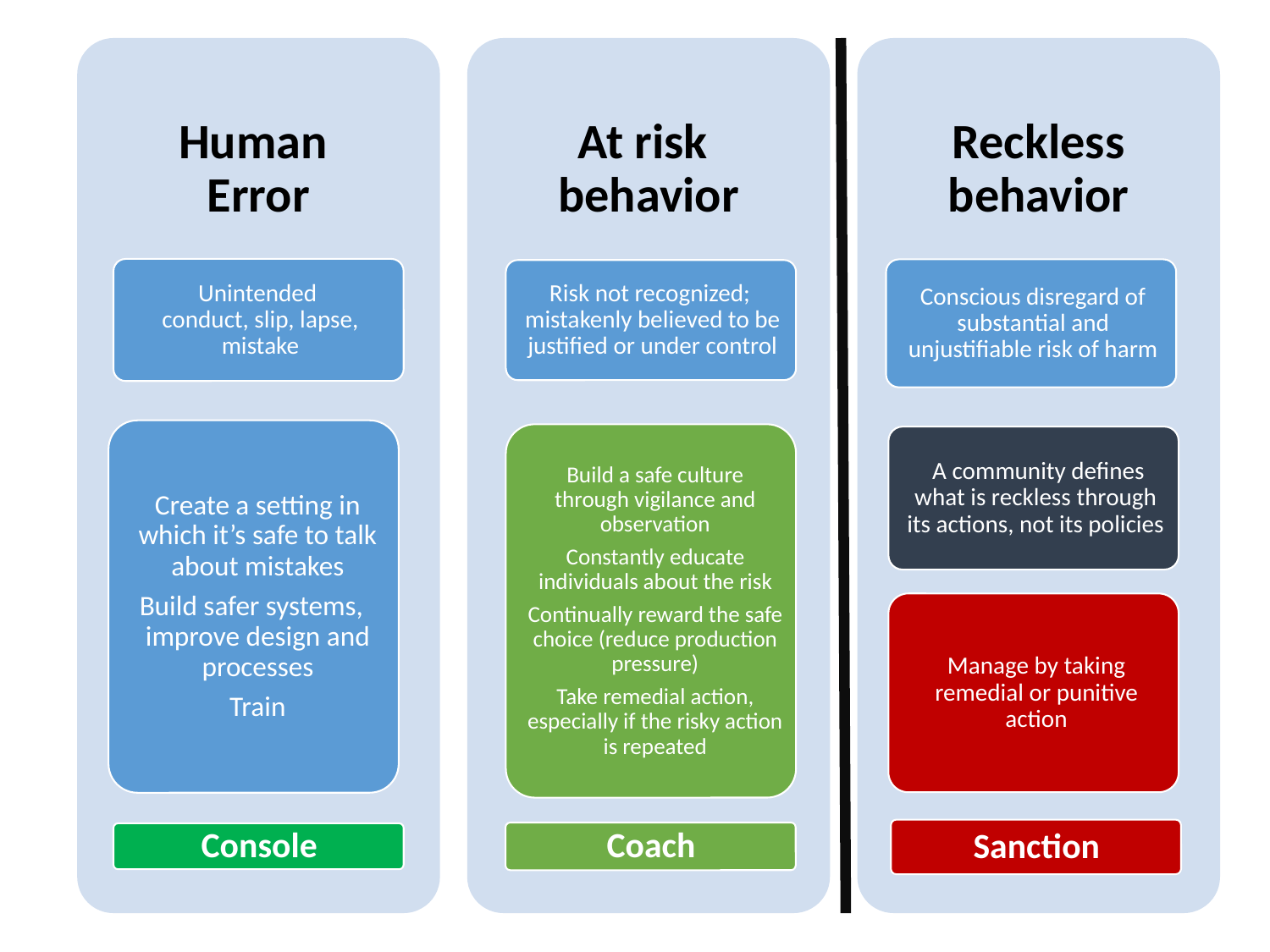

## Slide 11
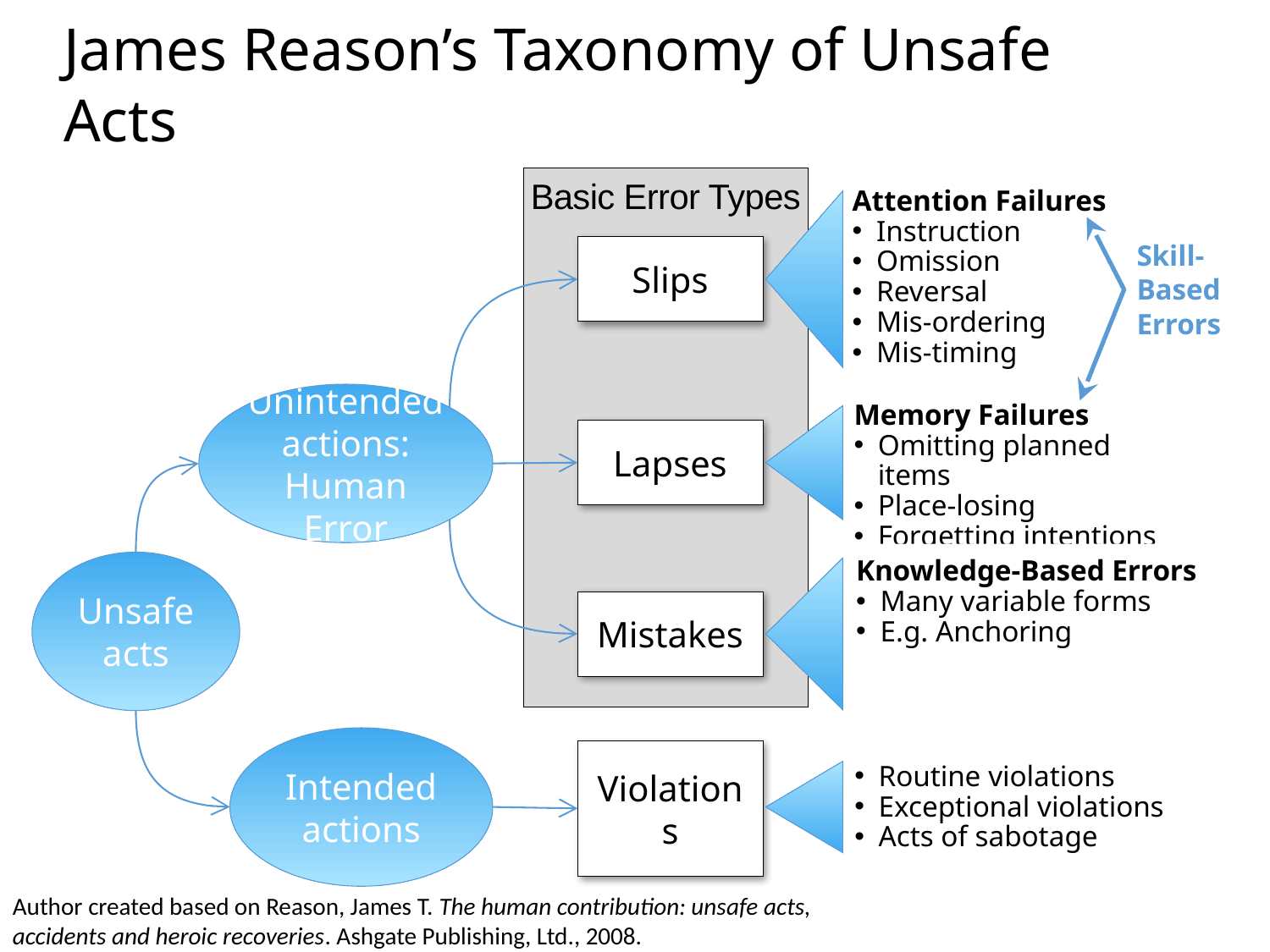

# James Reason’s Taxonomy of Unsafe Acts
Basic Error Types
Attention Failures
Instruction
Omission
Reversal
Mis-ordering
Mis-timing
Slips
Skill-Based
Errors
Unintended actions:
Human Error
Memory Failures
Omitting planned items
Place-losing
Forgetting intentions
Lapses
Knowledge-Based Errors
Many variable forms
E.g. Anchoring
Unsafe
acts
Mistakes
Intended actions
Routine violations
Exceptional violations
Acts of sabotage
Violations
Author created based on Reason, James T. The human contribution: unsafe acts, accidents and heroic recoveries. Ashgate Publishing, Ltd., 2008.

## Slide 12
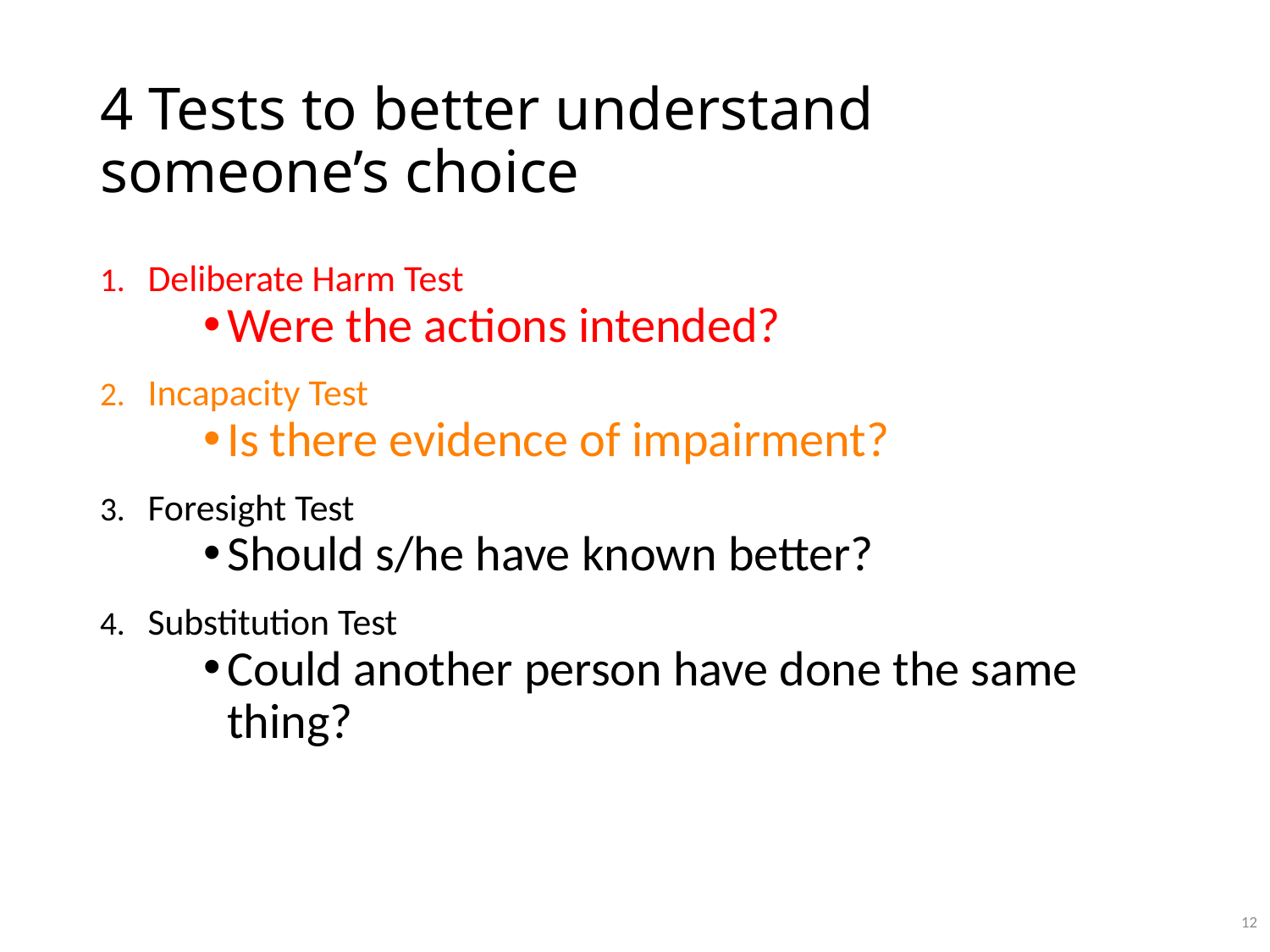

# 4 Tests to better understand someone’s choice
Deliberate Harm Test
Were the actions intended?
Incapacity Test
Is there evidence of impairment?
Foresight Test
Should s/he have known better?
Substitution Test
Could another person have done the same thing?
12

## Slide 13
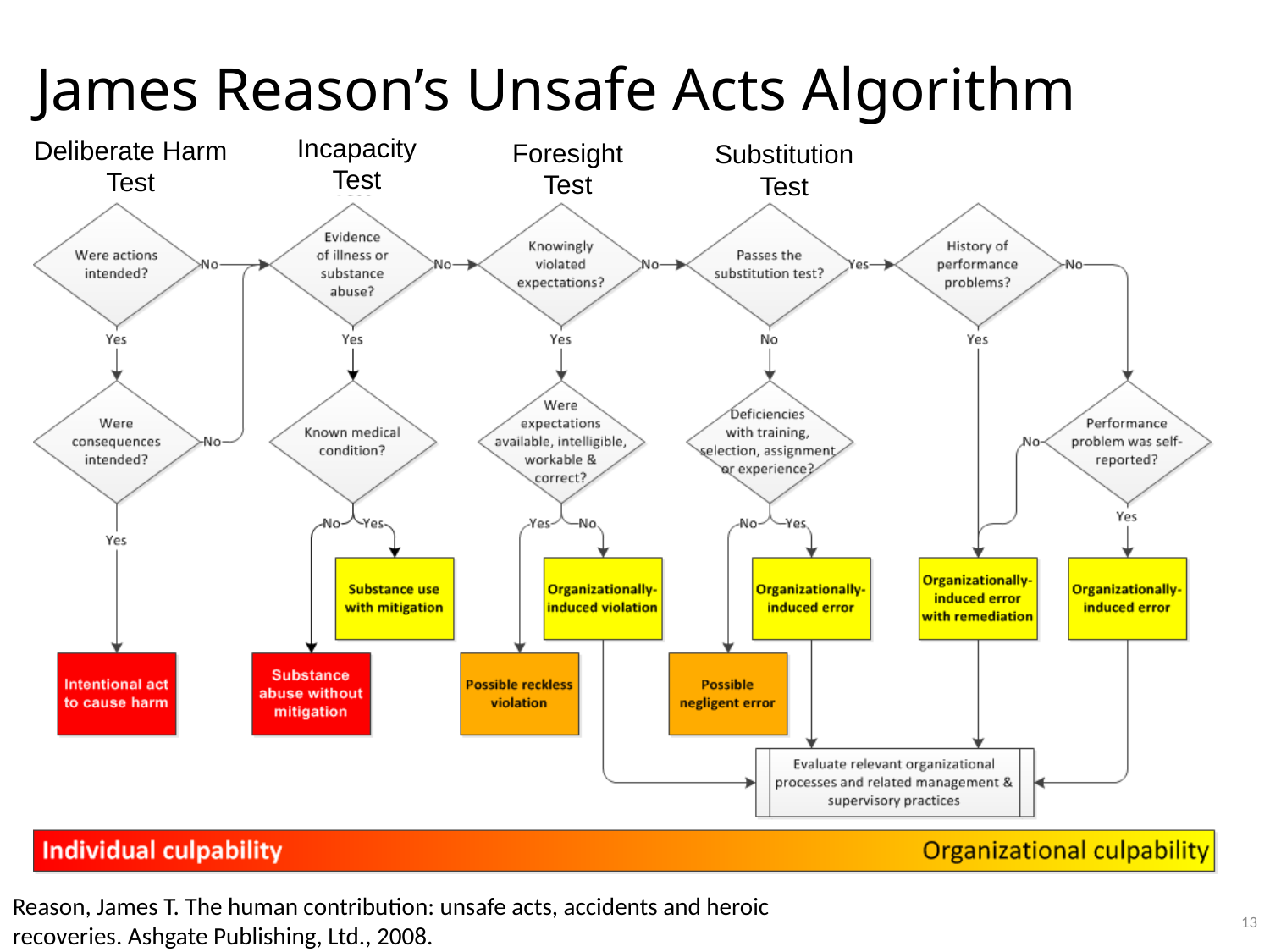

# James Reason’s Unsafe Acts Algorithm
Incapacity
Test
Deliberate Harm Test
Foresight
Test
Substitution
Test
Reason, James T. The human contribution: unsafe acts, accidents and heroic recoveries. Ashgate Publishing, Ltd., 2008.
13

## Slide 14
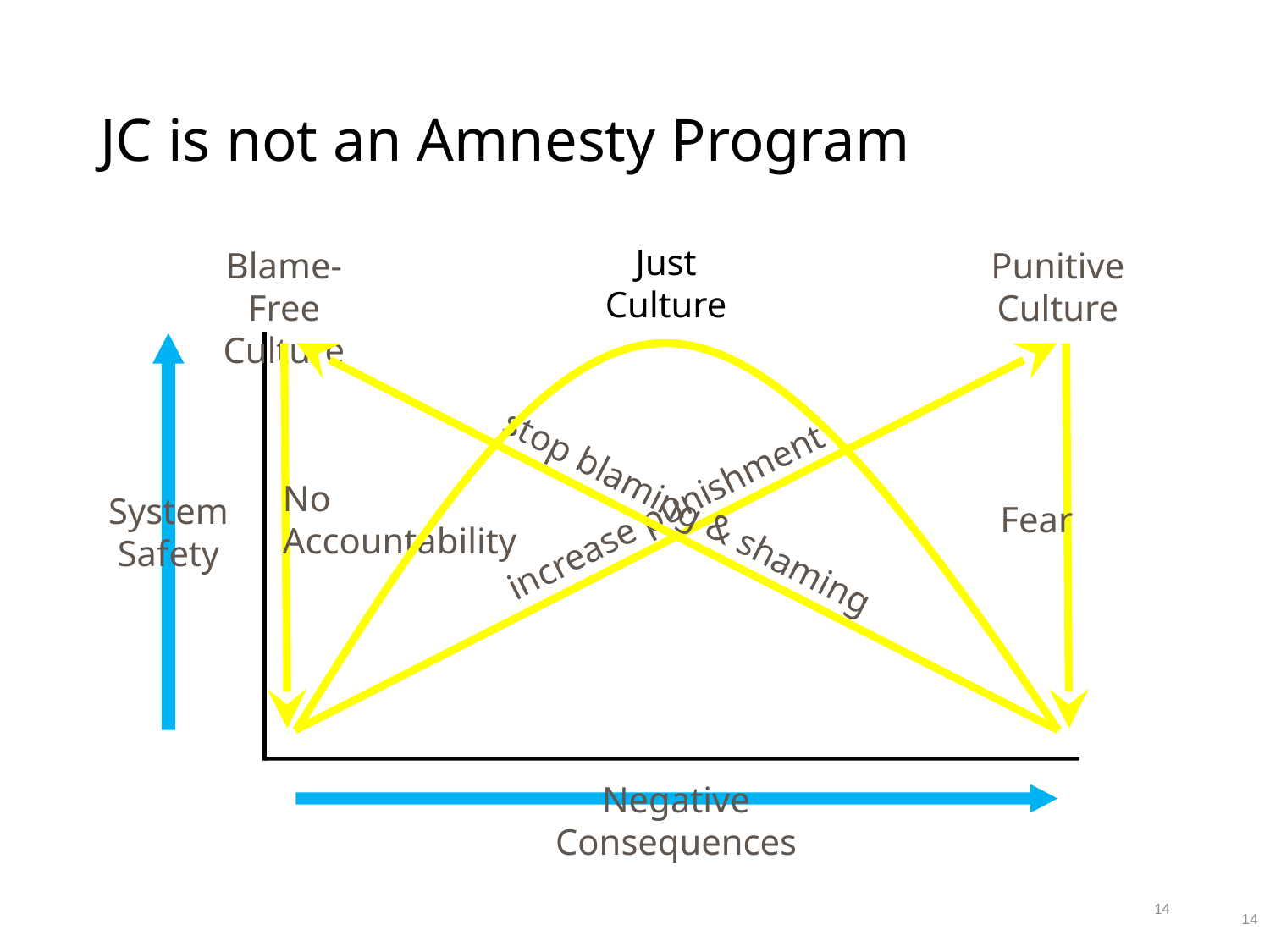

# JC is not an Amnesty Program
Just
Culture
Blame-Free Culture
Punitive Culture
System Safety
No Accountability
increase punishment
stop blaming & shaming
Fear
Negative Consequences
14
14

## Slide 15
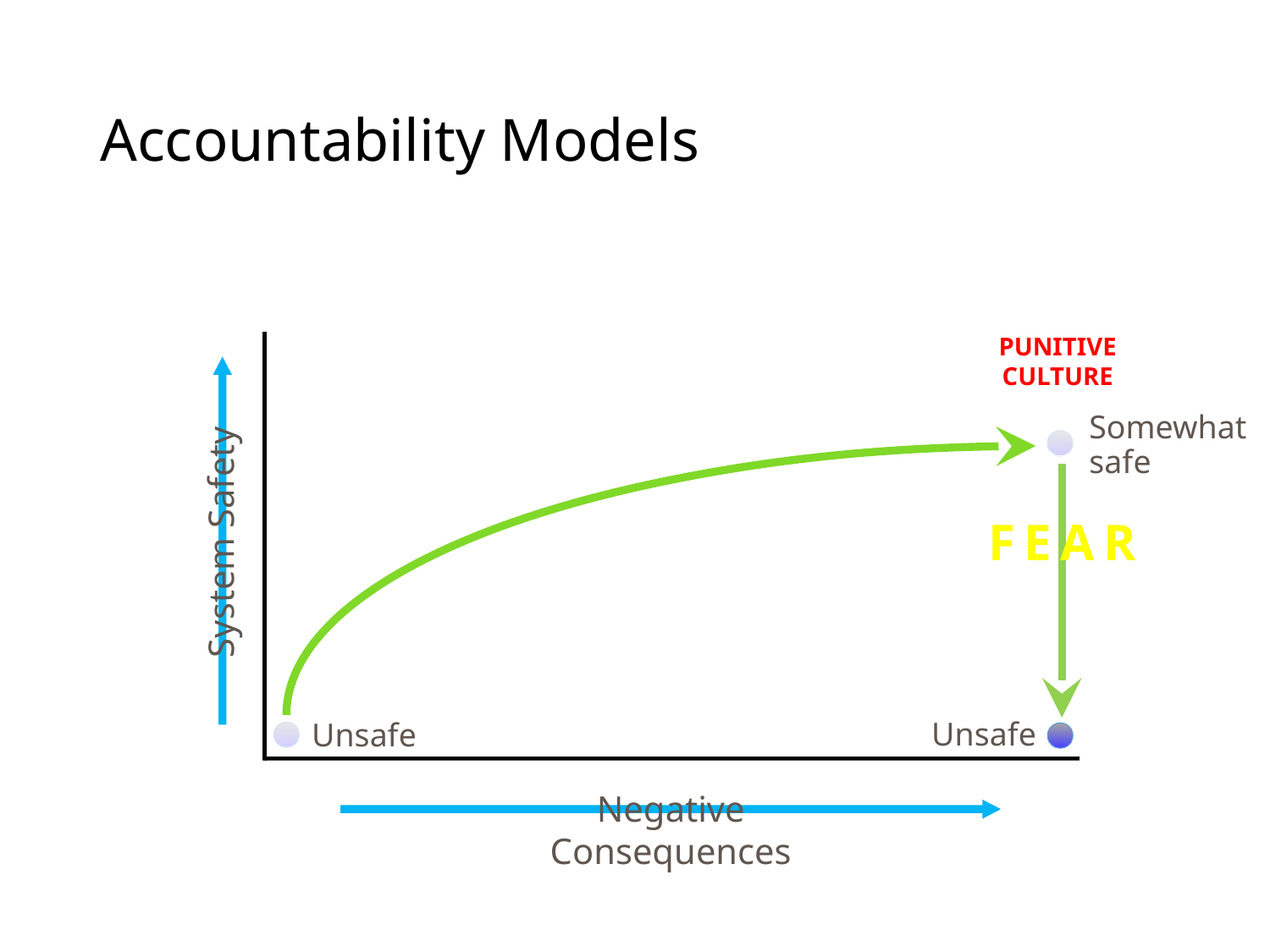

# Accountability Models
PUNITIVE CULTURE
System Safety
Somewhat
safe
FEAR
Unsafe
Unsafe
Negative Consequences

## Slide 16
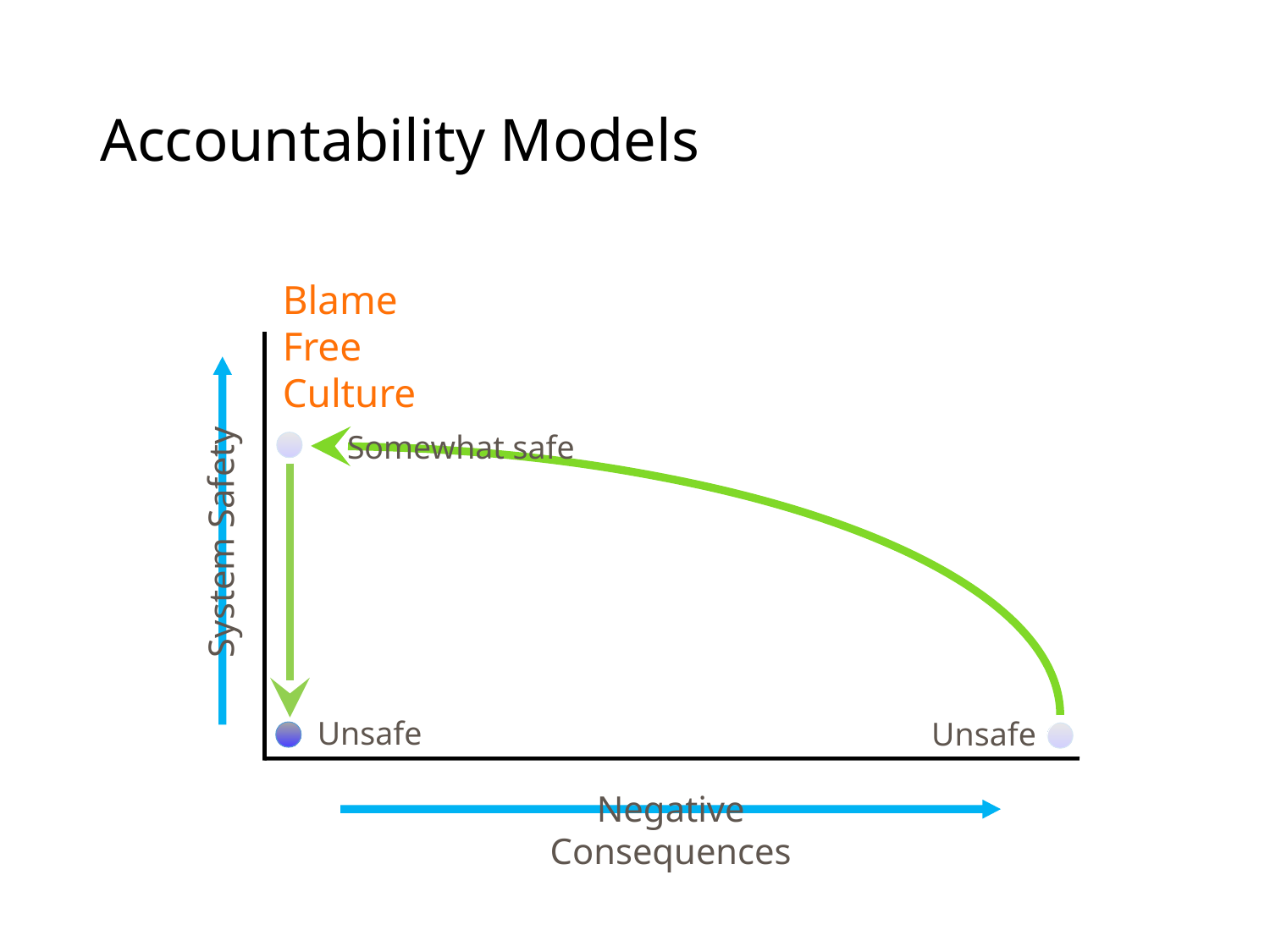

# Accountability Models
Blame
Free Culture
System Safety
Somewhat safe
Unsafe
Unsafe
Negative Consequences

## Slide 17
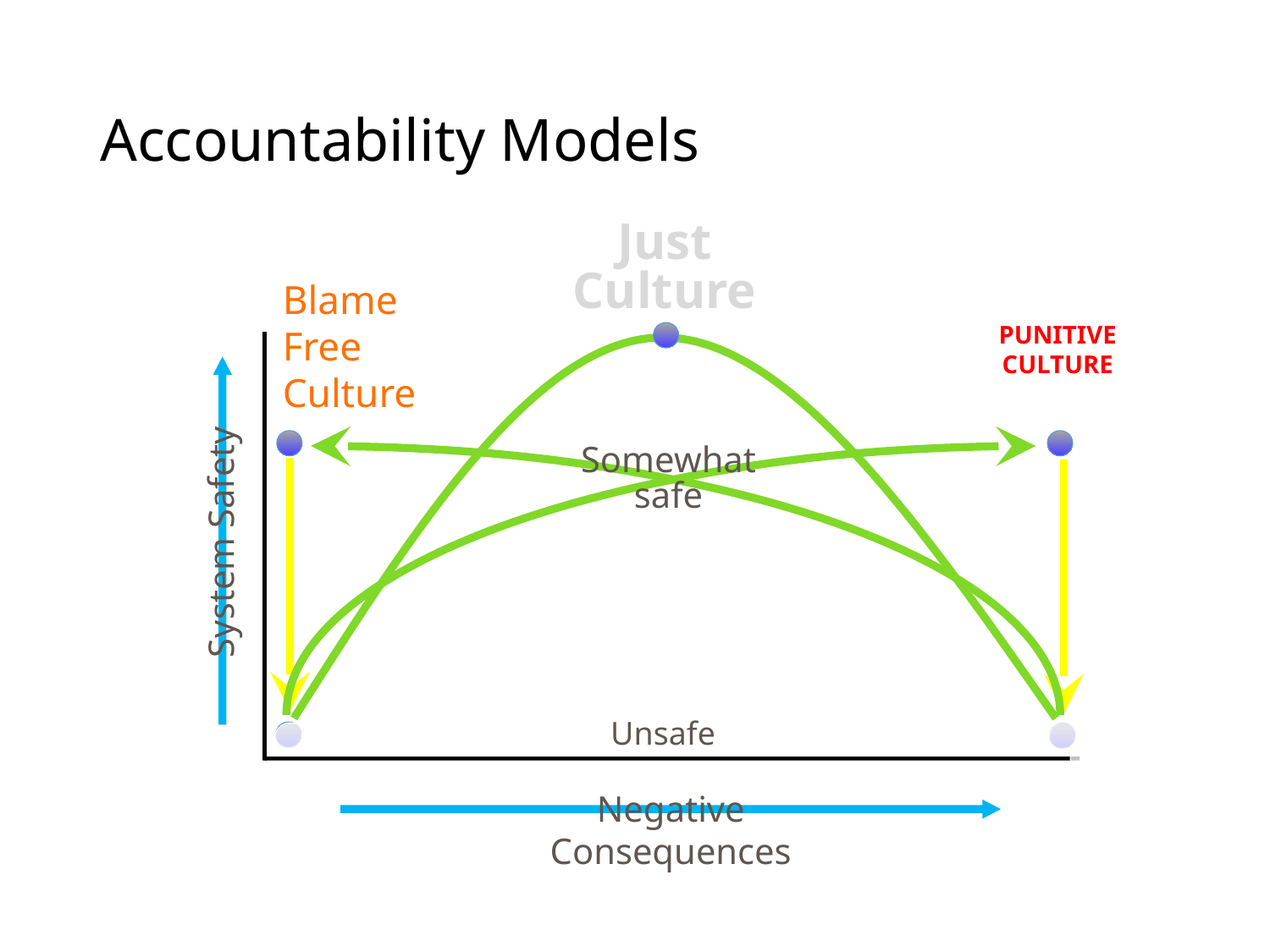

# Accountability Models
Just
Culture
Blame
Free Culture
PUNITIVE CULTURE
System Safety
Somewhat
safe
Unsafe
Negative Consequences

## Slide 18
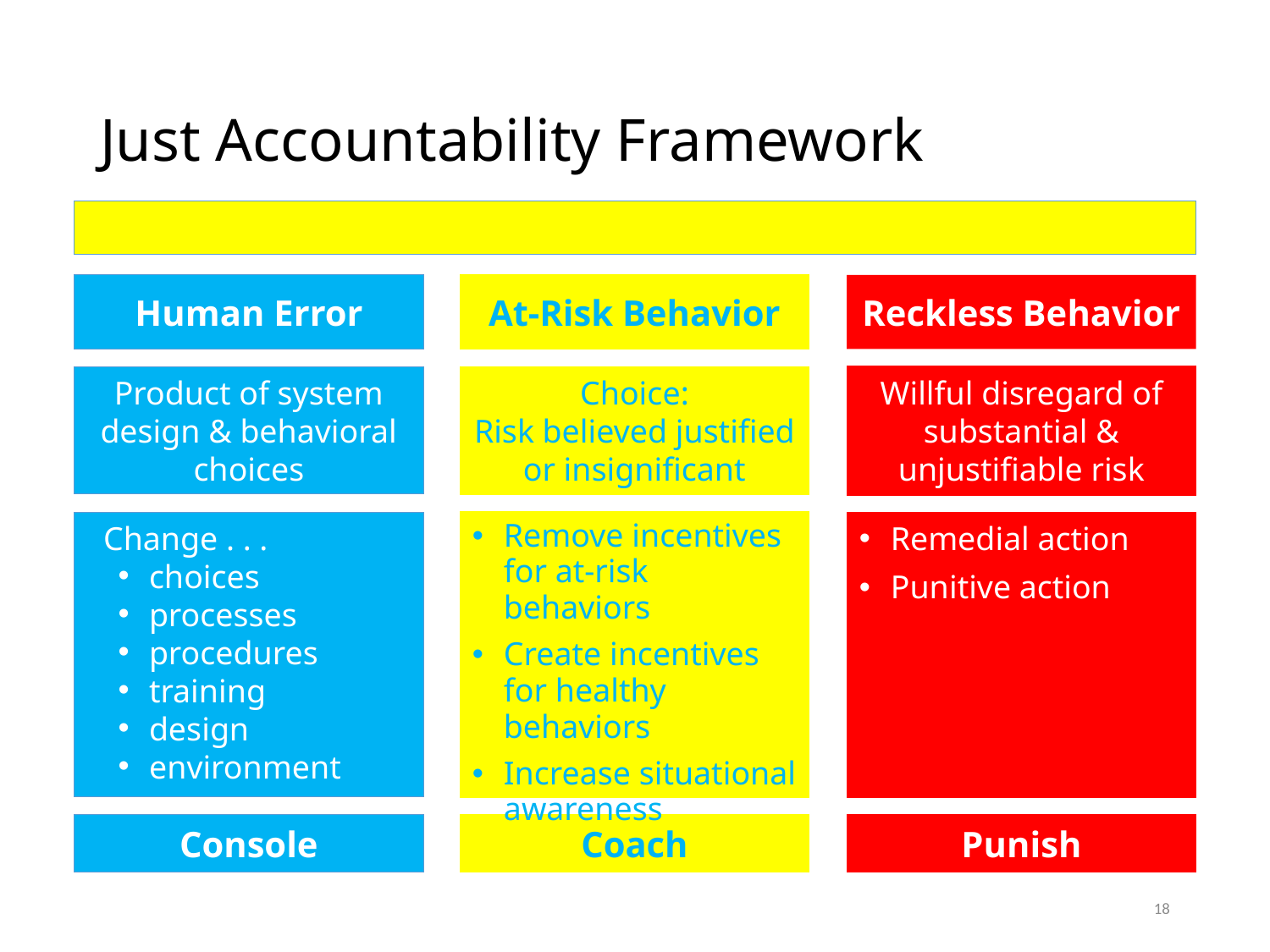

# Just Accountability Framework
Human Error
At-Risk Behavior
Reckless Behavior
Willful disregard of substantial & unjustifiable risk
Choice:
Risk believed justified or insignificant
Product of system design & behavioral choices
Remove incentives for at-risk behaviors
Create incentives for healthy behaviors
Increase situational awareness
Change . . .
choices
processes
procedures
training
design
environment
Remedial action
Punitive action
Console
Coach
Punish
18

## Slide 19
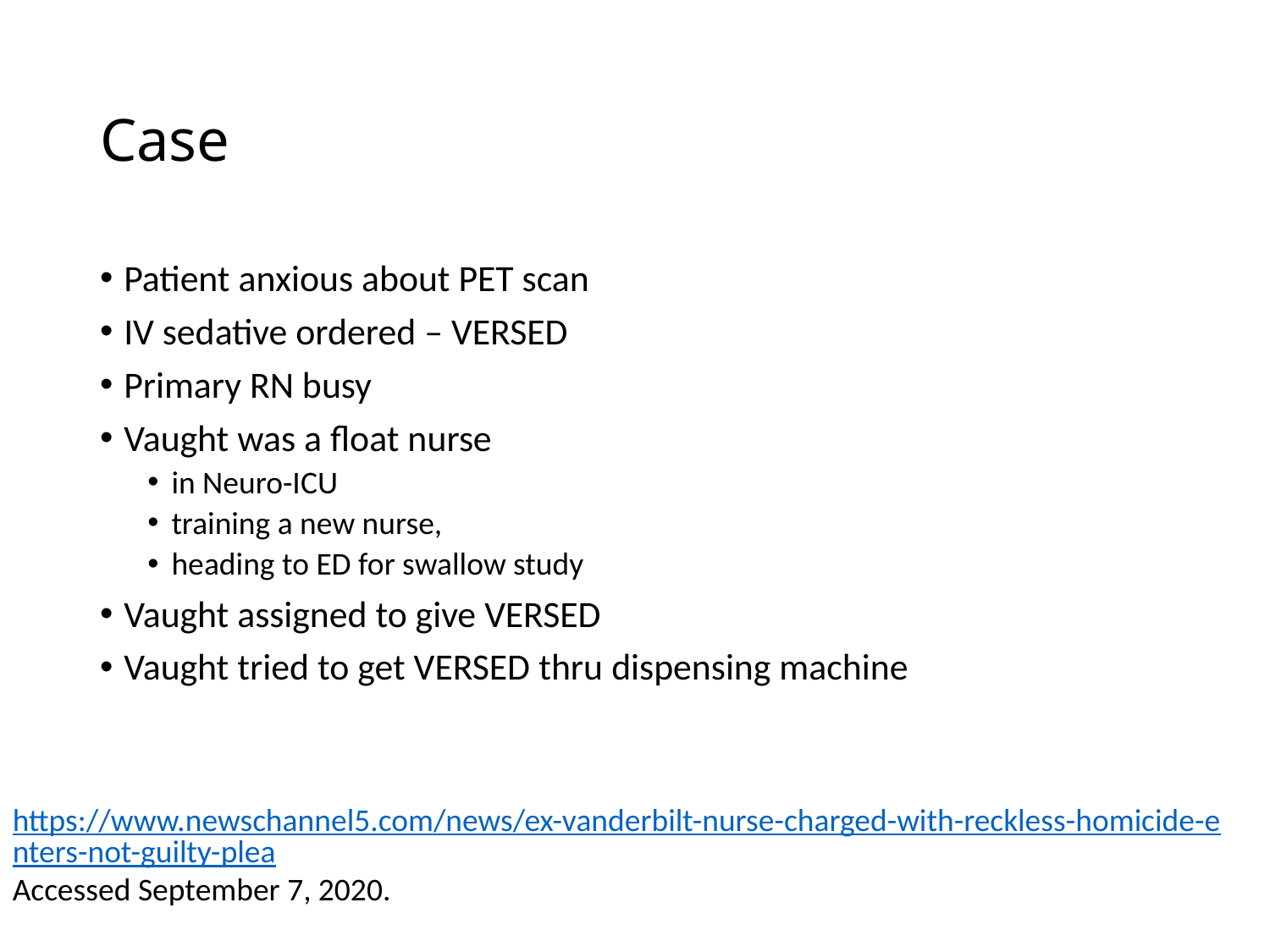

# Case
Patient anxious about PET scan
IV sedative ordered – VERSED
Primary RN busy
Vaught was a float nurse
in Neuro-ICU
training a new nurse,
heading to ED for swallow study
Vaught assigned to give VERSED
Vaught tried to get VERSED thru dispensing machine
https://www.newschannel5.com/news/ex-vanderbilt-nurse-charged-with-reckless-homicide-enters-not-guilty-plea
Accessed September 7, 2020.

## Slide 20
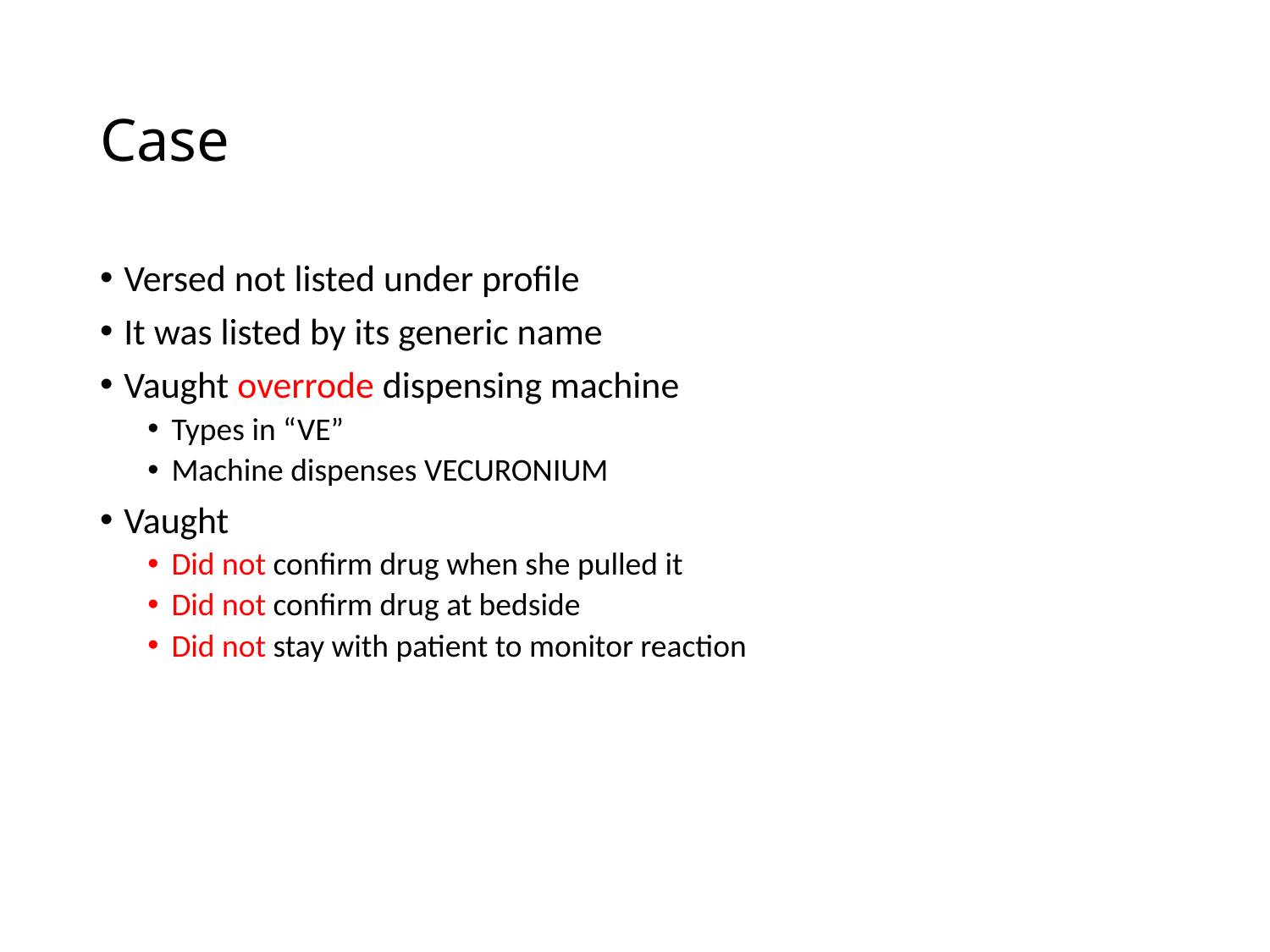

# Case
Versed not listed under profile
It was listed by its generic name
Vaught overrode dispensing machine
Types in “VE”
Machine dispenses VECURONIUM
Vaught
Did not confirm drug when she pulled it
Did not confirm drug at bedside
Did not stay with patient to monitor reaction

## Slide 21
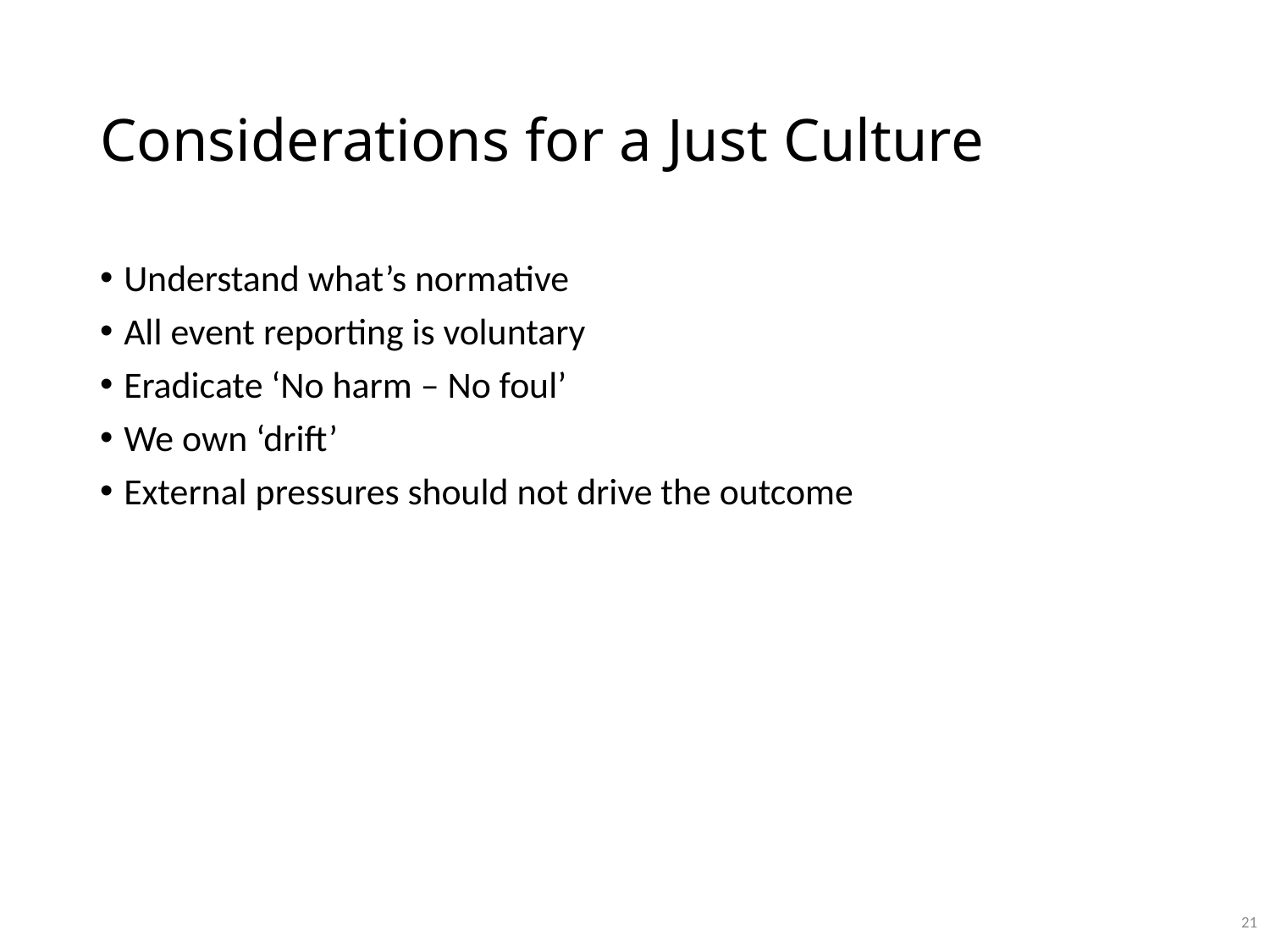

# Considerations for a Just Culture
Understand what’s normative
All event reporting is voluntary
Eradicate ‘No harm – No foul’
We own ‘drift’
External pressures should not drive the outcome
21

## Slide 22
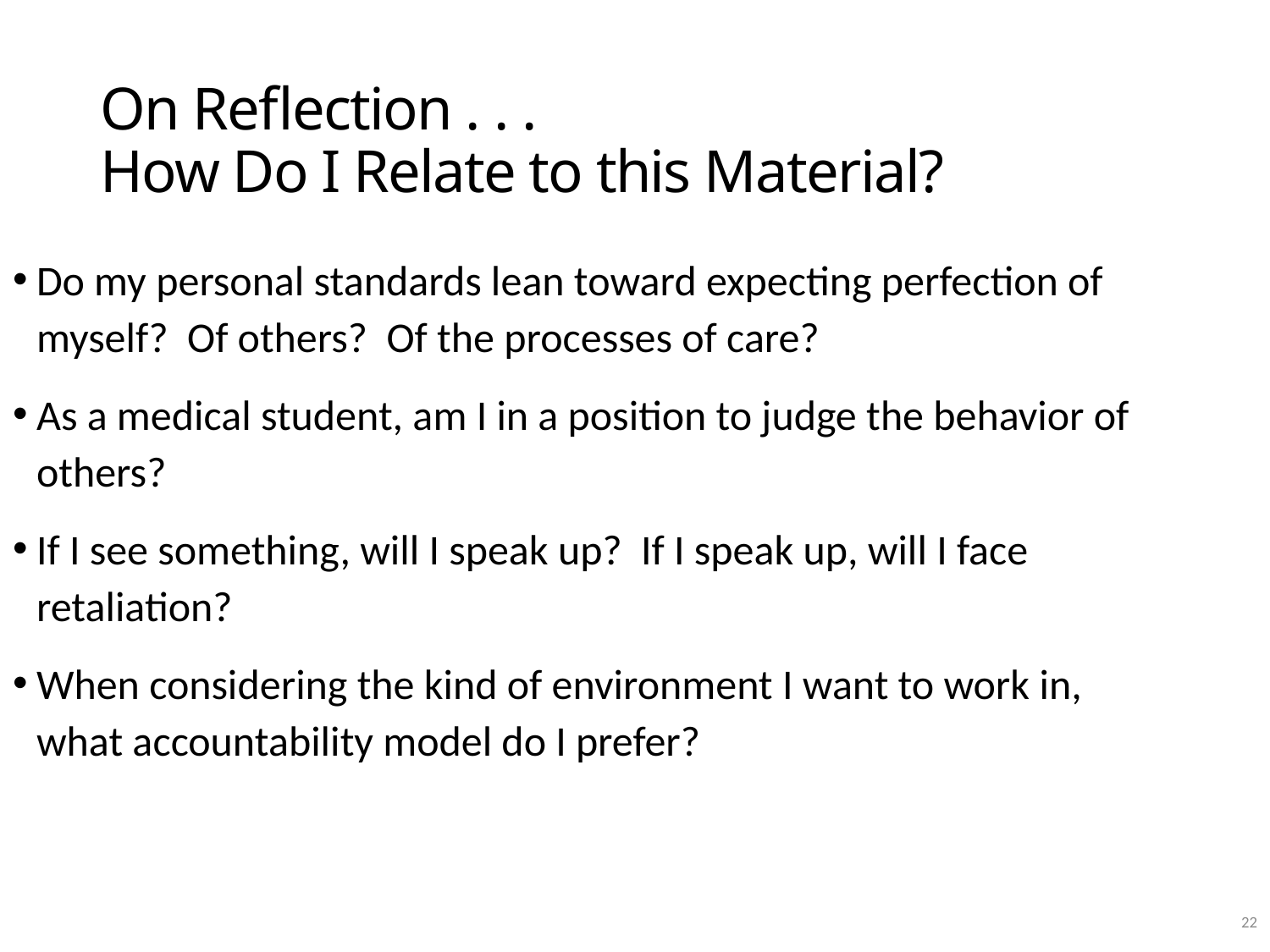

# On Reflection . . . How Do I Relate to this Material?
Do my personal standards lean toward expecting perfection of myself? Of others? Of the processes of care?
As a medical student, am I in a position to judge the behavior of others?
If I see something, will I speak up? If I speak up, will I face retaliation?
When considering the kind of environment I want to work in, what accountability model do I prefer?
22
