# Supplementary material for: Just Culture for Medical Students: Understanding Response to Providers in Adverse Events
Source: MedEdPORTAL. 2021 Jul 9;17:11167. doi: 10.15766/mep_2374-8265.11167 (PMC8266940; doi:10.15766/mep_2374-8265.11167)
Supplement: Supplementary file 1 — Slides for Cases.pptxLecture Slides.pptxFaculty Guide.docxQuiz and Evaluation Items.docx [file mep_2374-8265.11167-s001.zip › Educational_Summary_Report_11167.pdf]

MedEdPORTAL® The AAMC Journal of Teaching and Learning Resources

Original Publication Open Access

Just Culture for Medical Students: Understanding Response to Providers in Adverse Events

Brijen J. Shah, MD*, Bonnie Portnoy, RN, Dennis Chang, MD, Marc Napp, MD

*Corresponding Author: brijen.shah@mssm.edu

Abstract

Introduction: Individual and organizational response to an adverse event is a key part of the life cycle of a patient safety event. Just culture is a safety concept that emphasizes system drivers of human behavior. We developed a learning activity for medical students to teach and discuss just culture as part of a patient safety curriculum. **Methods:** This small-group, discussion-based learning activity was aimed at third-year medical students. Over 5 years, 628 students participated in it. The session had three components: a presession case-based survey, a didactic lecture, and a facilitated small-group discussion. Participants evaluated the session using our institution's standard learner assessment. They also took a postcourse test that contained multiple-choice questions relating to the session. **Results:** On a 5-point Likert scale (1 = *poor,* 3 = *good,* 5 = *excellent*), students rated the large-group lecture (3.2) and small-group discussion (3.2) moderately. Over 85% of students answered all knowledge items on a course posttest correctly. **Discussion:** This learning activity provides an easy-to-implement case-based discussion to introduce the concepts of just culture.

Keywords
Adverse Event, Patient Safety, Health Systems, Quality Improvement/Patient Safety, Systems-Based Practice, Case-Based Learning

Educational Objectives

By the end of this session, learners will be able to:

1. Define just culture.
2. List the attributes of a high-reliability organization.
3. Differentiate at-risk and reckless behavior from human error.
4. Recognize how errors differ in the degree of individual accountability and the responses required to address their undesirable outcomes.
5. Describe factors that create a culture that encourages speaking up.

Introduction

A pillar of high reliability, just culture is the conceptual approach to achieving an open and honest reporting culture while fostering appropriate accountability for the choices that humans make when interacting with a system.[1,2] Just culture dictates the institutional, day-to-day response to patient safety events that is separate from the malpractice process, to which students almost certainly have had more exposure due to popular media coverage. Just culture examines the roles of personal choices and sociotechnical systems. In so doing, it draws attention to systems explanations for patient safety events rather than personal blameworthy causes. For health care learners, education and training around just culture involve knowledge transfer, application of these principles to events, and changes in attitude around blame in the context of patient safety.

Citation:
Shah BJ, Portnoy B, Chang D, Napp M. Just culture for medical students: understanding response to providers in adverse events. *MedEdPORTAL*. 2021;17:11167.
https://doi.org/10.15766/mep_2374-8265.11167

Over the last 10 years, UME and GME curricula have included safety science knowledge and patient safety analysis tools. The majority of these curricula focus on event reporting, event analysis, and development of corrective actions. To be complete, learning about the patient safety life cycle must include how the health system responds to the involved staff both formally and informally—just culture. The Institute for Healthcare Improvement Open School offers a module (PS 203) that considers the concept of just culture through a case scenario.[3] However, that self-paced learning tool does not allow medical students an opportunity to discuss the challenging concepts of just culture. A recent PubMed search did not show any undergraduate medical school curricula or learning resources focused on just culture. There

Copyright © 2021 Shah et al. This is an open-access publication distributed under the terms of the Creative Commons Attribution license.

were two papers discussing application of just culture in nursing school for nursing students.[4,5]

In *MedEdPORTAL,* four resources mention just culture in their materials.[6-9] Two of these are focused on GME learners and faculty.[6,8] One UME resource mentions just culture; however, its learning materials focus on event analysis and provide no introduction of the just culture framework.[7] Cumbler and Glasheen provide a GME-focused resource that describes the types of human errors but does not discuss how to apply the just culture framework to event analysis.[9]

There are management training programs designed to build knowledge and skills about just culture, emphasizing how to respond to safety events.[10,11] However, the context of these programs requires human resources knowledge and patient safety experience that are not accessible to learners new to patient safety science. Most students at this point in their education have not yet been exposed to an adverse event, and so, a patient safety curriculum should (a) illustrate how high-reliability organizations ensure just accountability for human actions and (b) help students develop a just mindset to shape their reactions to patient safety events when serving as leaders of health care teams. Just culture helps to move a medical student's or resident's reaction away from blaming and toward seeking systems explanations and solutions.

Papers on health care quality improvement and patient safety curricula have identified nonpunitive response to error and systems approaches to error analysis, an element of the just culture framework, as a topic that should be included in the undergraduate curriculum.[12] The USMLE requires content addressing the role of law in health care, but this frequently translates into teaching about malpractice risks,[13] not the just culture concept of system factors that drive humans to make certain choices. Here, we present a discussion-based learning activity that provides undergraduate medical students with the opportunity to apply just culture concepts and engage in discussion about the complexity of these topics as part of a larger patient safety curriculum for third-year students. Our resource offers educators materials to create an individual learning gap and a structure for group discussion to aid students in their development of a just mindset.

Methods

This resource was a small-group, discussion-based learning activity for third-year medical students. It was part of InFocus, a larger sequence of health care delivery science modules that included human and system errors, event reporting, quality improvement, and reducing medical malpractice (Figure). Our session could also be used for more advanced learners engaged in a patient safety curriculum.

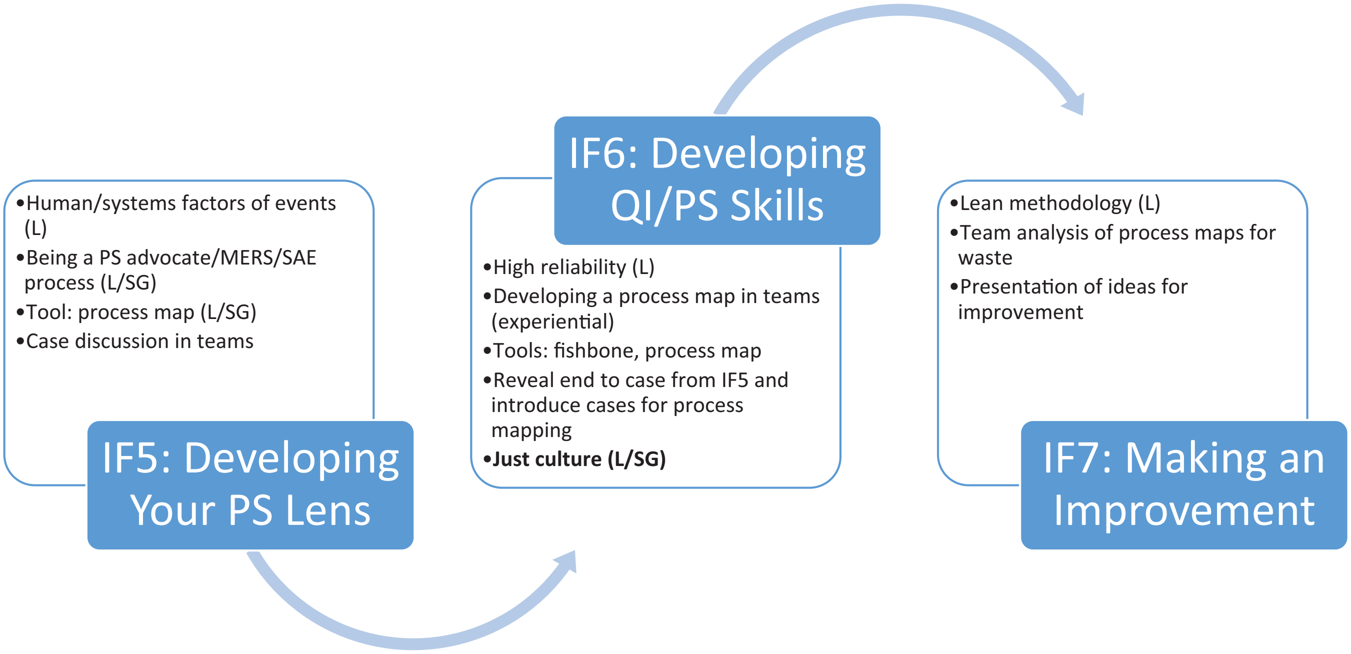

Figure. Partial InFocus course schematic: QI/PS via doing. The just culture session is indicated in bold. Abbreviations: IF5, InFocus module 5; IF6, InFocus module 6; IF7, InFocus module 7; L, lecture; MERS, Medical Event Reporting Systems; PS, patient safety; QI, quality improvement; SAE, serious adverse event; SG, small group.

Copyright © 2021 Shah et al. This is an open-access publication distributed under the terms of the Creative Commons Attribution license.

The session had three components: a presession case-based survey, a didactic lecture, and a facilitated small-group discussion. Because most students were likely to have had little experience with adverse events or the concept of just culture, we used a case-based social constructivist approach to inform our design.

Prior to the session and the provision of any didactic content, learners received a survey link, via SurkeyMonkey, leading them to a series of five case scenario descriptions (Appendix A). Each case scenario focused on a behavior or situation that a clinical student would encounter. These case scenarios were based on actual events that had been observed or reported in our institution. For each case scenario, using terms derived from the just culture algorithm,[1,2] learners were asked to rate the level of risk that the behavior posed to the patient, identify the type of behavior, and indicate how the behavior should be addressed. For one of the cases, we adapted a survey approach from Martinez et al. to stimulate a discussion about the various hierarchical health care team member roles and their impact on individual responses to at-risk behaviors.[14] Before the small-group session, these responses were aggregated across the class cohort and graphically displayed on slides.

A 50-minute lecture (Appendix B) preceded the small-group discussion. Topics covered included the concept of just culture; definitions of human error, at-risk behavior, and reckless behavior; and appropriate responses to those actions, that is, consoling, coaching, and punishing, respectively. We presented this information in the context of responding to adverse events, which students had learned in a prior part of the course.

Following the lecture, learners participated in a 50-minute small-group discussion utilizing the five case scenarios. The session was facilitated by faculty members with training in just culture and included patient safety leaders, risk managers, and chief medical officers from across our health system. In each small-group room, a slide deck contained the case scenarios and aggregated class responses to support the discussion.

The small groups ranged in size from 12 to 30 students over the years this session was offered. We started with larger groups due to fewer faculty and over time developed more faculty, so that our groups grew closer to 12-14 students each. This size allowed for greater discussion and participation during the discussion. We used a roundtable format when the sessions were in person and, more recently, have done this in a virtual forum with the same size groups. Rooms were equipped with a computer that allowed for sharing slides.

A faculty guide (Appendix C) provided prompts for discussion to encourage participation. For each case scenario, we developed key teaching points highlighting various aspects of just culture. The guide served to standardize the delivery of the learning content and to assist faculty in facilitating a discussion on a topic unfamiliar to medical students. We conducted 30-minute group faculty development sessions for all faculty the week prior to the small-group sessions. Those faculty who could not attend spoke directly with the authors for a one-on-one faculty development session.

We assessed students' learning using a few multiple-choice knowledge questions given at the end of this portion of the course (in 2015, 2018, and 2019) or at the conclusion of the entire course (in 2016 and 2017). These questions were created by the authors and adjusted, if needed, based on the performance on each item. For 2018-2019, we created a case scenario asking two questions; this change was made to assess application of knowledge rather than recall. A learner assessment used for all undergraduate medical school courses at our institution was given to participants to evaluate the session. Responses were on a 5-point Likert scale (1 = *poor,* 3 = *good,* 5 = *excellent*). The assessment was administered 1 week after the conclusion of the course (Appendix D). Because the curriculum leadership had deemed this course to be pass/fail, student participation in the small groups was assessed only via submission of the presession survey and attendance at the small group.

Results

The course was taken by 673 learners. There was a 100% completion rate on the multiple-choice items, which were part of a larger course posttest. Over 85% of students answered the knowledge items from the multiple-choice exam correctly (Table 1). Learners were able to define key just culture terms and identify the appropriate response for each of the three types of behavior. When the case scenario format was used for similar topics, performance appeared to decrease several percentage points.

Overall, there was a 93% response rate for the items evaluating the learning session. Learners reported moderate satisfaction with the large-group lecture and stable moderate-to-fair satisfaction with the small-group session (Table 2). Ratings for both sessions were similar despite different instructional approaches. There were limited narrative comments specific to the small-group discussion. Two illustrative comments were "Explained the concept really well and gave us time to discuss

Copyright © 2021 Shah et al. This is an open-access publication distributed under the terms of the Creative Commons Attribution license.

Table 1. Percentage of Correct Responses on Posttest Knowledge Assessment

Question	Percentages				
	2015 (*N* = 131)	2016 (*N* = 128)	2017 (*N* = 136)	2018 (*N* = 137)	2019 (*N* = 141)
What is the definition of a just culture?	96	93	95		
In a just culture, what is the appropriate action to be taken for a human error?	99	98	98		
In a just culture, what is the appropriate action to be taken for an at-risk behavior?	98	92	90		
In a just culture, what is the appropriate action to be taken for reckless behavior?	94	87	87		
The director of risk is asking what should done be done with the frontline provider in this case who ordered the test. In a hospital with a just culture, this provider should be coached, counseled, or punished?	91	91	92		
Case question with an intern who placed a nasogastric tube and there was a complication. The intern had never done this before, and there was a culture to not appear "weak."					
What type of behavior is this?				64	92
How should the program/leadership respond to the intern?				89	83

as a group without feeling the need to go through each word on each slide—much appreciated!" and "Excellent framing, really engaging, felt extremely relevant. Wouldn't change a thing." Sixty-six percent of students felt that the health system had a just culture when asked in March of the third year, as part of the end-of-year course assessment.

Discussion

We present the first learning resource focused on the concepts of just culture aimed at medical students. The session utilizes a constructivist and behaviorist approach to teach just culture concepts and foster an attitude of accountability when evaluating patient safety events. This content is part of a larger curriculum on patient safety science at our institution. We designed the session to teach the concepts of just culture and high reliability through a large-group lecture and a small-group discussion session grounded in examples within the experience and role of medical students and their next stage of training, internship and residency.

Over the course of the last 4 years, we used a continuous process improvement mindset to adjust the session based on feedback from both faculty and learners. In the main lecture, we decreased the number of concepts being taught, focusing on the three types of behavior and the responses to each. We also introduced the concept of high reliability and how just culture relates to this larger idea. In prior lectures, the content was too broad and initially did not relate back to earlier sessions on safety science, such as human and system causes of events. Feedback from faculty drove us to teach about diagnostic errors prior to the session since we referred back to such misses as an example of human error in the session. We added a brief mention of malpractice but let learners know it would be covered in a future session.

Initially, we had only three faculty members to lead the discussion sessions, resulting in approximately 50 students per group, too large to really be effective. To encourage discussion, therefore, we used Poll Everywhere technology to get input on each case scenario. This design choice provided instant feedback for each room but logistically was difficult to implement with three to four sessions running at a time. However, both faculty and students liked the use of audience response in the small-group session.

To provide more time to think about the case scenarios and focus both student and faculty effort on the discussion, we decided to send the case scenarios and questions out over an electronic survey link as part of the course prework. This design change allowed students time to self-reflect on the case scenarios and to have them in their memory as they listened to the lecture and prepared for discussion. This change allowed us to aggregate the class data and capture a greater variation in responses. We placed the aggregated responses to each case scenario question in graphical format in a PowerPoint slide deck. These slides were

Table 2. Student Satisfaction With Lecture and Small-Group Discussion

Learning Activity[a]	2015 (*N* = 129)	2016 (*N* = 142)	2017 (*N* = 105)	2018 (*N* = 124)	2019 (*N* = 128)
Lecture	3.1	3.4	3.2	3.3	3.2
Small-group discussion	3.1	N/A	3.3	3.2	3.1

[a]Rated on a 5-point Likert scale (1 = *poor*, 3 = *good*, 5 = *excellent*).

Copyright © 2021 Shah et al. This is an open-access publication distributed under the terms of the Creative Commons Attribution license.

placed in each small-group room to be used for the session. We adjusted the faculty guide to allow faculty to utilize the variation in responses to generate prompts for discussion. We feel that this design change has allowed enhanced engagement and helped to close a learning gap by applying the concepts to these case scenarios.

After the second year, we recognized the need for smaller group size in order to enhance the discussion. To accomplish this, we recruited risk managers, chief medical officers, and other patient safety leaders to help facilitate the sessions. The session size decreased to 14-15 learners per group, with the additional benefit of having faculty who could speak about their own experiences and challenges related to applying just culture in health care.

While learner satisfaction with the sessions was lower than we expected, it was in the same range as other sessions during these 3 weeks in the third year. These sessions were held between clinical rotation blocks. Session scores ranged from 3.2 to 4.0 out of 5. Challenges with this and other patient safety and quality improvement curricular content included immediate applicability to the role of the third-year student, minimal emphasis of this content on the USMLE, and the pass/fail nature of the course. Overall course satisfaction was 3.3 out of 5, averaged over 5 years.

Our resource can be used for clinical medical students in the third or fourth year. As patient safety is still taught with limited depth in GME, we feel that this resource can be used for interns and residents across disciplines. We would suggest adjusting the cases for this audience to reflect a more independent physician learner. We evaluated only knowledge recall and application of this knowledge to cases. We cannot comment on how this knowledge would translate to the clinical environment or to what extent it changed attitudes on the role of human behavior in a safety event. Our institution does not have a robust nursing school, so we do not have nursing or advanced practice provider learners to incorporate into the session. Some of our faculty are nurses and human resources professionals, as well as physicians. This approach is how we have addressed interprofessional learning in our environment.

One future approach in adapting the current design will be to provide a voice-over slide presentation of some or all of the lecture material prior to the session or to redesign the lecture into an interactive slide presentation. This prework, which would be active, would then be followed by the active small-group discussion. We are considering surveying fourth-year students to see the impact of this session on their attitudes and their retention of the content.

Conclusion

Just culture is an important concept to help physicians in training learn how to respond to safety events. The knowledge of how organizations create an accountable culture as part of high reliability is a foundation for safer delivery of care. This resource aids in teaching key just culture concepts and allows for a facilitated case-based discussion using realistic scenarios that a clinical student is likely to encounter. In a health care delivery science curriculum, it provides the safety science balance to concepts of malpractice, with which students may be more familiar.

Appendices

A. Slides for Cases.pptx

B. Lecture Slides.pptx

C. Faculty Guide.docx

D. Quiz and Evaluation Items.docx

All appendices are peer reviewed as integral parts of the Original Publication.

Brijen J. Shah, MD: Associate Dean for GME in Quality Improvement and Patient Safety, Departments of Medicine/Gastroenterology, Geriatrics, and Medical Education, Icahn School of Medicine at Mount Sinai; ORCID: https://orcid.org/0000-0002-5526-3241

Bonnie Portnoy, RN: Vice President for Risk Management and Patient Safety, Mount Sinai Health System

Dennis Chang, MD: Associate Professor of Medicine, Division of Hospital Medicine, Department of Medicine, Washington University School of Medicine in St. Louis

Marc Napp, MD: Deputy Chief Medical Officer, Mount Sinai Health System

Acknowledgments

We would like to thank Megan Sacco and Tiffany Moore for assisting in gathering data. We would also like to thank the more than 20 preceptors who have taught these sessions and provided invaluable feedback over the 4 years.

Disclosures

None to report.

Funding/Support

None to report.

Copyright © 2021 Shah et al. This is an open-access publication distributed under the terms of the Creative Commons Attribution license.

Ethical Approval

Reported as not applicable.

References

1. Marx D. *Patient Safety and the "Just Culture": A Primer for Health Care Executives.* Trustees of Columbia University; 2001.
2. Marx D. Patient safety and the just culture. *Obstet Gynecol Clin North Am*. 2019;46(2):239-245. https://doi.org/10.1016/j.ogc.2019.01.003
3. Institute for Healthcare Improvement Open School. *IHI Open School Online Courses: Curriculum Overview.* Institute for Healthcare Improvement; date unknown. Accessed April 5, 2021. http://www.ihi.org/education/IHIOpenSchool/Courses/Documents/Course%20Catalog.pdf
4. Barnsteiner J, Disch J. A just culture for nurses and nursing students. *Nurs Clin North Am*. 2012;47(3):407-416. https://doi.org/10.1016/j.cnur.2012.05.005
5. Walker D, Altmiller G, Barkell N, Hromadik L, Toothaker R. Development and validation of the Just Culture Assessment Tool for nursing education. *Nurse Educ*. 2019;44(5):261-264. https://doi.org/10.1097/NNE.0000000000000705
6. Mirza A, Winer J, Garber M, Makker K, Maraqa N, Alissa R. Primer in patient safety concepts: simulation case-based training for pediatric residents and fellows. *MedEdPORTAL*. 2018;14:10711. https://doi.org/10.15766/mep_2374-8265.10711
7. Bartlett CS, Huerta SA. Creating change: an experiential quality improvement and patient safety curriculum for medical students. *MedEdPORTAL*. 2018;14:10660. https://doi.org/10.15766/mep_2374-8265.10660
8. Szymusiak J, Fox MD, Polak C, et al. An inpatient patient safety curriculum for pediatric residents. *MedEdPORTAL*. 2018;14:10705. https://doi.org/10.15766/mep_2374-8265.10705
9. Cumbler E, Glasheen J. Teaching patient safety via a structured review of medical errors: a novel approach to educating residents about medical error, disclosure, and malpractice. *MedEdPORTAL*. 2007;3:761. https://doi.org/10.15766/mep_2374-8265.761
10. Broder JC, Doyle PA, Kelly L, Wald C. How we do it: operationalizing just culture in a radiology department. *AJR Am J Roentgenol*. 2019;213(5):986-991. https://doi.org/10.2214/AJR.19.21566
11. David DS. The association between organizational culture and the ability to benefit from "just culture" training. *J Patient Saf*. 2019;15(1):e3-e7. https://doi.org/10.1097/PTS.0000000000000561
12. Nie Y, Li L, Duan Y, et al. Patient safety education for undergraduate medical students: a systematic review. *BMC Med Educ*. 2011;11:33. https://doi.org/10.1186/1472-6920-11-33
13. *USMLE Content Outline*. United States Medical Licensing Examination; 2020. Accessed July 8, 2020. https://www.usmle.org/pdfs/usmlecontentoutline.pdf
14. Martinez W, Lehmann LS, Thomas EJ, et al. Speaking up about traditional and professionalism-related patient safety threats: a national survey of interns and residents. *BMJ Qual Saf*. 2017;26(11):869-880. https://doi.org/10.1136/bmjqs-2016-006284

Received: January 24, 2021
Accepted: April 30, 2021
Published: July 9, 2021

Copyright © 2021 Shah et al. This is an open-access publication distributed under the terms of the Creative Commons Attribution license.
